# Complete biosynthetic pathway to the antidiabetic drug acarbose

**DOI:** 10.1038/s41467-022-31232-4

**Published:** 2022-06-15

**Authors:** Takeshi Tsunoda, Arash Samadi, Sachin Burade, Taifo Mahmud

**Affiliations:** Department of Pharmaceutical Sciences, Oregon State University, Corvallis, OR 97331-3507 USA

**Keywords:** Biocatalysis, Biosynthesis, Amino sugars, Transferases, Natural products

## Abstract

Acarbose is a bacterial-derived α-glucosidase inhibitor clinically used to treat patients with type 2 diabetes. As type 2 diabetes is on the rise worldwide, the market demand for acarbose has also increased. Despite its significant therapeutic importance, how it is made in nature is not completely understood. Here, we report the complete biosynthetic pathway to acarbose and its structural components, GDP-valienol and *O*-4-amino-(4,6-dideoxy-α-D-glucopyranosyl)-(1→4)-*O*-α-D-glucopyranosyl-(1→4)-D-glucopyranose. GDP-valienol is derived from valienol 7-phosphate, catalyzed by three cyclitol modifying enzymes, whereas *O*-4-amino-(4,6-dideoxy-α-D-glucopyranosyl)-(1→4)-*O*-α-D-glucopyranosyl-(1→4)-D-glucopyranose is produced from dTDP-4-amino-4,6-dideoxy-D-glucose and maltose by the glycosyltransferase AcbI. The final assembly process is catalyzed by a pseudoglycosyltransferase enzyme, AcbS, which is a homologue of AcbI but catalyzes the formation of a non-glycosidic C-N bond. This study clarifies all previously unknown steps in acarbose biosynthesis and establishes a complete pathway to this high value pharmaceutical.

## Abstract (web summary)

The market demand for acarbose, a drug used for treatment of patients affected by type-2 diabetes, has increased. In this article, the authors report the acarbose complete biosynthetic pathway, clarifying previously unknown steps and identifying a pseudoglycosyltransferase enzyme, AcbS, a homologue of AcbI that catalyzes the formation of a non-glycosidic C-N bond.

## Introduction

Type 2 diabetes mellitus is on the rise worldwide with more than 463 million adults living with it in 2019^1^. This alarming trend is attributed to the increased level of obesity, physical inactivity, and poor diets among the world population. Among prescription medications used to treat type 2 diabetes are compounds called α-glucosidase inhibitors. These compounds inhibit sugar hydrolase enzymes that hydrolyze carbohydrates in the intestines, resulting in reduced glucose absorption and improved post-prandial blood sugar level^2^. The first α-glucosidase inhibitor approved in Europe and the U.S. for the treatment of type 2 diabetes was acarbose (**1**), a pseudo-oligosaccharide naturally produced by *Actinoplanes* sp. SE50/110 and several other soil bacteria^3–6^. The ‘noninferiority’ of **1** to metformin in terms of its glycated hemoglobin (HbA1c)-lowering effect, better lipid profile, and less postprandial hyperinsulinemia has made it a potential alternative to metformin for first-line treatment of type 2 diabetes^7,8^. As type 2 diabetes becomes more prevalent globally, the market demand for **1** has also increased. However, despite its significant therapeutic importance, how it is synthesized in bacteria is not completely understood.

**Fig. 1:**
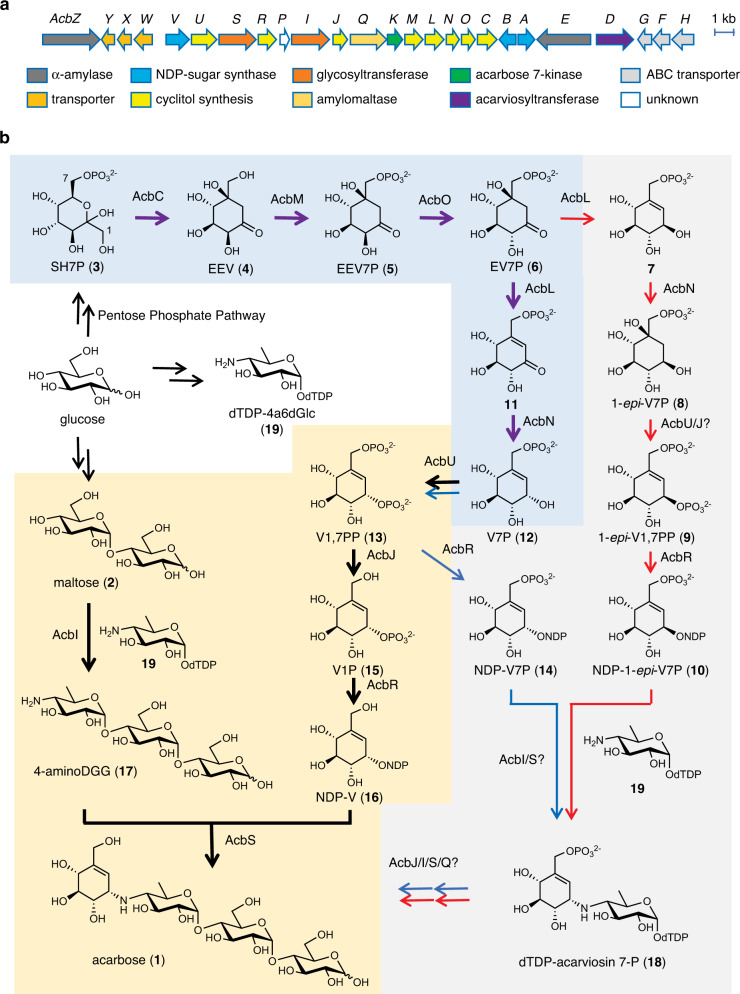
Acarbose biosynthesis in *Actinoplanes* sp. SE50/110. **a** Acarbose biosynthetic gene cluster from *Actinoplanes* sp. SE50/110; **b** Proposed acarbose biosynthetic pathways. The thick purple arrows (shaded in blue) indicate steps that have been characterized biochemically prior to this study. The blue and red arrows indicate previously proposed pathways. The thick black arrows (shaded in yellow) show the pathway elucidated in this study. SH7P, sedoheptulose 7-phosphate; EEV, 2-*epi*-5-*epi*-valiolone; EEV7P, 2-*epi*-5-*epi*-valiolone 7-phosphate; EV7P, 5-*epi*-valiolone 7-phosphate; V1P, valienol 1-phosphate; V7P, valienol 7-phosphate; 1-*epi*-V7P, 1-*epi*-valienol 7-phosphate; V1,7PP, valienol 1,7-diphosphate; 1-*epi*-V1,7PP, 1-*epi*-valienol 1,7-diphosphate; NDP-V, nucleoside diphosphate-valienol; NDP-V7P, nucleoside diphosphate-valienol 7-phosphate; NDP-1-*epi*-V7P, nucleoside diphosphate-1-*epi*-valienol 7-phosphate; dTDP-4a6dGlc, dTDP-4-amino-4,6-dideoxyglucose; 4-aminoDGG, *O*-4-amino-(4,6-dideoxy-α-D-glucopyranosyl)-(1→4)-*O*-α-D-glucopyranosyl-(1→4)-D-glucopyranose.

The chemical structure of acarbose (**1**) consists of a pseudosugar (C_7_-cyclitol), which is attached to an amino-deoxyhexose through a C-N bond, and maltose (**2**). The uncommon pseudosugar moiety and the experimental difficulties in handling highly hydrophilic intermediates have posed challenges to elucidating its mode of formation in nature. Although the biosynthesis of acarbose has been studied over the past four decades by isotope incorporation^9–13^, gene inactivation studies^14,15^, biochemical experiments^16–19^, as well as comparative genomic, transcriptomic, and proteomic analyses^20–22^, only the first half of the pathway has been elucidated (Fig. 1)^15^.

The C_7_-cyclitol unit of acarbose originates from 2-*epi*-5-*epi*-valiolone (EEV, **4**)^12^, a cyclization product of the pentose phosphate pathway intermediate sedoheptulose 7-phosphate (SH7P, **3**)^23^. This cyclization reaction is catalyzed by a dehydroquinate synthase (DHQS)-like enzyme AcbC^16^. In the SE50/110 strain, EEV (**4**) is converted to 2-*epi*-5-*epi*-valiolone 7-phosphate (EEV7P, **5**) by the ATP-dependent kinase AcbM^17^, and then to 5-*epi*-valiolone 7-phosphate (EV7P, **6**) by the epimerase AcbO^19^. It was suggested that the product is subsequently reduced by the putative cyclitol dehydrogenase AcbL to give 5-*epi*-valiolol 7-phosphate (**7**), followed by dehydration, phosphorylation, and nucleotidylation reactions to furnish NDP-1-*epi*-valienol 7-phosphate (**10**) (Fig. 1b, red arrows)^24^. However, a more recent study showed that 5-*epi*-valiolone 7-phosphate (**6**) is converted to valienone 7-phosphate (**11**) by AcbL and then reduced to valienol 7-phosphate (V7P, **12**) by AcbN^15^. What lies further downstream of the pathway is elusive.

On the basis of the putative functions of enzymes encoded by genes present in the biosynthetic gene cluster of acarbose in *Actinoplanes* sp. SE50/110 (Fig. 1a, Supplementary Table 1), it has been proposed that **12** is phosphorylated by the putative kinase AcbU to give valienol 1,7-diphosphate (V1,7PP, **13**) followed by modification by the putative nucleotidyltransferase AcbR to give NDP-valienol 7-phosphate (NDP-V7P, **14**)^15^. The latter compound is then coupled with dTDP-4-amino-4,6-dideoxyglucose (dTDP4a6dGlc, **19**)^25^ to give dTDP-acarviosin 7-phosphate (**18**). Finally, **18** is converted to acarbose (**1**) by the putative glycosyltransferases AcbI, AcbS, and/or AcbQ and the phosphatase AcbJ^15,24^. While the above-proposed pathway is attractive, no biochemical evidence exists to support it.

In this study, we elucidate step-by-step the biosynthetic pathway to acarbose (**1**) starting from V7P (**12**) and establish a complete map of the acarbose pathway, which is different from the proposed pathways described above.

## Results

### Valienol 1,7-diphosphate is involved in acarbose biosynthesis

**Fig. 2:**
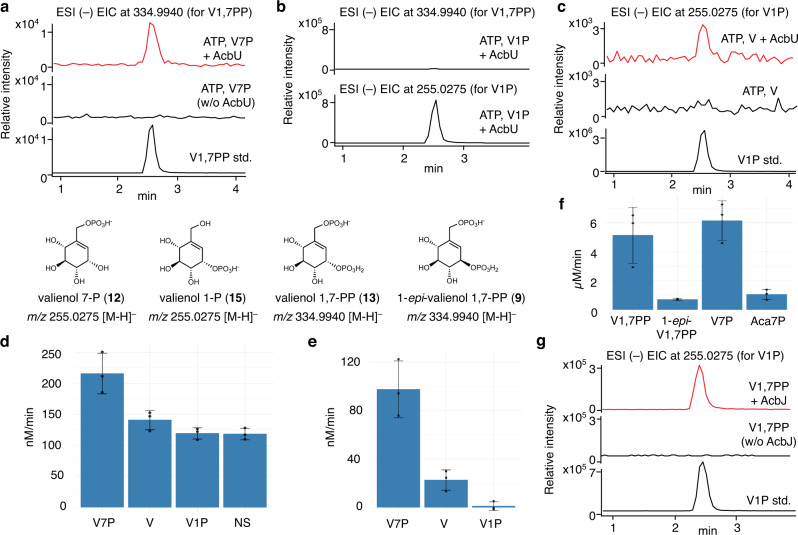
Biochemical characterization of the kinase AcbU and the phosphatase AcbJ. **a** ESI (–) EIC (*m/z* 334.9940) of AcbU reaction with V7P; **b** ESI (–) EIC (*m/z* 334.9940 and 255.0275) of AcbU reaction with V1P; **c** ESI (–) EIC (*m/z* 255.0275) of AcbU reaction with V; **d** PK/LDH coupled enzyme assay of AcbU reactions with V7P, V, or V1P as substrates, and no substrate (NS) in the presence of ATP. Reaction with boiled AcbU was used as a blank; **e** PK/LDH coupled enzyme assay of AcbU reactions with V7P, V, or V1P as substrates in the presence of ATP. Reactions of AcbU without substrate but with ATP were used as blanks; **f** MESG assay of AcbJ reaction with V1,7PP, 1-*epi*-V1,7PP, V7P, or Aca7P as substrates; **g** ESI (–) EIC (*m/z* 255.0275) of AcbJ reactions with V1,7PP. For **d**–**f**, error bars indicate standard deviation (SD) (*n* = 3 analytical replicates), and data are presented as mean values ± SD. All experiments were carried out at least three times with similar results. ESI, electrospray ionization mass spectrometry; EIC, extracted ion chromatogram; V, valienol; V1P, valienol 1-phosphate; V7P, valienol 7-phosphate; V1,7PP, valienol 1,7-diphosphate; 1-*epi*-V1,7PP, 1-*epi*-valienol 1,7-diphosphate; Aca7P, acarbose 7-phosphate.

Recent work by Bai and co-workers showed that V7P (**12**) is one of the intermediates in acarbose biosynthesis^15^. On the basis of its resemblance to a hexose 6-phosphate, V7P is predicted to be either phosphorylated to V1,7PP (**13**) or directly converted to valienol 1-phosphate (V1P, **15**)^15,26^. The former reaction resembles the conversion of fructose 6-phosphate to fructose 1,6-bisphosphate catalyzed by the 6-phosphofructose kinases, whereas the latter reaction resembles the conversion of glucose 6-phosphate to glucose 1-phosphate by the phosphoglucomutases^27^. The presence of multiple genes that encode putative cyclitol kinases, but none for phosphoglucomutases, in the *acb* cluster (Fig. 1a, Supplementary Table 1) suggests that V1,7PP is a more likely next intermediate. Two of the kinases, AcbK and AcbM, have been characterized to be an acarbose 7-kinase and a 2-*epi*-5-*epi*-valiolone 7-kinase, respectively^17,28^, leaving only the third putative kinase AcbU as the possible enzyme that may convert V7P to V1,7PP. To test this hypothesis, the putative kinase gene *acbU* was cloned in *Escherichia coli* and the recombinant protein (Supplementary Fig. 1) was incubated with ATP and V7P. V7P was prepared chemoenzymatically from valienol (V) using the cyclitol kinase enzyme ValC from the validamycin pathway (Supplementary Fig. 2)^15,29^. As expected, AcbU was able to efficiently convert V7P (**12**) to V1,7PP (**13**), indicating that AcbU is the next enzyme involved in the pathway (Fig. 2a). Incubation of AcbU with V1P (**15**) in the presence of ATP did not give any product (Fig. 2b), suggesting that AcbU can only phosphorylate the 1-OH, not the 7-OH. In addition, the study showed that AcbU can also convert valienol (V) to V1P (**15**), albeit with a lower yield (Fig. 2c).

### Substrate preference of AcbU

To confirm the substrate preference of AcbU, the enzyme’s kinase activities toward V7P (**12**), V, and V1P (**15**) were quantified using the pyruvate kinase/lactate dehydrogenase (PK/LDH) coupled enzyme assay^30^, with reactions using boiled AcbU as negative controls. Reactions were initiated by the addition of AcbU, and the oxidation of NADH to NAD^+^, which is stoichiometrically equivalent to the production of ADP, was measured by the decrease in UV absorption at 340 nm (A_340_) (Supplementary Figs. 3 and 4a). Interestingly, the PK/LDH coupled enzyme assay showed that not only reactions with a substrate (V7P, V, or V1P) but those without a substrate also produced ADP (Fig. 2d, Supplementary Table 5), suggesting that AcbU may also have an ATPase activity, making it a bifunctional enzyme that can function as a kinase as well as an ATPase. The ATPase activity does not seem to be due to a contaminating ATPase or a phosphatase, because, despite the use of purified recombinant AcbU, significantly high ATPase activity was observed (Fig. 2d, Supplementary Table 5). Coupled kinase/ATPase activity has been reported in ATP-binding cassette transporters^31^, rabbit skeletal muscle phosphorylase kinase^32^, human RIO2 serine/threonine-protein kinase/ATPase^33^, and enzymes from the GHKL ATPase/Kinase superfamily^34^. MS analysis of the reaction of AcbU without substrate, but containing ATP, confirmed the ATPase activity of AcbU (Supplementary Figs. 5 and 6, Supplementary Table 5). Re-examination of the kinase assay using a reaction mixture containing ATP but without substrate as a blank provided the net kinase activity of AcbU for V7P and V with the initial velocities of 98 ± 19 nM/min and 23 ± 7 nM/min, respectively (Fig. 2e, Supplementary Table 6), which suggest that V7P is a preferred substrate for AcbU to produce V1,7PP; no turnover was observed within experimental error for V1P as a substrate. AcbU can also phosphorylate valienol (V), but with the rate of only about 20% of that for V7P (Fig. 2e). Consistent with the MS data (Fig. 2b), the PK/LDH assay also showed that V1P is not a substrate of AcbU.

### Dephosphorylation of V1,7PP precedes nucleotidylation

There are two highly plausible scenarios that may take place after the formation of V1,7PP (**13**): (1) V1,7PP (**13**) is dephosphorylated to V1P (**15**) and then nucleotidylated to NDP-valienol (NDP-V, **16**) and (2) V1,7PP (**13**) is directly nucleotidylated to NDP-valienol 7-phosphate (NDP-V7P, **14**). The former scenario is based on the fact that both V1P and GDP-valienol are involved in the biosynthesis of validamycin A, a pseudo-trisaccharide produced by *Streptomyces hygroscopicus*. On the other hand, there is no precedent for the latter scenario (i.e., nucleotidylation of sugar diphosphates) but it has been proposed widely in the literature for acarbose biosynthesis^5,15,17,24^. To this end, we first investigated whether V1,7PP (**13**) may be converted to V1P (**15**) and then nucleotidylated to NDP-valienol (NDP-V, **16**). Bai and co-workers identified five phosphatases in *Actinoplanes* sp. SE50/110, including AcbJ from the *acb* cluster, that are able to dephosphorylate V7P (**12**) and 1-*epi*-V7P (**8**) to V and 1-*epi*-V, respectively^15^. However, both V and 1-*epi*-V were found to be shunt products and are not involved in acarbose biosynthesis. Yet, inactivation of *acbJ* in *Actinoplanes* sp. SE50/110 completely abolished the production of acarbose, indicating that AcbJ is critical in acarbose biosynthesis^15^, but its function in the pathway is enigmatic. To investigate if AcbJ can dephosphorylate V1,7PP (**13**), the recombinant enzyme was produced in *Streptomyces lividans* TK24 (Supplementary Fig. 1) and incubated with V1,7PP (**13**), and the phosphatase activity was measured by a purine nucleoside phosphorylase (PNP) assay using 7-methyl-6-thioguanosine (MESG) as a substrate (Fig. 2f, Supplementary Figs. 7 and 4b). In addition, AcbJ was incubated with V7P (**12**) as a positive control, as well as with 1-*epi*-V1,7PP (**9**) or acarbose 7-phosphate (Aca7P) (Supplementary Fig. 8). 1-*epi*-V1,7PP (**9**) and acarbose 7-phosphate were included because they were proposed to be involved in acarbose biosynthesis and a substrate for AcbJ, respectively^5,19,24^. The results showed that AcbJ efficiently dephosphorylated V7P (**12**) and V1,7PP (**13**) with the initial velocities of 6.1 ± 1.1 µM/min and 5.1 ± 1.6 µM/min, respectively, and poorly dephosphorylated 1-*epi*-V1,7PP (**9**) (0.69 ± 0.39 µM/min) and Aca7P (1.0 ± 0.3 µM/min) (Fig. 2g, Supplementary Fig. 8, Supplementary Table 7). The steady-state kinetic studies of recombinant AcbJ showed that the *K*_m_ for V1,7PP was 817 ± 168 µM and the *k*_cat_ was 22 ± 4 min^-1^ (Supplementary Fig. 9). Given that the dephosphorylation product of V7P (**12**) is not part of the acarbose pathway, the data suggest that AcbJ is involved in the pathway by dephosphorylating V1,7PP (**13**) to V1P (**15**). It is noteworthy that AcbJ only dephosphorylates the C-7 phosphate group, not the C-1 phosphate group, as incubation of AcbJ with V1P (**15**) did not give any product (Supplementary Fig. 10).

**Fig. 3:**
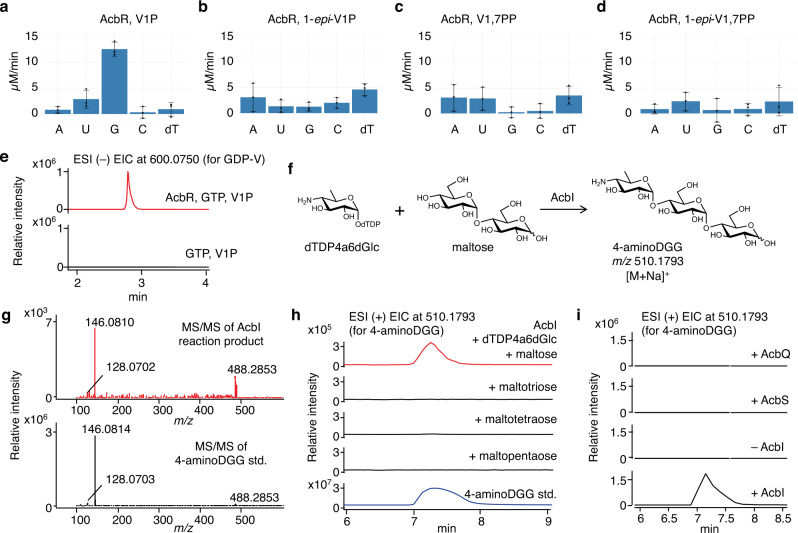
Biochemical characterizations of AcbR and AcbI. **a**–**d** The activity of AcbR with V1P, 1-*epi*-V1P, V1,7PP, or 1-*epi*-V1,7PP as substrates incubated with various NTPs. The blank was a reaction mixture lacking AcbR. A: ATP, U: UTP, G: GTP, C: CTP, dT: dTTP; **e** Partial LC-QTOF/MS EIC chromatograms (negative ion mode) of AcbR reaction with V1P and GTP (in red) and the negative control lacking AcbR (in black). Both chromatograms are the extraction of corresponding calculated exact mass for GDP-valienol (*m/z* 600.0750 [M-H]^–^). **f** Coupling between dTDP4a6dGlc and maltose catalyzed by AcbI; **g** MS/MS analysis of the product of the AcbI reaction; **h** ESI( + ) EIC for 4-aminoDGG (*m/z* 510.1793, [M + Na]^+^) from AcbI reactions with maltose, maltotriose, maltotetraose, and maltopentaose; and (**i**) ESI( + ) EIC for 4-aminoDGG (*m/z* 510.1793 [M + Na]^+^) from AcbQ, AcbS, and AcbI reactions with maltose. For **a**–**d**, error bars indicate standard deviation (SD) (n = 3 analytical replicates), and data are presented as mean values ± SD. All experiments were carried out at least three times with similar results. ESI, electrospray ionization mass spectrometry; EIC, extracted ion chromatogram; V1P, valienol 1-phosphate; V1,7PP, valienol 1,7-diphosphate; 1-*epi*-V1,7PP, 1-*epi*-valienol 1,7-diphosphate; A, ATP; U, UTP; G, GTP; C, CTP; dT, dTTP; dTDP-4a6dGlc, dTDP-4-amino-4,6-dideoxyglucose; 4-aminoDGG, *O*-4-amino-(4,6-dideoxy-α-D-glucopyranosyl)-(1→4)-*O*-α-D-glucopyranosyl-(1→4)-D-glucopyranose.

Next, we investigated the putative nucleotidyltransferase AcbR. This protein shows high similarity to VldB (63 % identity), which is the first characterized cyclitol nucleotidyltransferase that converts V1P to GDP-valienol (GDP-V) in validamycin biosynthesis^35^. While efforts to obtain recombinant AcbR from *E. coli* resulted in only an insoluble protein, expressing the gene in *S. lividans* TK24 gave a soluble enzyme (Supplementary Fig. 1). The recombinant AcbR was then incubated with V1P (**15**) in the presence of the nucleotidyl triphosphates, ATP, UTP, GTP, CTP, or dTTP. As expected, AcbR was able to efficiently convert V1P (**15**) to NDP-V (**16**), as determined by an enzymatic coupling assay using inorganic pyrophosphatase (IPP), PNP and MESG (Fig. 3a, Supplementary Fig. 11)^35^. Among the five nucleotidyl triphosphates tested, GTP was the preferred substrate, whereas UTP was also recognized as a substrate but with the rate of only about 25% of that for GTP (Fig. 3a, Supplementary Table 8). The formation of GDP-V was subsequently confirmed by LC-QTOF/MS (Fig. 3e). The steady-state kinetic studies of recombinant AcbR showed that the *K*_m_ for V1P was 412 ± 83 μM and the *k*_cat_ was 14 ± 1 min^-1^ (Supplementary Fig. 12). The enzyme was also incubated with 1-*epi*-V1P, V1,7PP, or 1-*epi*-V1,7PP. Both V1,7PP (**13**) and 1-*epi-*V1,7PP (**9**) have been proposed as potential substrates for AcbR (Fig. 1)^5,15,24^, and their conversion to NDP-V7P (**14**) or NDP-1-*epi*-V7P (**10**), respectively, would support the notion that nucleotidylation at C-1 takes place prior to dephosphorylation at C-7. While AcbR can also convert 1-*epi*-V1P, V1,7PP, and 1-*epi*-V1,7PP to their NDP derivatives, the conversion rates were significantly lower than that for V1P with GTP (Fig. 3a–e). Therefore, overall the results suggest that V1P (**15**) is the most likely substrate for AcbR, and the dephosphorylation at C-7 precedes the nucleotidylation at C-1.

### dTDP-acarviosins are not intermediates

It has been proposed that either dTDP-acarviosin 7-phosphate (**18**) or dTDP-acarviosin is an intermediate in acarbose biosynthesis^15,24,36^. These proposed compounds may be derived from a coupling reaction between dTDP-4-amino-4,6-dideoxyglucose (dTDP4a6dGlc, **19**)^25^ and GDP-V7P or GDP-V, respectively. However, as AcbR only effectively produces GDP-V, the involvement of **18** in the acarbose pathway may be ruled out. The question remains whether GDP-V can be coupled with dTDP4a6dGlc to produce dTDP-acarviosin. Although GDP-V is structurally different from a normal sugar donor (e.g., GDP-glucose), the coupling reaction is still possible, as GDP-V may function as a pseudosugar donor to an amino acceptor (an aglycon). This type of reaction may be catalyzed by pseudoglycosyltransferases (PsGTs), an emerging group of enzymes highly similar to glycosyltransferases (GTs)^37–40^. Unfortunately, to date it is still difficult to differentiate PsGTs from regular GTs on the basis of their amino acid sequences alone. Nevertheless, there are two putative GT genes in the *acb* biosynthetic gene cluster, *acbI* and *acbS*. Both *acbI* and *acbS* encode proteins that are similar to the bacterial glycogen/starch synthases (e.g., GlgA proteins). In contrast to GlgA proteins (size between 350–550 aa), both AcbI and AcbS are bigger in size (>700 aa). To investigate whether AcbI or AcbS can catalyze a coupling reaction between GDP-V and dTDP4a6dGlc, the genes were cloned and expressed in *E. coli* (Supplementary Fig. 1). The recombinant AcbI and AcbS were individually incubated with GDP-V and dTDP4a6dGlc, which was synthetically prepared from galactose^25^. The results showed that none of the reaction mixtures gave any products (Supplementary Fig. 13), suggesting that dTDP-acarviosin is not involved in acarbose biosynthesis.

### AcbI catalyzes the formation of 4-aminoDGG

As neither dTDP-acarviosin 7-phosphate nor dTDP-acarviosin may be an intermediate in the acarbose pathway, other plausible scenarios were explored. Early studies have shown that upon hydrogenation, acarbose can be converted to several cyclitol products (most prominently validatol) and a basic trisaccharide, *O*-4-amino-(4,6-dideoxy-α-D-glucopyranosyl)-(1→4)-*O*-α-D-glucopyranosyl-(1→4)-D-glucopyranose (4-aminoDGG, **17**)^41^. While the latter compound has never been isolated from the cultures of acarbose producing bacteria, the possibility of its involvement in the acarbose pathway cannot be ruled out. 4-AminoDGG (**17**) may be formed from a coupling between dTDP4a6dGlc (**19**) and maltose through a glycosyltransferase reaction. Alternatively, it may also be formed from maltotriose or longer oligosaccharides by transferring a maltose unit to dTDP4a6dGlc (**19**)^11^. To test these possibilities, we incubated the putative GT AcbI with dTDP4a6dGlc (**19**) and maltose, maltotriose, maltotetraose, or maltopentaose. The reaction products were then analyzed by LC-MS. To our delight, AcbI was able to catalyze the coupling between dTDP4a6dGlc (**19**) and maltose to give 4-aminoDGG (**17**) (Fig. 3g–h), whereas reactions containing maltotriose, maltotetraose, or maltopentaose did not give any product. The conversion yield of 4-aminoDGG (**17**) was about 23 ± 2% after 3 h incubation under the experimental conditions (Supplementary Fig. 14a). Moreover, we found that the reaction is unique to AcbI, as incubations of dTDP4a6dGlc (**19**) and maltose (**2**) with the second putative GT AcbS as well as with AcbQ, a putative amylomaltase/acarbose 4-α-glucanotransferase, did not give any product (Fig. 3i).

### AcbS is a PsGT

**Fig. 4:**
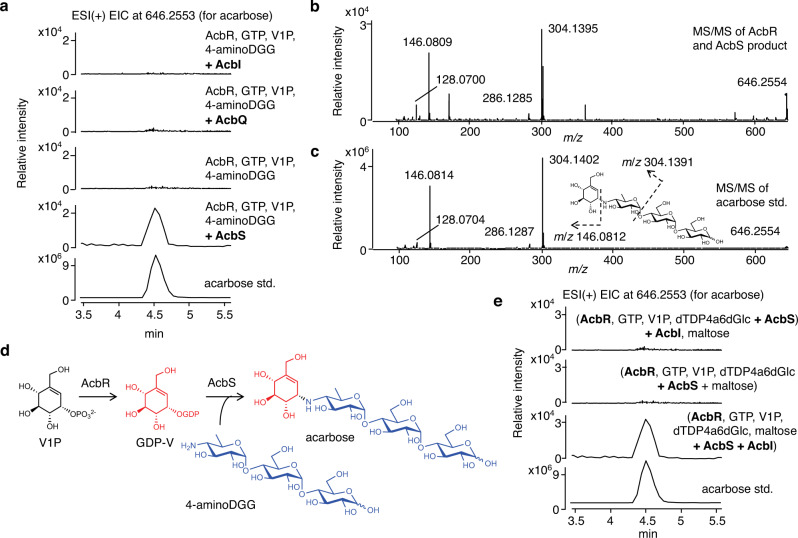
Characterization of the pseudoglycosyltransferase AcbS. **a** ESI( + ) EIC for acarbose at *m/z* 646.2553 of reaction mixtures with AcbI, AcbQ, or with or without AcbS; **b** ESI-MS/MS of the AcbS product; **c** ESI-MS/MS of acarbose standard; **d** reaction scheme of AcbR and AcbS; **e** ESI( + ) EIC for acarbose at *m/z* 646.2553 of reaction mixtures with AcbI, AcbR, and AcbS added in different sequence and combination (see text and experimental section). All experiments were carried out at least three times with similar results. ESI, electrospray ionization mass spectrometry; EIC, extracted ion chromatogram; V1P, valienol 1-phosphate; GDP-V, GDP-valienol; dTDP-4a6dGlc, dTDP-4-amino-4,6-dideoxyglucose; 4-aminoDGG, *O*-4-amino-(4,6-dideoxy-α-D-glucopyranosyl)-(1→4)-*O*-α-D-glucopyranosyl-(1→4)-D-glucopyranose.

With GDP-V and 4-aminoDGG (**17**) found to be involved in acarbose biosynthesis, what is left in the pathway is the assembly of acarbose from these two structural components. Because AcbI catalyzes the formation of 4-aminoDGG (**17**), AcbS became the most likely candidate for this final reaction. As expected, incubation of recombinant AcbS with GDP-V (generated in situ from V1P and GTP by AcbR) and 4-aminoDGG did produce acarbose (Fig. 4). The product was confirmed by direct comparisons of its ESI-MS/MS spectrum with that of an acarbose standard (Fig. 4b–c). The conversion yield of acarbose was estimated to be 20 ± 3% after 3 h incubation under the experimental condition (Supplementary Fig. 14b). AcbI and AcbQ were also incubated with GDP-V and 4-aminoDGG, but no product was observed (Fig. 4a), indicating that AcbS is the only PsGT enzyme in the pathway that catalyzes the formation of a non-glycosidic C-N bond between GDP-V and 4-aminoDGG (Fig. 4d).

Next, we reconstituted the pathway from V1P (**15**) to acarbose using recombinant AcbI, AcbR, and AcbS in a one-pot reaction. As expected, incubation of the three enzymes in the presence of V1P (**15**), GTP, dTDP4a6dGlc (**19**), maltose (**2**) and the necessary cofactors for 6 h did furnish acarbose (Fig. 4e). Reaction mixtures that lacked AcbI did not give any products, confirming that AcbS cannot complement the AcbI activity. Furthermore, addition of AcbI to the reaction mixture after the removal of AcbR and AcbS also did not give any products, confirming the sequence of AcbI–AcbS reactions as shown in Fig. 1 (shaded in yellow). In addition, the results further support the notion that dTDP-acarviosin 7-phosphate (**18**) and dTDP-acarviosin are not intermediates in the acarbose pathway.

### AcbS is a dedicated PsGT

Although AcbS has been found to be a PsGT enzyme, the possibility that it can also catalyze a GT reaction cannot be ruled out. To investigate if AcbS is a dedicated PsGT or a bifunctional GT/PsGT, we incubated the recombinant enzyme with GDP-glucose (as the sugar donor) and 4-aminoDGG (as the sugar acceptor) (Supplementary Fig. 15a). Structurally, GDP-glucose is the closest sugar analogue of GDP-V. Analysis of the reaction mixture by LC-MS showed the lack of the expected product G-N-DGG (Supplementary Fig. 15b), suggesting that AcbS is not able to use a regular NDP-sugar (GDP-glucose) as substrate. This was further supported by the apparent lack of consumption of GDP-glucose in the reaction (Supplementary Fig. 15c). Next, we incubated AcbS with GDP-valienol and maltotriose (in place of 4-aminoDGG) (Supplementary Fig. 16a). The reaction also did not give the expected product valienol-maltotriose (VGGG) (Supplementary Fig. 16b) and showed no noticeable consumption of maltotriose (Supplementary Fig. 16c), suggesting that the amino group in 4-aminoDGG is essential for the coupling reaction to occur. The need of a strong nucleophile (*i.e*., an amino group) in the AcbS reaction is similar to that of VldE from the validamycin pathway where validamine 7-P but not validol 7-P can function as an acceptor^39^. In fact, all natural pseudo-oligosaccharides identified so far contain a pseudo-anomeric C-N bond^42^.

### AcbS homologues are ubiquitous in nature

**Fig. 5:**
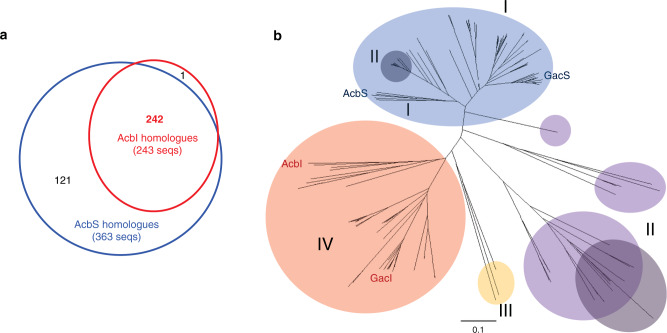
Homologues of AcbI and AcbS. **a** Homology searches for AcbI and AcbS; **b** Phylogenetic tree of AcbI and AcbS. The ones shaded in blue are predicted to be AcbS homologues (PsGTs). The ones shaded in red are AcbI homologues (GTs). The ones shaded in dark blue are AcbS whose genes do not have an AcbI partner gene within a biosynthetic gene cluster (BGC). The ones shaded in purple do not pair with another AcbI/AcbS homologue in a BGC. The ones shaded in dark purple are AcbI/AcbS homologues whose genes are part of a BGC that lacks an aminotransferase gene. The ones shaded in yellow are AcbI/AcbS homologue whose genes are part of a BGC that does not contain cyclitol biosynthetic genes.

Although both AcbS (a PsGT) and AcbI (a GT) are similar to glycogen/starch synthases (GTs family-5), their amino acid sequences are somewhat unique and differentiable from those of the glycogen/starch synthases. BLAST analysis of the NCBI database using the AcbS and the AcbI sequences resulted in 363 and 243 homologous proteins, respectively. However, as AcbS and AcbI are also homologous to each other (42.5% identity), almost all of the proteins identified from the AcbI homology search (242 out of 243 proteins) were also identified in the AcbS search (Fig. 5a). Only one of the AcbI homologues was not found in the AcbS search. This outlying protein is the hypothetical protein A3B44_01620 from a *Candidatus Levybacteria* bacterium. Phylogenetic analysis of the 242 sequences similar to both AcbS and AcbI provided two distinct major clades (Fig. 5b, shaded in blue and red) and a number of smaller clades (shaded in purple and yellow). The ones shaded in blue are predicted to be AcbS homologues (PsGTs), as they are clustered together with AcbS. Among them is GacS, which was found in the acarbose biosynthetic gene cluster in *Streptomyces glaucescens* GLA-O^5^. The ones shaded in red are AcbI homologues (GTs), as they are clustered together with the characterized AcbI. Except for a small number of proteins that formed a sub-clade (shaded in dark blue), the gene of each member of the AcbS major clade was found together with an AcbI homologue gene within a biosynthetic gene cluster (BGC). Those BGCs also contain a sugar phosphate cyclase gene (similar to *acbC*), a cyclitol nucleotidyltransferase gene (similar to *acbR*), and other cyclitol (pseudosugar) biosynthetic genes, indicating that they are involved in the biosynthesis of acarbose or related pseudo-oligosaccharides. Moreover, they are distributed across 9 genera of bacteria, indicating that pseudo-oligosaccharides such as acarbose are produced by a wide variety of bacteria.

The genes of the AcbS/AcbI homologues that formed smaller clades (shaded in purple) do not pair with another AcbI/AcbS homologue gene in a BGC. However, these BGCs contain pseudosugar biosynthetic genes such as *acbC* and *acbR*, indicating that the AcbS/AcbI homologues in those clades are PsGTs and they may be involved in the biosynthesis of different types of pseudo-oligosaccharides. Interestingly, the AcbS homologues in those clades appear to be more similar to members of the GT family-1 than those of the GT family-5. Considering that the PsGT VldE from the validamycin pathway is similar to members of the GT family-20 and AcbS from the acarbose pathway is similar to members of the GT family-5, the putative PsGTs that are similar to members of the GT family-1 may represent yet another uncharacterized family of PsGTs involved in the biosynthesis of secondary metabolites^26,43,44^. Moreover, there are a number of AcbS/AcbI homologues that are not paired with another AcbI/AcbS homologue and the BGCs do not contain *acbC* and *acbR* genes (shaded in yellow), indicating that they are more similar to AcbI than AcbS and may not be involved in the biosynthesis of pseudo-oligosaccharides.

Most putative pseudo-oligosaccharide BGCs described in this report contain an AcbV homologue, an aminotransferase enzyme that introduces an amino group to the pseudosugar acceptors, underscoring the importance of an amino group in the PsGT coupling reaction^39^. However, BGCs containing AcbS homologous genes from one of the smaller clades (shaded in dark purple) do not contain an aminotransferase gene. The lack of an aminotransferase gene in those BGCs may be interpreted in several different ways including (1) there is a housekeeping aminotransferase that can complement the role of AcbV in those pathways; (2) the pseudosugar acceptor molecules are produced by another pathway or from one of the aminosugars available in the cell (e.g., glucosamine, kanosamine, galactosamine); and (3) the putative PsGTs do not require an amino group for the coupling reaction. The latter possibility is interesting from the point of view of the catalytic mechanisms underlying the coupling reactions catalyzed by PsGTs.

## Discussion

Owing to its potent α-glucosidase inhibitory activity, the pseudo-oligosaccharide acarbose has been used clinically to treat patients with type 2 diabetes. This natural glycomimetic possesses potent competitive inhibitory activity against various sugar hydrolases including sucrase, maltase, glucoamylase, and other human intestinal α-glucosidases^45^. As a member of the emerging family of the C_7_N-aminocyclitol natural products^43,44^, acarbose contains a unique C_7_-pseudosugar (cyclitol or carbasugar) moiety in its structure, which attributes to the enhanced binding of the compound to the hydrolases. Other members of the C_7_N-aminocyclitol family containing the same pseudosugar unit include the amylostatins, the oligostatins, the trestatins, and the validamycins^42,45^. Due to it potent inhibitory activity against trehalases, validamycin A has been used widely to control fungal infections in rice plants.

Despite the importance of acarbose as a clinically used drug, how this bioactive compound is synthesized in the producing organisms was not completely understood. Therefore, elucidation of its complete biosynthetic pathway is important from both scientific and industrial points of view. In this study, we established the complete pathway to acarbose in *Actinoplanes* sp. SE50/110 by systematically characterizing the biochemical functions of enzymes involved in the pathway starting from V7P all the way to the final product. In addition to the five enzymes previously identified for the early steps of the pathway, we characterized five additional proteins that are essential for the formation of the structural components of acarbose and their assembly. They are the V7P kinase AcbU, the V1,7PP phosphatase AcbJ, the V1P nucleotidyltransferase, the glycosyltransferase AcbI, and the pseudoglycosyltransferase AcbS. While the sixth enzyme, AcbQ, a putative amylomaltase, is not involved in the formation of acarbose, it may play a role in the modification of acarbose. In fact, in addition to acarbose, longer pseudo-oligosaccharides are produced by acarbose-producing bacteria^4,45^.

It is noteworthy that most of the proteins involved in acarbose biosynthesis are similar to enzymes that are involved in sugar biosynthesis, e.g., sugar kinases and sugar phosphatases. Those enzymes may have evolved from sugar biosynthetic enzymes with a new capability to recognize cyclitols (pseudosugars) as substrates. The acarbose 7-kinase AcbK and the cyclitol 7-kinase AcbM are highly similar to the adenosine kinases and the ROK family proteins, respectively^18,28^. In validamycin biosynthesis, the valienone kinase ValC is also similar to the ROK family proteins^29^, whereas the validoxylamine 7´-phosphate phosphatase VldH is similar to trehalose 7-phosphate phosphatase (a HAD family sugar phosphatase)^37^. Therefore, it is understandable that enzymes similar to sugar kinases and phosphatases, such as AcbU and AcbJ, respectively, are involved in the acarbose pathway. However, the two-step conversion of V7P to V1P catalyzed by AcbU and AcbJ is somewhat peculiar, as switching a phosphate group from C-7 to C-1 of valienol may be done more efficiently and in a single step by a phosphoglucomutase-like enzyme. However, as V7P is not a sugar, some enzymes such as phosphoglucomutases may have not evolved to obtain the ability to recognize pseudosugars as substrates, or at least not in the acarbose producing bacterium *Actinoplanes* sp. SE50/110.

As observed with the kinases and the phosphatases, the nucleotidyltransferase AcbR is also similar to sugar nucleotidyltransferases, but functions as a cyclitol nucleotidyltransferase. The first known cyclitol nucleotidyltransferase is ValB or VldB, which catalyzes the conversion of V1P to GDP-V in the validamycin pathway^35,37^. In fact, both AcbR and ValB are similar to glucose 1-phosphate adenylyltransferases. In the literature and protein databases, AcbR has been widely denoted as a putative 1-*epi*-valienol-1,7-bisphosphate-1-adenylyltransferase. However, on the basis of the results from this study, the enzyme should be renamed valienol 1-phosphate nucleotidyltransferase.

Although the glycosyltransferase AcbI is functionally different from glycogen/starch synthases (e.g., GlgA proteins), it does catalyze a reaction that is similar to that of glycogen/starch synthases. However, instead of using NDP-glucose, AcbI uses dTDP-4-amino-6-deoxyglucose as a sugar donor. The identification of maltose as a sugar acceptor is consistent with the expected final product, but is somewhat contradictory to the result of a previous study using radiolabeled compounds that showed maltotriose was the source of the maltose moiety in acarbose^11^. However, it is possible that the radiolabeled maltotriose was converted to maltose in the culture before being incorporated into acarbose.

The ability of AcbS to catalyze the coupling between GDP-V and 4-aminoDGG to furnish acarbose makes it the second PsGT enzyme characterized biochemically, with VldE from the validamycin pathway being the first^37,38^. In contrast to VldE, which is similar to trehalose 6-phosphate synthase (GT family-20), AcbS has high homology to members of GT family-5, which include glycogen/starch synthases, α-1,3-glucan synthases, and α-1,4-glucan synthases^46^. All of these enzymes are known to be retaining GTs that adopt GT-B 3D structures. This is consistent with the coupling between GDP-V and 4-aminoDGG by AcbS that also gives a product with a retained anomeric configuration. Interestingly, bioinformatic studies showed that some AcbS homologues are more similar to members of the GT family-1 than the GT family-5. Members of the GT family-1 are fundamentally different from those from the GT family-5 because they catalyze glycosylation reaction with inversion of configuration. Many of them are involved in natural products biosynthesis, e.g., UDP-glucuronosyltransferases, anthocyanidin 3-*O*-glucosyltransferases, zeatin *O*-β-xylosyltransferases, and UDP-Glc: cinnamoyl *O*-β-glucosyltransferases. Detailed studies of the AcbS homologues from this clade of PsGTs may lead to the discovery of new natural products and/or biocatalysts.

Despite the similarities of AcbI and AcbS, they catalyzed two fundamentally different reactions. AcbI catalyzes the formation of a glycosidic bond (a GT reaction) and AcbS catalyzes the formation of a non-glycosidic C-N bond (a PsGT reaction). Inspection of the genome sequences of bacteria containing AcbS and/or AcbI homologues in the NCBI database led to the identification of biosynthetic gene clusters similar to the *acb* cluster in various bacteria, suggesting that all of these bacteria can produce acarbose or related compounds. This raises interesting physiological and/or ecological questions as to why bacteria need to produce acarbose or related pseudo-oligosaccharides. Currently, there are two hypotheses pertaining to the natural function of acarbose. First, acarbose may play a role as a ‘carbophore’ which shuttles sugars in and out between intra- and extracellular spaces of bacteria (Supplementary Fig. 17)^24^. Second, acarbose and other pseudo-oligosaccharides, which have strong inhibitory activity towards sugar hydrolases, may be utilized for niche protection by inhibiting carbohydrate-degrading enzymes of other organisms^24,42^. What exactly the functions of pseudo-oligosaccharides are for the producing organisms in their natural environment remain an exciting topic to be explored.

## Methods

### General

All chemicals were obtained either from Sigma Aldrich, EMD, TCI, or Pharmacia. All reactions were carried out under an inert, Argon atmosphere in oven-dried glassware at 170 °C unless indicated otherwise. Methylene chloride (CH_2_Cl_2_) and triethylamine (Et_3_N) were distilled over calcium hydride prior to use. All other reagents and solvents were used without further purification from commercial sources. Thin-layer chromatography (TLC) was performed using silica gel plates (60 Å), which were visualized using a UV lamp, ceric ammonium molybdate (CAM), potassium permanganate, iodine, and/or vanillin stains. Chromatographic purification of products was performed on silica gel (60 Å, 72–230 mesh). NMR spectra were recorded on Bruker 500 or 700 MHz spectrometers. Proton and carbon chemical shifts are reported in ppm (δ) relative to the residual solvent signals as the internal standard. NMR data were processed using Bruker Topspin (version 3.5) or MNova (version 6.0.2). High-resolution ESI mass spectra were obtained using an Agilent 1260 HPLC upstream of an Agilent 6545 Q-ToF. The data were processed on Agilent MassHunter workstation (version 10.1). HPLC was performed using a Shimadzu dual LC-20AD solvent delivery system with a Shimadzu SPD-M20A UV/vis photodiode array detector.

### Substrate preparation

The substrates V, V1P, and 1-*epi*-V1P were synthesized from tetrabenzylglucose according to the procedure described in our previous publication^35^. V7P was prepared chemoenzymatically from V and ATP using the cyclitol kinase ValC (see the details below)^29^. V1,7PP and 1-*epi*-V1,7PP were synthesized from tetrabenzyl-1-*epi*-valienol following the procedures described in our previous publication and confirmed by NMR (see the details in Supplementary Figs. 19 and 20 and the accompanying information)^35^. Acarbose 7-phosphate was prepared from acarbose and ATP using the acarbose kinase AcbK (Supplementary Fig. 18) (see the details below)^28^. dTDP4a6dGlc was synthesized from galactose as described in Bowers et al.^25^. 4-aminoDGG was a gift from H. G. Floss at the University of Washington and was confirmed by NMR (Supplementary Figs. 21 and 22).

### Strains, cultivation, and genetic manipulation

Genomic DNA (*g*DNA) of *Actinoplanes* sp. SE50/110 was prepared using GeneElute Bacterial Genomic DNA Kit (Sigma). *E. coli* strains DH10b, BL21(DE3)/*pLysS*, and ET12567/pUZ8002 were used as hosts for DNA manipulation, recombinant protein production, intergeneric conjugation between *E. coli* and *Streptomyces lividans* TK24, respectively (Supplementary Table 2). DNA fragments were recovered from an agarose gel using the E.Z.N.A. Gel-extraction kit (Omega Bio-tek). Restriction endonucleases were purchased from New England Biolabs. Preparation of plasmid DNA was done by using an EconoSpin (Epoch Life Science). All other DNA manipulations were performed according to standard protocols. PCR was performed in 35 cycles using PrimeSTAR GXL DNA polymerase or PrimeSTAR MAX DNA polymerase (Takara Bio). Oligodeoxyribonucleotides for PCR primers were synthesized by Sigma. The nucleotide sequences of the gene fragments were determined at the Center for Genome Research and Biocomputing Core Laboratories, Oregon State University. BD Difco LB medium was used for the *E. coli* culture. YEME medium was used for the *S. lividans* culture. YEME medium contains yeast extract (0.3%), Bacto peptone (0.5%), malt extract (0.3%), D-glucose (1.0%), sucrose (34%) and MgCl_2_ (5 mM).

### Construction of the expression vectors

The genes *acbU, acbJ, acbR, acbI, acbS* and *acbK* were PCR amplified from *g*DNA of *Actinoplanes* sp. SE50/110 using their corresponding primers (Supplementary Table 3). The amplicons were digested with appropriate restriction enzymes (Supplementary Table 2) and ligated with pRSET-B (Thermo Fisher), pXY201^47^, or pET28b (Novagen) vectors. All plasmids were listed in Supplementary Table 4. The plasmid pRSETB-*valC* was constructed by Minagawa et al.^29^.

### Construction of the expression systems

Plasmids pRSETB-*acbU*, -*acbI*, -*acbS* and -*valC* and pET28b-*acbK* were introduced into *E. coli* BL21(DE3)/pLysS using the normal transformation method for *E. coli*. Transformants were selected on the LB plates including ampicillin (100 µg/mL) and chloramphenicol (35 µg/mL) or kanamycin (50 µg/mL) and chloramphenicol (35 µg/mL) (for pET28b-*acbK*) and used for expression. Plasmids pXY201-*acbJ* and -*acbR* were introduced into *E. coli* ET12567/pUZ8002 for conjugation. These plasmids were introduced into *S. lividans* TK24 using the normal conjugation method for *Streptomyces*. The exconjugants were selected on the MS plates (defatted soy flour 2%, mannitol 2%, agar 2%), containing nalidixic acid (25 µg/mL) and apramycin (50 µg/mL), and used for getting recombinant enzymes.

### Preparation of recombinant AcbJ and AcbR

Transformants harboring *acbJ* or *acbR* were cultivated on the MS agar containing apramycin (50 µg/mL) until spores were formed. Subsequently, the bacteria (from a 1 cm×1 cm agar piece) were incubated in YEME medium (100 mL) containing apramycin (50 µg/mL). After 24 h of cultivation at 30 °C in a shaker (200 rpm), thiostrepton (10 µg/mL) was added and the cultures were incubated for 2 additional days (30 °C, 200 rpm). The bacteria were harvested by centrifugation (2850x *g*, 4 °C, 20 min) and the bacterial pellets were washed with buffer A (30 mM HEPES pH 8.0, 300 mM NaCl, 10% glycerol, 1 mM DTT). Then, the pellets were frozen with dry ice-acetone or liquid nitrogen and stored at –80 °C until use.

To prepare the recombinant proteins, the cells were resuspended with buffer A and treated with lysozyme at 4 °C for 30 min. After sonication (2 W, 15 sec, 10 times), the samples were centrifuged (12500 x g, 30 min, 4 °C) to remove the cell debris and the supernatants were incubated with 0.5 mL or 1.0 mL of TALON resin (Takara Bio) for 30 min at 4 °C. Each mixture was transferred into a column and the resin was washed with buffer A (20 mL) followed by buffer A (10 mL or 20 mL) containing imidazole (20 mM) to remove impurities from the resin. The recombinant proteins AcbR or AcbJ were eluted with buffer A (2 mL or 5 mL) containing imidazole (200 mM). The eluants were passed through a PD-10 column (GE healthcare) pre-equilibrated with the reaction buffer (Tris-HCl, pH 7.5, 20 mM). Recombinant proteins were concentrated with Amicon Ultra (molecular weight cut off (MWCO) 10 K, Merck Millipore) and concentration was confirmed with the Bradford assay (Bio-Rad).

### Preparation of recombinant AcbI, AcbK, AcbS, AcbU, and ValC

Transformants harboring *acbI*, *acbK*, *acbS*, *acbU*, or *valC* genes were grown on LB agar plates containing ampicillin (100 µg/mL) and chloramphenicol (35 µg/mL) (for pRSETB-*acbU*, -*acbI*, -*acbS* and -*valC*) or kanamycin (50 µg/mL) and chloramphenicol (35 µg/mL) (for pET28b-*acbK*) until colonies were formed. The colonies were picked and cultivated in 3–5 mL of LB medium containing appropriate antibiotics. After 12–14 h of cultivation at 37 °C in a shaker (200 rpm), the cultures (1 mL) were transferred to fresh LB medium (100 mL) containing appropriate antibiotics and shaken (37 °C, 200 rpm) until OD_600_ reached 0.4–0.5. After cooling to 4 °C on ice, IPTG (100 µM) was added, and the cultures were continued for 16–20 h at 16 °C. The cells were harvested by centrifugation (3200 × *g*, 4 °C, 10 min), and the cell pellets were washed with buffer A. Subsequently, the pellets were frozen with dry ice-acetone or liquid nitrogen and stored at –80 °C until use.

To prepare the recombinant proteins, the pellets were resuspended with buffer A, sonicated (2 W, 10 sec, 10 times), and then centrifuged (12,500 × *g*, 30 min, 4 °C) to remove the cell debris. Ni-NTA resin (UBPBio) was used to purify the recombinant proteins. Recombinant proteins were concentrated with Amicon Ultra (MWCO 10 K or 30 K) and concentration was confirmed with the Bradford assay.

### LC-Q-TOF/MS analysis

High-resolution mass spectrometry (HR-MS) was performed with an Agilent 1260 HPLC upstream of an Agilent 6545 Q-ToF. The separation was performed on an InfinityLab Poroshell HILIC column (150 × 4.6 mm, 2.7 μm) using the following isocratic (method A) or gradient (method B) solvent systems at a flow rate of 0.4 mL/min. Solvent A was aqueous ammonium formate solution (10 mM), and Solvent B was acetonitrile. The Q-ToF mass spectrometer was operated in an auto MS/MS mode or a targeted MS/MS mode. For method A, the column was pre-equilibrated with 50% A/ 50% B. Upon injection, the mobile phase composition was maintained 50% A/ 50% B for 10 min. For method B, the column was pre-equilibrated with 10% A/ 90% B. Upon injection, the mobile phase composition was maintained for 2 min, and then the mobile phase was changed using a linear gradient to 90% A/10% B over the following 20 min.

### Preparation of valienol 7-phosphate

Valienol 7-phosphate was prepared by phosphorylation of synthetically prepared valienol using the cyclitol kinase ValC (Supplementary Figs. 2, 23–25)^29^. The reaction mixture (50 µL) containing Tris-HCl (20 mM, pH 7.5), MgCl_2_ (5 mM), ATP (6 mM), valienol (5 mM) and ValC (5 µM) was incubated at 30 °C for 6 h. The enzyme was removed by ultrafiltration using a Pierce™ protein concentrator (MWCO: 3 K, Thermo Scientific) at 4 °C, 13,000 × *g*, 10 min, and the conversion ratio was assessed by a coupling enzymatic assay using pyruvate kinase (PK) and lactose dehydrogenase (LDH). For the PK/LDH assay, the ValC product solution (3 µL) was added to the assay mixture (100 µL) containing Tris-HCl (pH 7.5, 20 mM), PK (37 U/mL), LDH (46 U/mL), MgCl_2_ (5 mM), PEP (1.5 mM), and NADH (500 µM). The mixture was incubated at 30 °C for 30 min and the A_340_ was measured by SpectraMax iD3 plate reader (Molecular Devices) with the SoftMax Pro (version 7) processor. The absorption values were transferred into concentrations based on a calibration curve made of ADP standard. Data were analyzed with Microsoft Excel (version 16.54).

### In vitro reaction of AcbU

To get the initial velocity of AcbU, a reaction mixture (42 µL) containing Tris-HCl (20 mM, pH 7.5), MgCl_2_ (5 mM), ATP (500 µM), PK (37 U/mL), LDH (46 U/mL), PEP (1.5 mM), NADH (600 µM) and valienol (200 µM), valienol 7-phosphate (200 µM) or valienol 1-phosphate (200 µM) were preincubated at 30 °C and the A_340_ was monitored until no changes were observed. Subsequently, AcbU (0.5 µM) and Tris-HCl buffer were added to make a 50 µL reaction mixture. The change in A_340_ was monitored at 30 °C for 10 min.

For LC-QTOF/MS analysis, a reaction mixture (10 µL) containing Tris-HCl (pH 7.5, 20 mM), MgCl_2_ (1 mM), ATP (1 mM), AcbU (5 µM) and valienol (1 mM), valienol 7-phosphate (1 mM) or valienol 1-phosphate (1 mM) was each incubated at 30 °C for 3 h and then subjected to MS analysis. The above-described method A was used to analyze V1P, V1,7PP and V7P and method B was used to analyze valienol.

### Preparation of acarbose 7-phosphate

Acarbose 7-phosphate was prepared enzymatically from acarbose and ATP using the acarbose kinase AcbK (Supplementary Figs. 18, 26–28). A reaction mixture (50 µL) containing Tris-HCl (20 mM, pH 7.5), MgCl_2_ (5 mM), ATP (6 mM), acarbose (5 mM) and AcbK (5 µM) was incubated at 30 °C for 6 h. The enzyme was removed by ultrafiltration using a Pierce™ protein concentrator (MWCO 3 K) at 4 °C, 13,000 × *g*, 10 min, and the conversion ratio was assessed by the same method as employed for the preparation of V7P.

### In vitro reaction of AcbJ

To get the initial velocity of AcbJ, a reaction mixture (45 µL) containing Tris-HCl (pH 7.5, 20 mM), purine nucleoside phosphorylase (PNP) (2 U/mL), 7-methyl-6-thioguanosine (MESG, Setareh biotech) (600 µM) and V7P (500 µM), V1,7PP (500 µM), 1-*epi*-V1,7PP (500 µM) or acarbose 7-phosphate (500 µM) were preincubated at 30 °C and the A_360_ was monitored until no changes were observed. Subsequently, AcbJ (1.0 µM) and Tris-HCl buffer were added to make a 50 µL reaction mixture. The change in A_360_ was monitored at 30 °C for 10 min.

For LC-QTOF/MS analysis, reaction mixtures (10 µL) containing Tris-HCl (pH 7.5, 20 mM), AcbJ (5 µM) and V7P (1 mM) or V1,7PP (1 mM) were incubated at 30 °C for 3 h. The substrates and the possible products were analyzed by LC-QTOF/MS using the above-described method A (for V1P, V1,7PP and V7P) or method B (for valienol).

### Kinetic studies of AcbJ toward V1,7PP

To get the kinetic values of AcbJ toward V1,7PP, reaction mixtures (90 µL) containing Tris-HCl (20 mM, pH 7.5), PNP (2 U/mL), MESG (400 µM) and V1,7PP (1500, 1250, 1000, 750, 500, 250, 125, 62.5, or 0 µM) were preincubated at 30 °C and the A_360_ values were monitored until no changes were observed. Subsequently, AcbJ (1.0 µM) and Tris-HCl buffer were added to make 100 µL reaction mixtures. The changes in A_360_ were monitored at 30 °C for 10 min. The data were collected in triplicate. A Lineweaver-Burk plot was used to calculate the *K*_m_ and *k*_cat_ values.

### In vitro reaction of AcbR

To get the initial velocity of AcbR, reaction mixtures (45 µL) containing Tris-HCl (20 mM, pH 7.5), PNP (2 U/mL), inorganic pyrophosphatase (iPP) (2 U/mL), MESG (600 µM), NTP (500 µM) and either V1P, 1-*epi*-V1P, V1,7PP or 1-*epi*-V1,7PP (500 µM) were pre-incubated at 30 °C and the A_360_ was monitored until no changes were observed. Subsequently, AcbR (1.0 µM) and Tris-HCl buffer were added to make 50 µL reaction mixtures and the changes in A_360_ were monitored at 30 °C for 10 min.

For LC-QTOF/MS analysis, a reaction mixture (10 µL) containing Tris-HCl (20 mM, pH 7.5), AcbR (5 µM), 1 mM GTP (1 mM) and V1P (1 mM) (Supplementary Figs. 29–31) was incubated at 30 °C for 3 h. The expected product GDP-V was analyzed by LC-QTOF/MS using the above-described method A.

### Kinetic studies of AcbR

To obtain the kinetic values of AcbR, reaction mixtures (50 µL) containing Tris-HCl (20 mM, pH 7.5), MgCl_2_ (1 mM), GTP (1 mM), PNP (3 U/mL), IPP (2 U/mL), MESG (600 µM) and V1P (1000 µM, 800 µM, 600 µM, 300 µM, 200 µM, 150 µM, 120 µM, 100 µM or 0 µM) were preincubated at 30 °C and the A_360_ was monitored until no changes were observed. Subsequently, AcbR (1.0 µM) and Tris-HCl buffer were added to make 100 µL reaction mixtures and the changes in A_360_ were monitored at 30 °C for 10 min. The data were collected in triplicate. A Lineweaver-Burk plot was used to calculate the *K*_m_ and *k*_cat_ values.

### In vitro reactions of AcbI or AcbS using GDP-V and dTDP4a6dGlc as substrates

To determine whether AcbI or AcbS can catalyze a coupling reaction between GDP-V and dTDP4a6dGlc to furnish dTDP-acarviosin, a reaction mixture (10 µL) containing Tris-HCl (20 mM, pH 7.5), AcbR (5 µM), MgCl_2_ (1 mM), V1P (1 mM), GTP (1 mM), AcbI or AcbS (5 µM), and dTDP4a6dGlc (1 mM) was incubated at 30 °C for 6 h. The reaction mixture was analyzed for the potential product dTDP-acarviosin by LC-QTOF/MS using the above-described method A.

### In vitro reactions of AcbI using dTDP4a6dGlc and oligosaccharides as substrates

To determine whether AcbI can catalyze a coupling between dTDP4a6dGlc and oligosaccharides to yield 4-aminoDGG, reaction mixtures (10 µL each) containing Tris-HCl (20 mM, pH 7.5), AcbI (5 µM), dTDP4a6dGlc (1 mM) and either maltose, maltotriose, maltotetraose or maltopentaose (1 mM) were incubated at 30 °C for 3 h. The reaction mixtures were analyzed for the expected product 4-aminoDGG by LC-QTOF/MS using the above-described method A. Maltotriose and maltopentaose were purified over Sephadex LH-20 to remove contaminating maltose before use.

### In vitro reactions of AcbS or AcbQ using dTDP4a6dGlc and maltose as substrates

To determine whether AcbS and AcbQ can catalyze a coupling reaction between dTDP4a6dGlc and maltose to yield 4-aminoDGG, reaction mixtures (10 µL) containing Tris-HCl (20 mM, pH 7.5), dTDP4a6dGlc (1 mM), maltose (1 mM) and either AcbI, AcbS, or AcbQ (5 µM) were incubated at 30 °C for 3 h. The reaction mixture was analyzed for the potential product 4-aminoDGG by LC-QTOF/MS using the above-described method A.

### Characterization of AcbS as a PsGT

To determine whether AcbS can catalyze a coupling reaction between GDP-V and 4-aminoDGG to produce acarbose, a reaction mixture (10 µL) containing Tris-HCl (20 mM, pH 7.5), AcbR (5 µM), MgCl_2_ (1 mM), V1P (1 mM), GTP (1 mM), AcbS (5 µM), and 4-aminoDGG (1 mM) was incubated at 30 °C for 6 h. The expected product acarbose was analyzed by LC-QTOF/MS using the above-described method A.

### In vitro reactions to demonstrate that only AcbS can catalyze a PsGT reaction

To determine whether AcbI and AcbQ can catalyze a coupling reaction between GDP-V and 4-aminoDGG to produce acarbose, reaction mixtures (10 µL) containing Tris-HCl (20 mM, pH 7.5), AcbR (5 µM), MgCl_2_ (1 mM), V1P (1 mM), GTP (1 mM), 4-aminoDGG (1 mM) and either AcbS, AcbI or AcbQ (5 µM) were incubated at 30 °C for 6 h. The reaction mixtures were analyzed for the expected product acarbose by LC-QTOF/MS using the above-described method A.

### In vitro reconstitution of acarbose formation by AcbI, AcbR, and AcbS

A reaction mixture (10 µL) containing Tris-HCl (20 mM, pH 7.5), AcbR (1 µM), MgCl_2_ (1 mM), V1P (1 mM), GTP (1 mM), AcbS (1 µM), dTDP4a6dGlc (1 mM), AcbI (1 µM) and maltose (1 mM) was incubated at 30 °C for 6 h. In parallel, a reaction mixture (8 µL) containing Tris-HCl (20 mM, pH 7.5), AcbR (1 µM), MgCl_2_ (1 mM), V1P (1 mM), GTP (1 mM), AcbS (1 µM), and dTDP4a6dGlc (1 mM) was incubated at 30 °C for 3 h. The enzymes were removed by ultrafiltration using a Pierce™ protein concentrator (MWCO: 3 K) at 4 °C, 13000 x g, 10 min. Subsequently, AcbI (1 µM) and maltose (1 mM) were added to the reaction mixture and the reaction was incubated at 30 °C for another 3 h. A reaction mixture without AcbI was used as a negative control. The reaction mixtures were analyzed for the expected product acarbose by LC-QTOF/MS using the above-described method A.

### In vitro reactions of AcbS using alternative substrates

To determine whether AcbS can accept maltotriose (instead of 4-aminoDGG) as substrate, a reaction mixture (10 µL) containing Tris-HCl (20 mM, pH 7.5), AcbR (5 µM), MgCl_2_ (1 mM), V1P (1 mM), GTP (1 mM), maltotriose (1 mM) and AcbS (5 µM) was incubated at 30 °C for 6 h. The reaction mixture was analyzed for the potential product V-GGG by LC-QTOF/MS using the above-described method A.

To determine whether AcbS can accept GDP-glucose (instead of GDP-V) as substrate, a reaction mixture (10 µL) containing Tris-HCl (20 mM, pH 7.5), GDP-glucose (1 mM), 4-aminoDGG (1 mM) and AcbS (5 µM) was incubated at 30 °C for 6 h. The reaction mixture was analyzed for the potential product G-N-DGG by LC-QTOF/MS using the above-described method A.

### Construction of the phylogenetic tree

The homologues of AcbS and AcbI were identified using a BlastP search (BLAST + version 2.12.0). Standard parameters were used in the search, except that the maximum number of target sequences was changed from 100 to 500. For phylogenetic tree construction, 242 amino acid sequences, which show high identity to both AcbI and AcbS, were used. The phylogenetic tree was constructed by Geneious Tree Builder program using Jukes-Cantor genetic distance model and Neighbor-Joining tree building method. The constructed tree was edited by Geneious Prime (version 2019.1.3).

### Reporting summary

Further information on research design is available in the Nature Research Reporting Summary linked to this article.

## Supplementary information

Supplementary Information

Reporting Summary

## Data Availability

All additional experimental data generated in this study are provided in the Supplementary information (Supplementary Tables 5 – 8; Supplementary Figs. 32 – 45). DNA and amino acid sequences for all proteins used in this study are available in the NCBI database; their accession numbers can be found in Supplementary Table 1. Accession numbers for phylogenetic tree analysis are given in the source data file. The bacterial strains and plasmids can be sourced from the corresponding author. Source data are provided with this paper.

## Source data

Source Data
