# Supplementary material for: Complete biosynthetic pathway to the antidiabetic drug acarbose
Source: Nat Commun. 2022 Jun 15;13:3455. doi: 10.1038/s41467-022-31232-4 (PMC9200736; doi:10.1038/s41467-022-31232-4)
Supplement: Supplementary file 1 — Supplementary Information [file 41467_2022_31232_MOESM1_ESM.pdf]

SUPPLEMENTARY INFORMATION

Complete biosynthetic pathway to the antidiabetic drug acarbose

Takeshi Tsunoda, Arash Samadi, Sachin Burade, and Taifo Mahmud*

Department of Pharmaceutical Sciences, Oregon State University, Corvallis, OR 97331-3507 (USA)

Supplementary Tables

Supplementary Table 1. Acarbose biosynthetic genes in *Actinoplanes* sp. SE50/110

Gene	Putative function of the gene product	Accession number
acbZ	pullulanase-type α-1,6-glucosidase	RZU54349.1
acbY	ABC-2 family transporter protein	WP_210924000.1
acbX	ABC-2 family transporter protein	WP_210923999.1
acbW	ATP-binding cassette domain-containing protein	WP_210923998.1
acbV	aminotransferase class III-fold PLP-dependent enzyme	WP_212826328.1
acbU	hypothetical protein	WP_212826336.1
acbS	glycogen/starch synthase	WP_212826338.1
acbR	NTP transferase domain-containing protein	WP_212826340.1
acbP	8-oxo-dGTP pyrophosphatase	WP_244215755
acbI	glycogen/starch synthase	WP_212826345.1
acbJ	Cof-type HAD-IIB family hydrolase	WP_212826347.1
acbQ	4-α-glucanotransferase	WP_212826349.1
acbK	adenosine kinase	WP_184538174.1
acbM	alcohol dehydrogenase catalytic domain-containing protein	WP_126388024.1
acbL	ROK family protein	WP_126388022.1
acbN	SDR family NAD(P)-dependent oxidoreductase	WP_193207917.1
acbO	TIM barrel protein	WP_211769137.1
acbC	sedoheptulose 7-phosphate cyclase	WP_052482047.1
acbB	dTDP-glucose 4,6-dehydratase	WP_078976026.1
acbA	glucose-1-phosphate thymidylyltransferase	WP_225258639.1
acbE	pullulanase-type α-1,6-glucosidase	WP_212990052.1
acbD	α-amylase	NUR52166.1

Supplementary Table 2. Bacterial strains used in this study

Strains	Relevant genotype/comments	Source/Ref
Escherichia coli DH10B	*F⁻ mcrA Δ(mrr-hsdRMS-mcrBC)φ80lacZΔM15 ΔlacX74 recA1 endA1 araD139 Δ(ara, leu)7697 galU galK λ⁻ rspL nupG.*	GibcoBRL
Escherichia coli BL21(DE3)pLysS	*F⁻ ompT* $hsdS_B$ (r_B^- m_B^-) *gal dcm* (DE3) pLysS (CmR).	Promega
Escherichia coli ET12567(pUZ8002)	CmR, TetR, dam-13::Tn9, dcm-6, pUZ8002 (pRP4 derivative, oriT, KanR).	[1]
Streptomyces lividans TK24	*S. lividans* wild type.	[2]
Streptomyces lividans/pXY201-acbJ	*S. lividans* TK24 plus pXY201-*acbJ.*	This study
Streptomyces lividans/pXY201-acbR	*S. lividans* TK24 plus pXY201-*acbR.*	This study

Supplementary Table 3. Primers used in this study.

Primer	Sequence (5´→3´)
*acbR*_F	AAAACATATGAGCACGGGCGTACGGGC
*acbR*_R	AAGAATTCTTAAGCTTTCGCTCCGGTGGTGAC
*acbJ*_F	AAAACATATGGACGGCATCGGCACCACA
*acbJ*_R	AAGAATTCTCATCGCAGGCTTGCCTTTCGG
*acbS*_F	AAAGATCTATGCACATCATCGAGACGTACTTC
*acbS*_R	AAGAATTCTCATGCCGTCACCTCGTCC
*acbK*_F	AAAGATCTATGTCGGAGCACACCGACGT
*acbK*_R	AAGAATTCGGTGCGGTGGCCGCTTCA
*acbI*_F	AAAGATCTATGCACGTGGTGAGCTTCGC
*acbI*_R	AAGAATTCGCCGATGCCGTCCATGTCA
*acbQ*_F	CCGAGCTCGAGATCTATGACCACCACGACGGATGCC
*acbQ*_R	TCAAGCTTCGAATTCTCAGCGAGGTCAGGGTGTGGT
*acbU*_F	AAAACATATGACACCCCGGCCGGTC
*acbU*_R	AAGAATTCTCAGCTCTCCCCGGTGACC

Supplementary Table 4. Plasmids used in this study.

Plasmid	Description	Source/Ref
pBlueScript II SK(–)	ColE1-based phagemid vector with f1 (–) and pUC origins; T3, T7 and lac promoters; *bla*.	Stratagene
pRSET B	N-terminal his-tagged fusion peptide.	Novagen
pET-28b(+)	N- and C-terminal his-tagged fusion peptide.	Novagen
pXY201	Multicopy *Streptomyces* expression vector, thiostrepton promoter, N-terminal His tag, Amp^R, Apr^R, ColE1, oriT.	[3]
pRSET B-*acbU*	pRSETB containing *acbU* gene.	This study
pRSET B-*acbS*	pRSETB containing *acbS* gene.	This study
pRSET B-*acbI*	pRSETB containing *acbI* gene.	This study
pRSET B-*acbK*	pRSETB containing *acbK* gene.	This study
pRSET B-*valC*	pRSETB containing *valC* gene from *Streptomyces hygroscopicus* subsp. *jinggangensis* 5008.	[4]
pXY201-*acbJ*	pXY201 containing *acbJ* gene.	This study
pXY201-*acbR*	pXY201 containing *acbR* gene.	This study
pET28b(+)-*acbQ*	pET28b(+) containing *acbQ* gene.	This study

Supplementary Table 5. Combined Kinase/ATPase activities of AcbU with various substrates*

Substrate	ATP added	Initial velocity (nM/min)**
V7P	YES	216 ± 27
V	YES	141 ± 13
V1P	YES	119 ± 7
No substrate	YES	118 ± 8

*Reaction without AcbU was used as a blank.
** n = 3 analytical replicates.

Supplementary Table 6. Kinase activity of AcbU with various substrates*

Substrate	ATP added	Initial velocity (nM/min)**
V7P	YES	98 ± 19
V	YES	23 ± 7
V1P	YES	1 ± 3

*Reaction without substrate but with ATP was used as a blank.
** n = 3 analytical replicates.

Supplementary Table 7. Phosphatase activity of AcbJ with various substrates*

Substrate	Initial velocity (μM/min)**
V1,7PP	5.1 ± 1.6
1-epi-V1,7PP	0.7 ± 0.4
V7P	6.1 ± 1.1
Aca7P	1 ± 0.3

* Reaction without AcbJ was used as a blank.
** n = 3 analytical replicates.

Supplementary Table 8. Nucleotidyltransferase activity of AcbR with various substrates and NTPs*

Substrate	NTP	Initial velocity (μM/min)**
V1P	ATP	0.7 ± 0.8
	UTP	2.9 ± 1.9
	GTP	12.5 ± 1.1
	CTP	0.3 ± 1.4
	dTTP	0.9 ± 1.2
1-epi-V1P	ATP	3.1 ± 2.9
	UTP	1.3 ± 1.6
	GTP	1.2 ± 1.2
	CTP	2.2 ± 1.5
	dTTP	5.1 ± 1.6
V1,7PP	ATP	3.2 ± 2.8
	UTP	3.2 ± 1.9
	GTP	0.3 ± 1.4
	CTP	0.4 ± 2
	dTTP	3.5 ± 2.4
1-epi-V1,7PP	ATP	0.8 ± 1.4
	UTP	2.4 ± 1.7
	GTP	0.7 ± 2.8
	CTP	0.8 ± 1.1
	dTTP	2.1 ± 2.8

* Reaction without AcbR was used as a blank.
** n = 3 analytical replicates.

Supplementary Figures

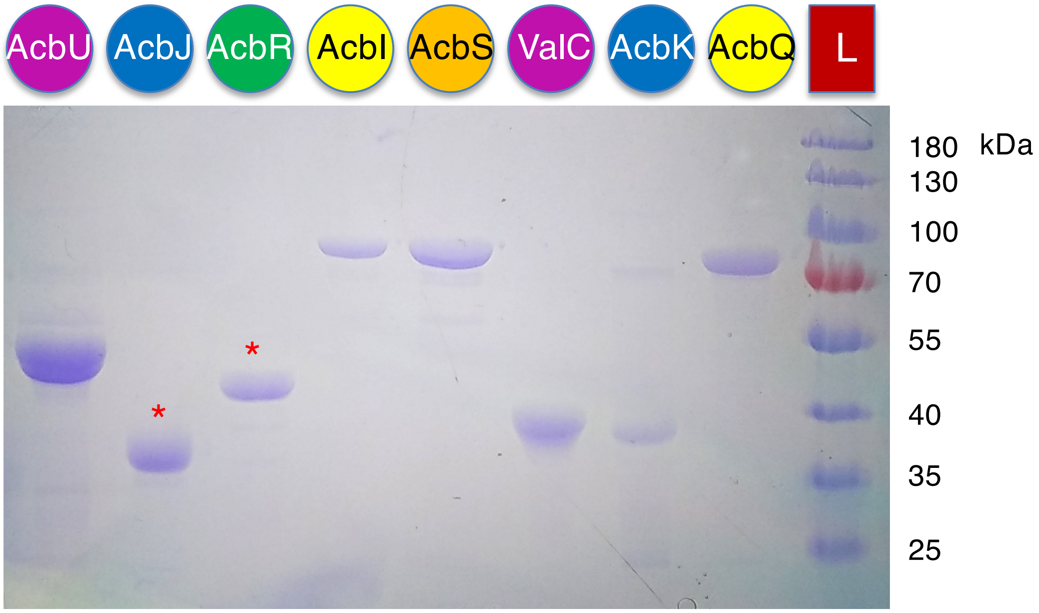

Supplementary Figure 1. SDS-PAGE of purified recombinant proteins used in this study. Red star, recombinant proteins produced in *Streptomyces lividans* TK24; L, protein ladder. All experiments were carried out at least three times with similar results.

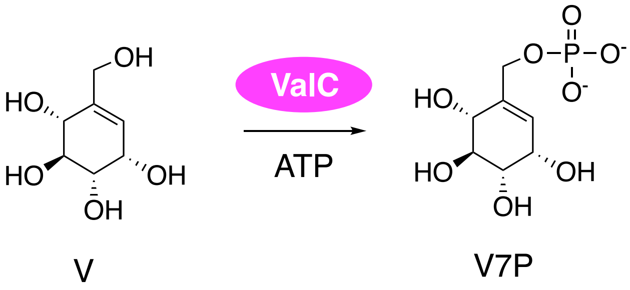

Supplementary Figure 2. Bioconversion of V to V7P by ValC.

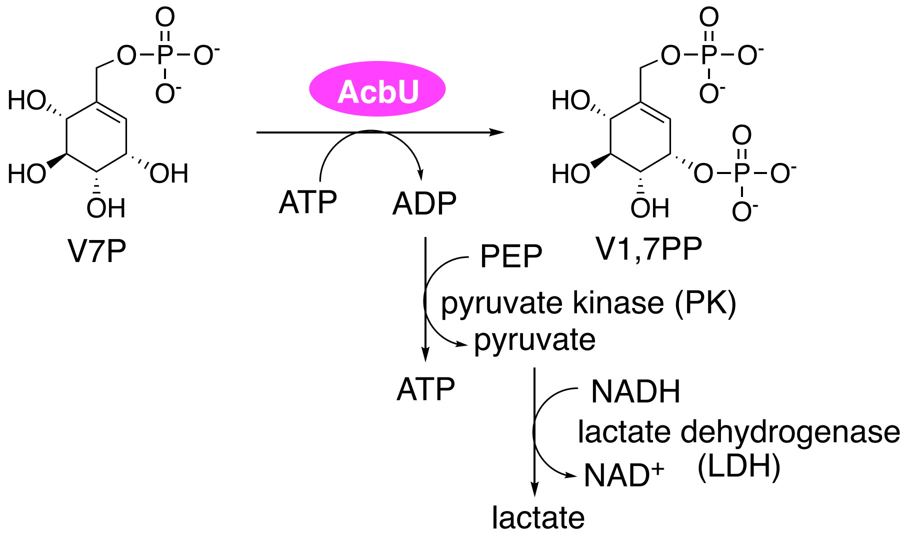

Supplementary Figure 3. PK/LDH coupled enzyme assay for AcbU kinase activity.

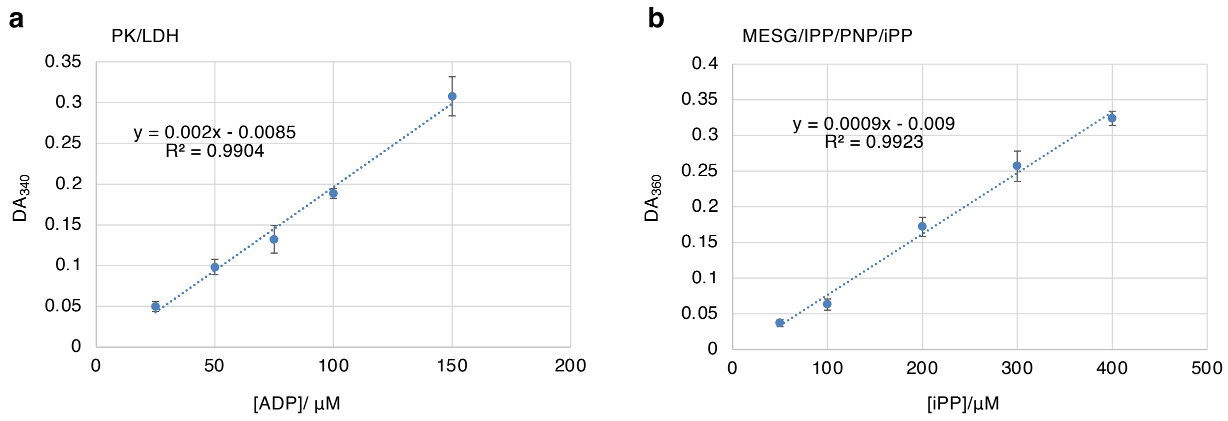

Supplementary Figure 4. Calibration curves for ADP and iPP. (**a**) Calibration curve for ADP using the PK/LDH assay; and (**b**) calibration curve for iPP using the MESG/IPP/PNP assay. Error bars indicate mean ± standard deviation (n = 3 analytical replicates).

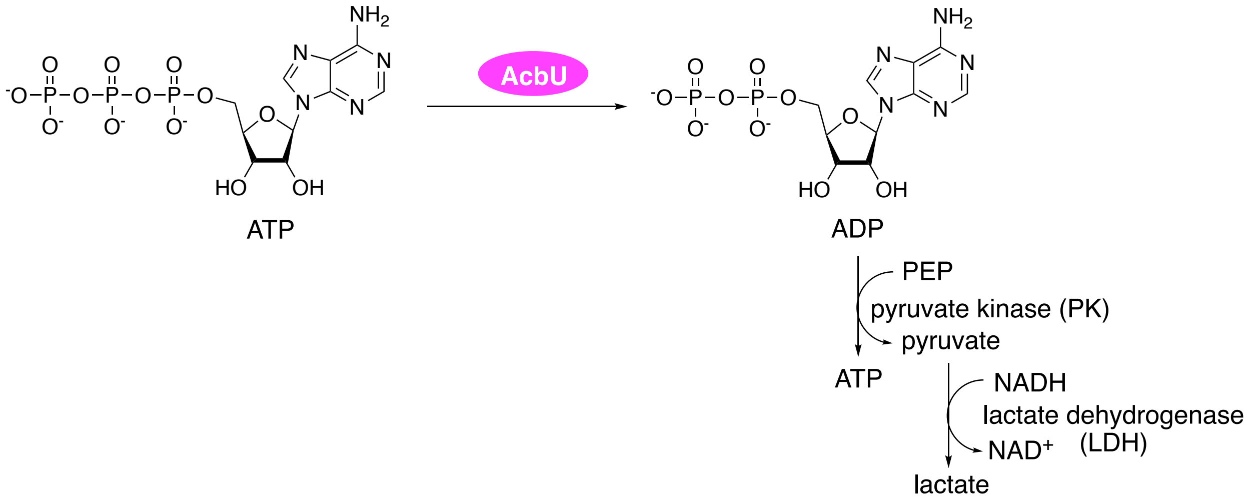

Supplementary Figure 5. PK/LDH coupled enzyme assay for AcbU ATPase activity.

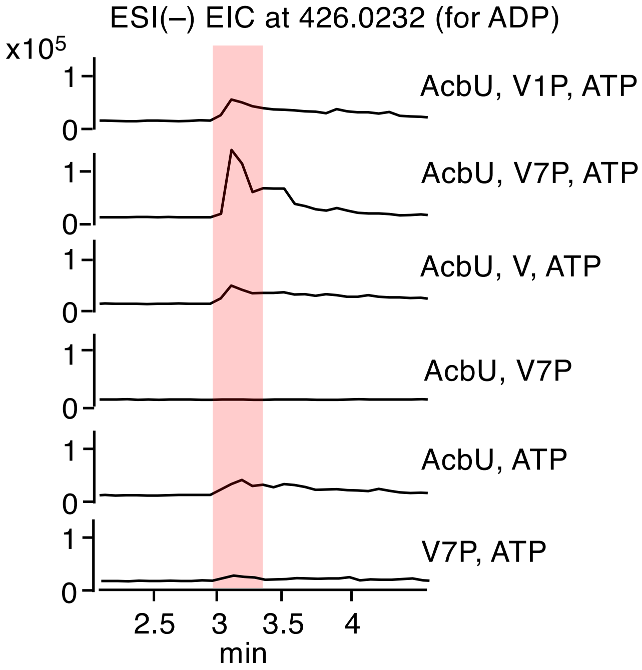

Supplementary Figure 6. LC-MS analysis of AcbU reactions with and without substrate and/or ATP. All experiments were carried out at least three times with similar results.

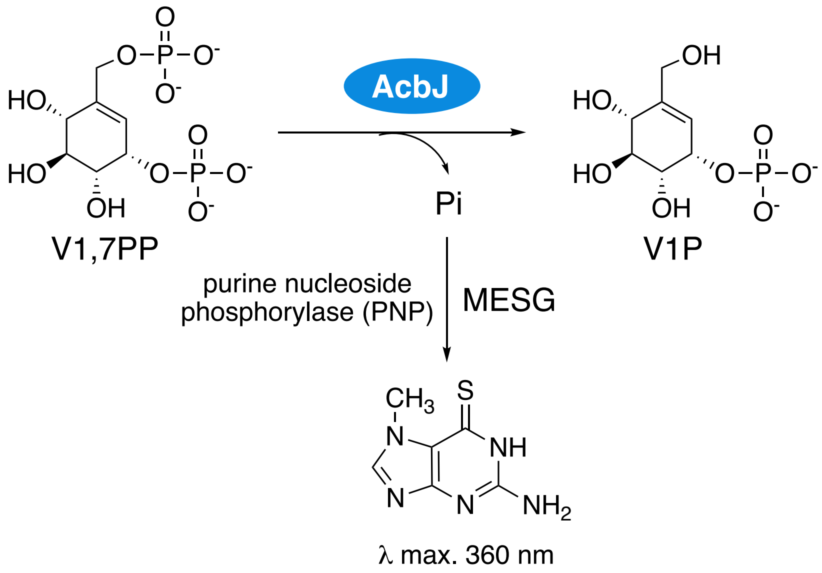

Supplementary Figure 7. Measurement of AcbJ dephosphorylase activity by purine nucleoside phosphorylase assay.

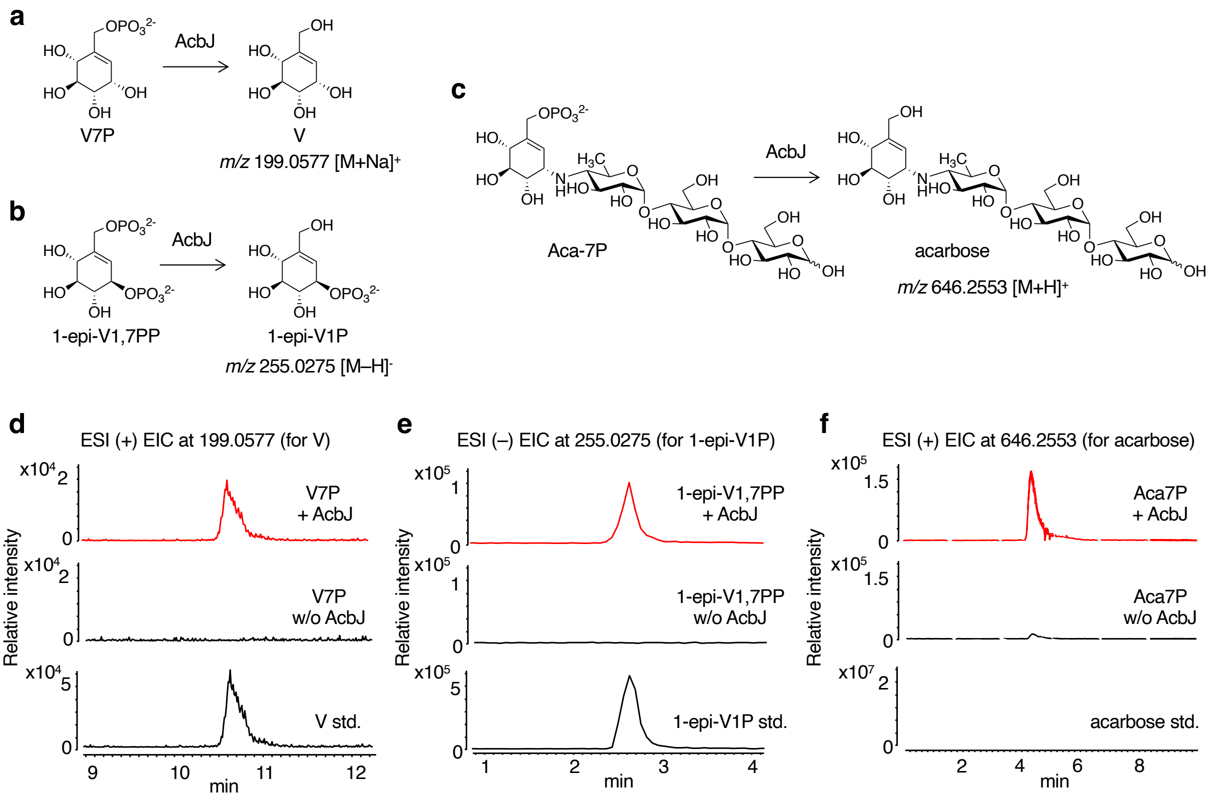

Supplementary Figure 8. ESI MS analysis of AcbJ phosphatase activity with various substrates. (**a**) reaction scheme of V7P with AcbJ; (**b**) reaction scheme of 1-epi-V1,7PP with AcbJ; (**c**) reaction scheme of Aca7P with AcbJ; (**d**) ESI (+) EIC for valienol (V) at *m/z* 199.0577 of reaction mixtures containing V7P with or without AcbJ; (**e**) ESI (–) EIC for 1-epi-V1P at *m/z* 255.0275 of reaction mixtures containing 1-epi-V1,7PP with or without AcbJ; and (**f**) ESI (+) EIC for acarbose at *m/z* 646.2553 of reaction mixtures containing Aca7P with or without AcbJ. All experiments were carried out at least three times with similar results.

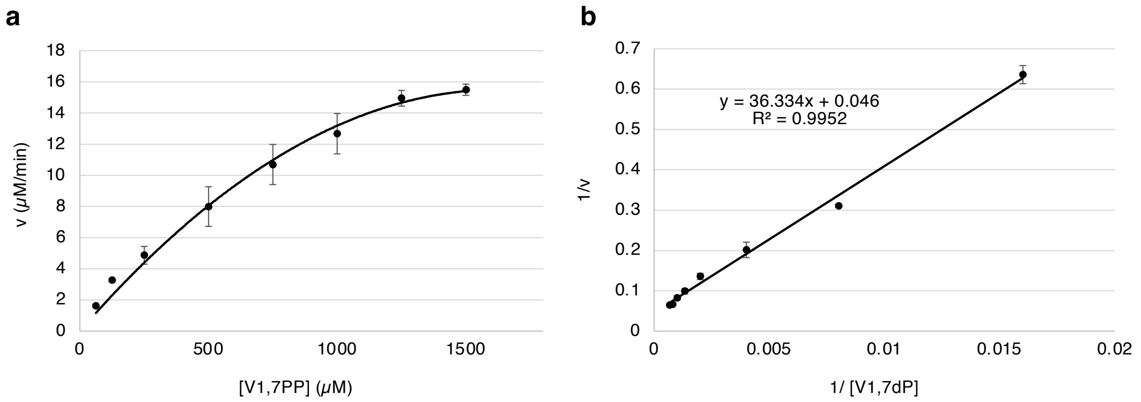

Supplementary Figure 9. Steady-state kinetic studies of recombinant AcbJ. (**a**) Michaelis-Menten plot; (**b**) Lineweaver-Burk plot. K_m = 817 ± 168 μM, k_{cat} = 22 ± 4 min^{-1}. Error bars indicate mean ± standard deviation (n = 3 analytical replicates).

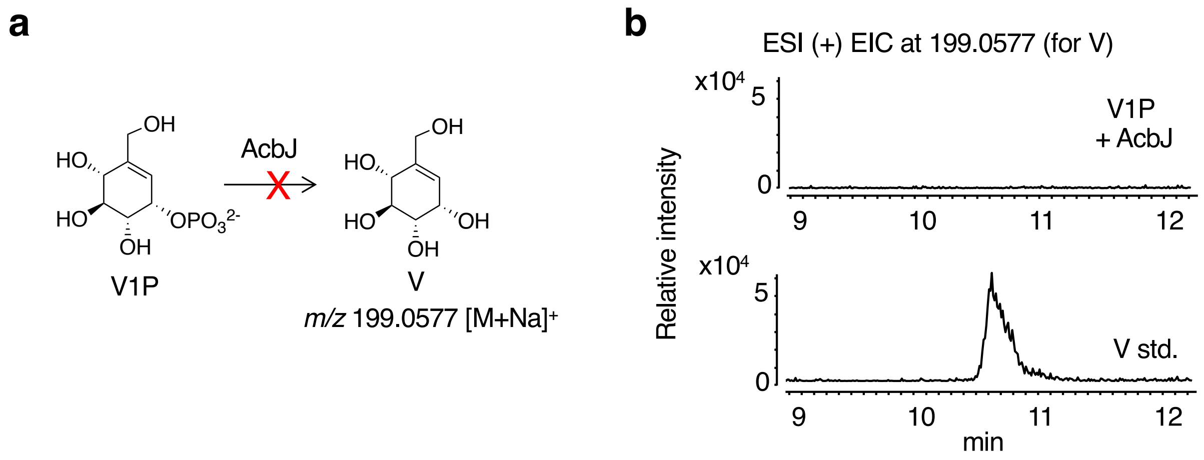

Supplementary Figure 10. ESI MS analysis of AcbJ phosphatase activity with valienol 1-phosphate (V1P). (**a**) reaction scheme of V1P with AcbJ; (**b**) ESI (+) EIC for valienol (V) at *m/z* 199.0577 of a reaction mixture containing V1P with AcbJ and a solution containing authentic V. All experiments were carried out at least three times with similar results.

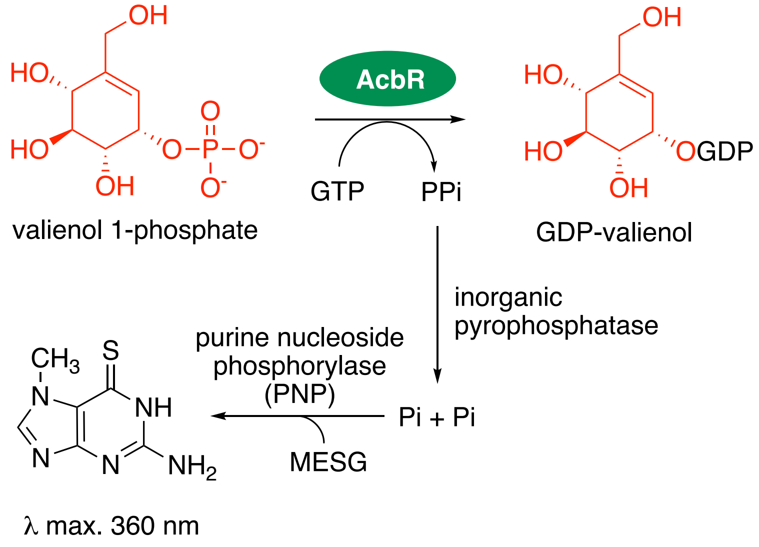

Supplementary Figure 11. Characterization of AcbR by enzymatic coupling assay using inorganic pyrophosphatase (IPP), nucleotidyltransferase (PNP) and MESG.

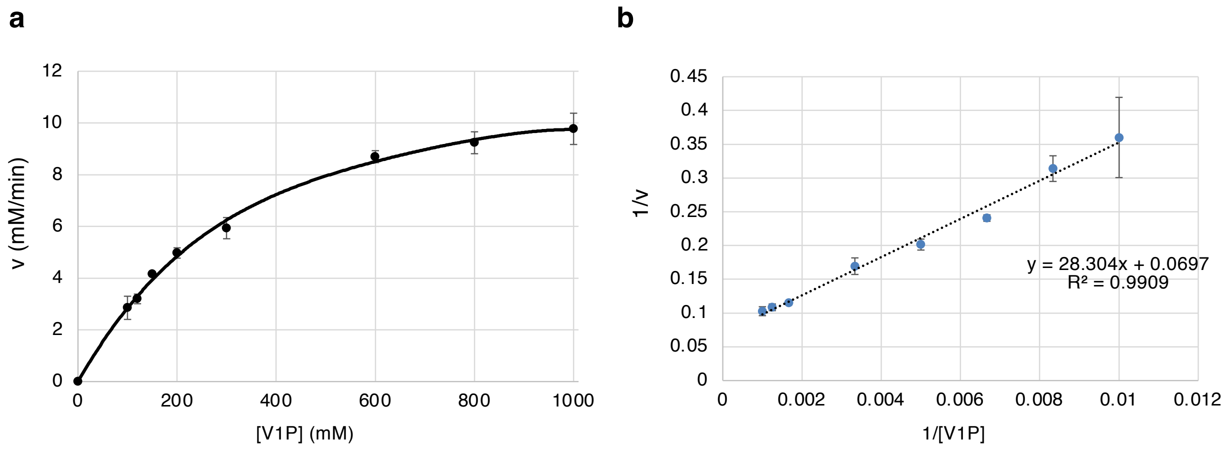

Supplementary Figure 12. Steady-state kinetic studies of recombinant AcbR. (**a**) Michaelis-Menten plot; (**b**) Lineweaver-Burk plot. K_m = 412 ± 83 μM, k_{cat} = 14 ± 1 min^{-1}. Error bars indicate mean ± standard deviation (n = 3 analytical replicates).

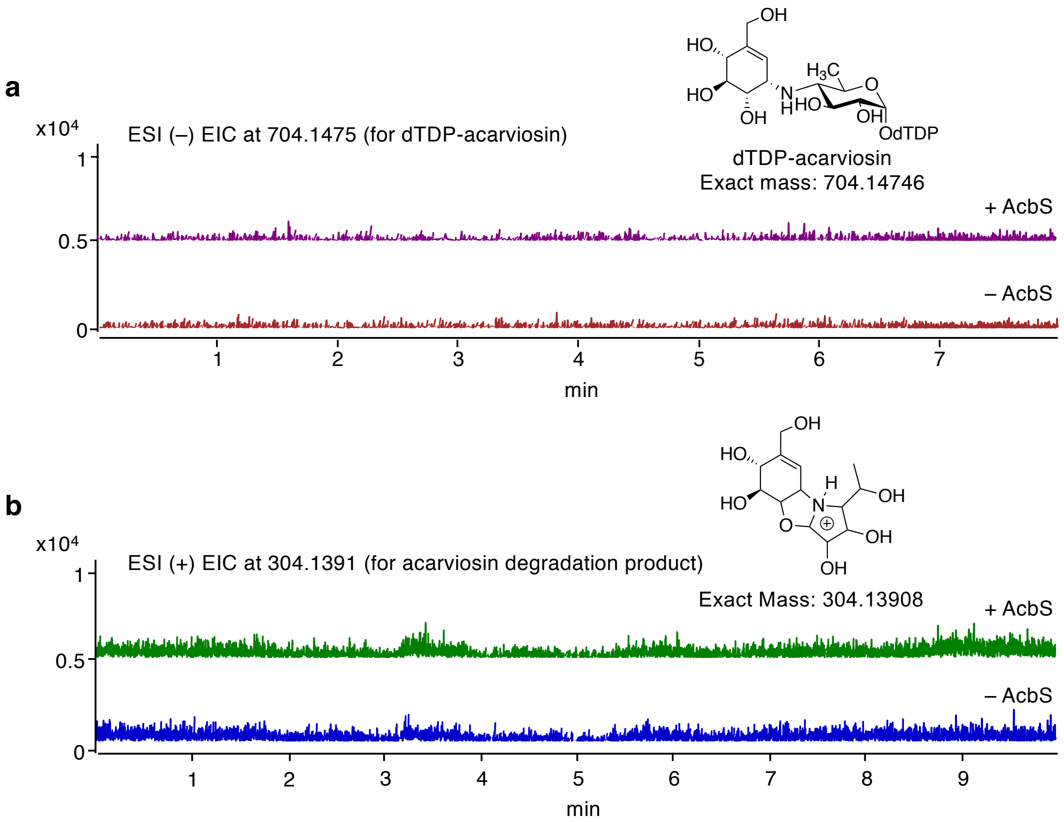

Supplementary Figure 13. Mass spectrometry analysis of AcbS reactions with GDP-V and synthetically prepared dTDP4a6dGlc. (**a**) ESI (–) EIC (*m/z* 704.1475) for dTDP-acarviosin; (**b**) ESI (–) EIC (*m/z* 304.1391) for acarviosin degradation product. All experiments were carried out at least three times with similar results.

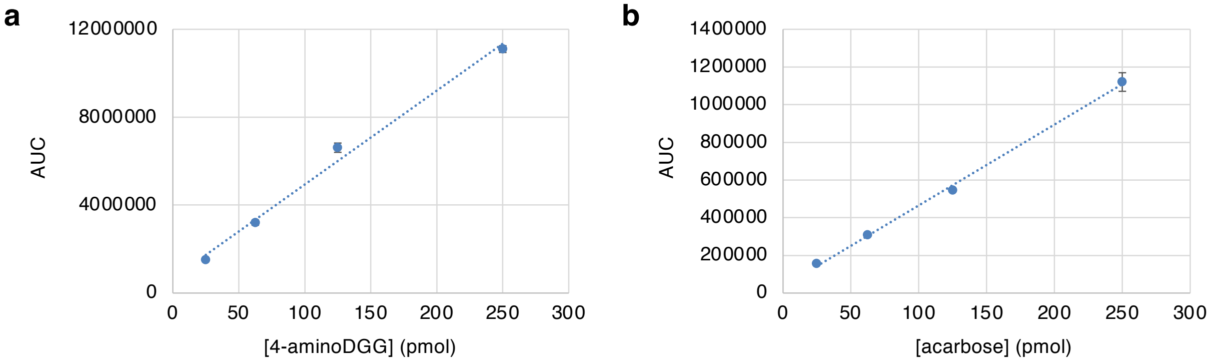

Supplementary Figure 14. Calibration curves for 4-aminoDGG and acarbose based on the areas under curve (AUC) of their LC-MS chromatograms. (**a**) Calibration curve for 4-aminoDGG and (**b**) calibration curve for acarbose. Error bars indicate mean ± standard deviation (n = 3 analytical replicates).

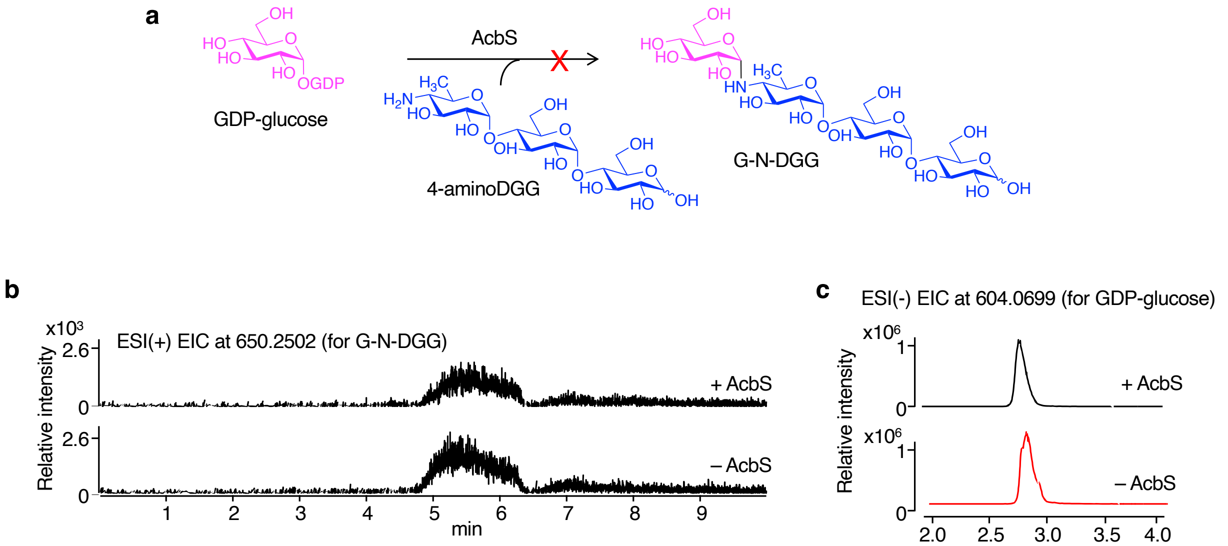

Supplementary Figure 15. AcbS does not catalyze the coupling between GDP-glucose and 4-aminoDGG. (**a**) reaction scheme of GDP-glucose and 4-aminoDGG, (**b**) ESI(+) EIC for G-N-DGG at *m/z* 650.2502 of reaction mixtures containing GDP-glucose and 4-aminoDGG with and without AcbS, and (**c**) ESI EIC (–) for DGP-glucose at *m/z* 604.0699 of reaction mixtures containing GDP-glucose and 4-aminoDGG with or without AcbS. No noticeable consumption of GDP-glucose was observed. All experiments were carried out at least three times with similar results.

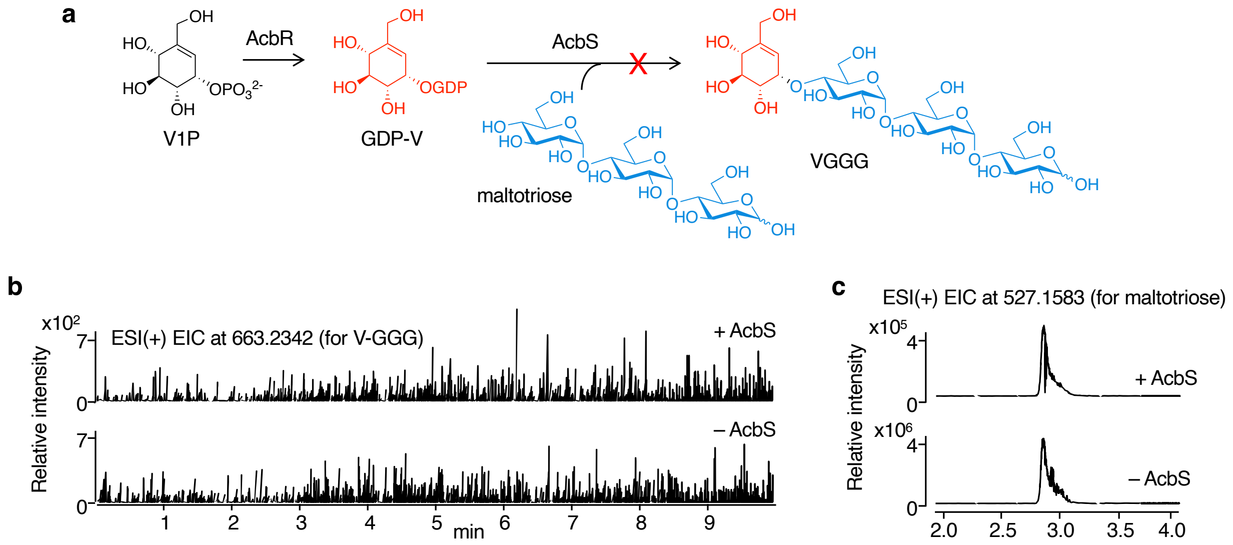

Supplementary Figure 16. AcbS does not catalyze the coupling between GDP-V and maltotriose. (**a**) reaction scheme of GDP-V and maltotriose, (**b**) ESI(+) EIC for VGGG at *m/z* 663.2342 of reaction mixtures containing GDP-V and maltotriose with or without AcbS, and (**c**) EIC for maltotriose at *m/z* 527.1583 of reaction mixtures containing GDP-V and maltotriose with or without AcbS. No noticeable consumption of maltotriose was observed. All experiments were carried out at least three times with similar results.

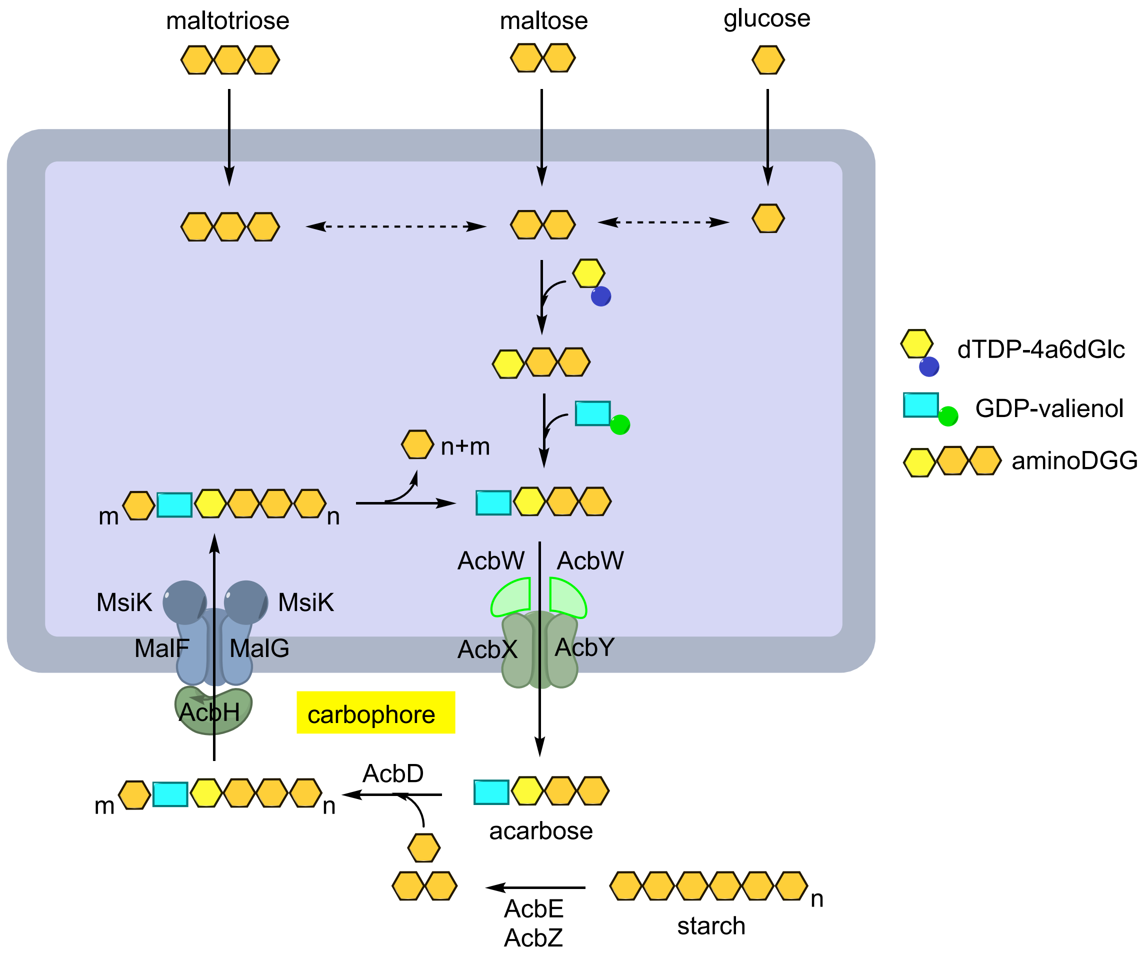

Supplementary Figure 17. Proposed function of acarbose as a carbophore.[5,6]

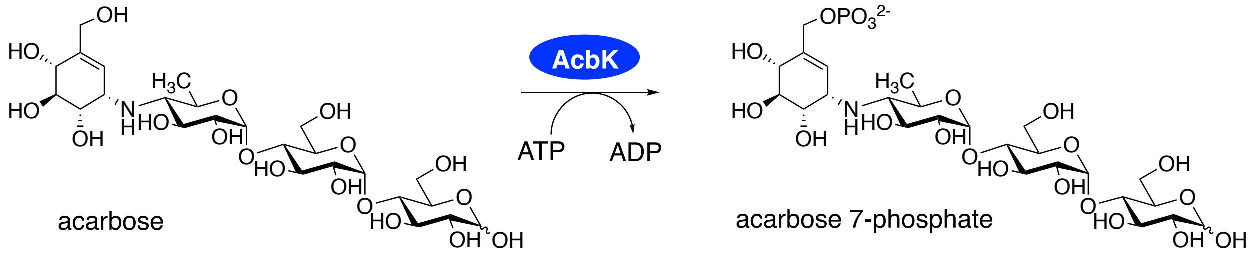

Supplementary Figure 18. Preparation of acarbose 7-phosphate using the acarbose kinase AcbK.

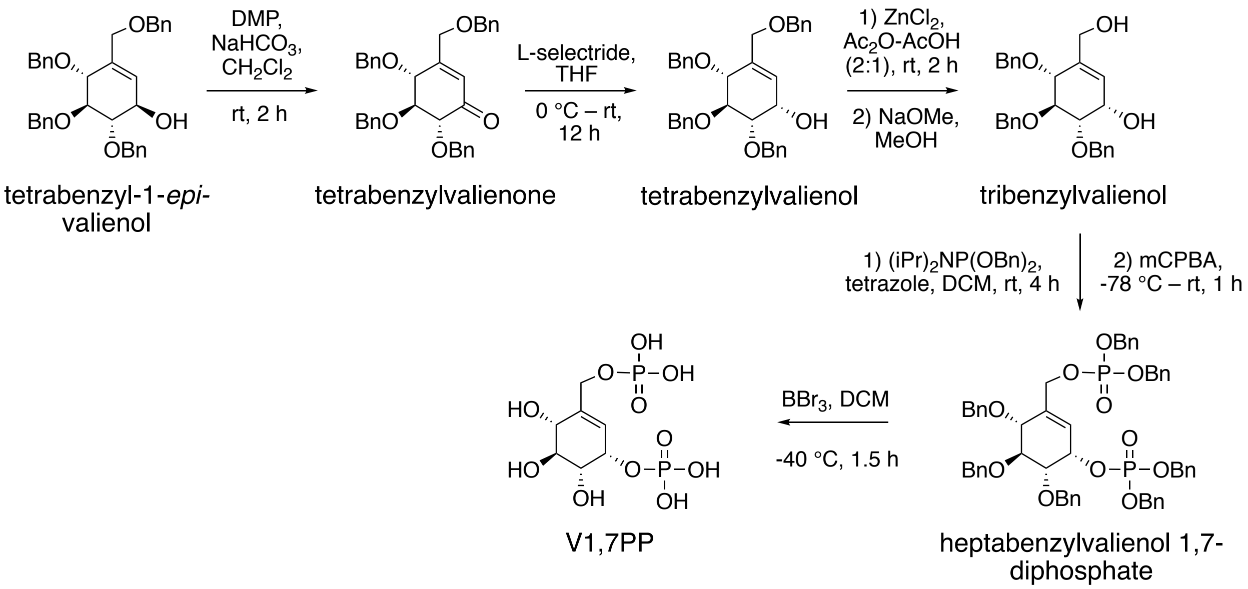

Supplementary Figure 19. Chemical synthesis of valienol 1,7-diphosphate (V1,7PP) from tetrabenzyl-1-*epi*-valienol.

Synthesis of valienol 1,7-diphosphate

Synthesis of tetrabenzylvalienone. Tetrabenzyl-1-epi-valienol (400 mg, 0.745 mmol) was dissolved in CH_2Cl_2 (2 mL) under Argon atmosphere, then $NaHCO_3$ (2 eq.) followed by DMP (379 mg, 0.894 mmol) were added and the reaction was stirred for 2 h at room temperature under Argon. After completion, the reaction mixture was quenched with saturated solution of $Na_2S_2O_3$ (0.5 mL). The solvent was removed using a rotary evaporator, and the residue was re-dissolved in EtOAc (4 mL). The organic layer was washed with 10% aqueous $Na_2S_2O_3$ (5 mL), saturated aqueous $NaHCO_3$ (5 mL), and brine (5 mL). The organic layer was dried over Na_2SO_4, filtered, and concentrated under reduced pressure to get crude compound. The crude was purified using column chromatography (silica gel, *n*-hexane–EtOAc = 9:1) to afford tetrabenzylvalienone (334 mg, 83% yield) as colorless syrup. Analytical data matched reported data.[7]

Tetrabenzylvalienone: ^{1}H NMR (700 MHz, $CDCl_3$): δ 7.47 – 7.26 (m, 20H), 6.25 (s, 1H), 5.14 (d, J = 11.4 Hz, 1H), 5.04 (d, J = 10.8 Hz, 1H), 4.94 (d, J = 10.9 Hz, 1H), 4.78 (dd, J = 11.1, 5.9 Hz, 2H), 4.72 (d, J = 11.0 Hz, 1H), 4.54 (d, J = 3.8 Hz, 2H), 4.41 (d, J = 8.0 Hz, 1H), 4.30 (d, J = 16.1 Hz, 1H), 4.11 (dd, J = 13.3, 9.9 Hz, 2H), 4.07 – 4.03 (m, 1H).

Synthesis of tetrabenzylvalienol. Tetrabenzylvalienone (320 mg, 0.598 mmol) was dissolved in dry THF under Argon atmosphere and L-selectride 1M sol. in THF (1.2 mL, 1.2 mmol) was added dropwise at 0 °C. Subsequently, reaction was stirred at 0 °C for 1 h and then at rt for 12 h. The reaction was quenched by an NH_4Cl solution. The product was extracted using EtOAc. The organic layer was dried over Na_2SO_4, filtered, and concentrated under reduced pressure to get crude compound. The crude was purified using column chromatography (silica gel, *n*-hexane–EtOAc = 8:2) to afford tetrabezylvalienol (223 mg, 69% yield) as colorless syrup. Analytical data matched reported data.[8]

Tetrabenzylvalienol: ^{1}H NMR (500 MHz, $CDCl_3$): δ 7.37 – 7.24 (m, 20H), 5.93 (dd, J = 4.9, 1.2 Hz, 1H), 4.90 (d, J = 11.1 Hz, 1H), 4.85 – 4.75 (m, 3H), 4.69 (dd, J = 13.1, 11.5 Hz, 2H), 4.49 (dd, J = 25.9, 11.8 Hz, 2H), 4.33 (t, J = 4.5 Hz, 1H), 4.26 (d, J = 12.2 Hz, 1H), 4.18 (d, J = 6.7 Hz, 1H), 4.08 (dd, J = 9.3, 6.8 Hz, 1H), 3.97 (d, J = 12.3 Hz, 1H), 3.63 (dd, J = 9.3, 4.1 Hz, 1H).

Synthesis of tribenzylvalienol. $ZnCl_2$ (533 mg, 3.91 mmol) was added to a solution of tetrabenzyl valienol (210 mg, 0.391 mmol) in Ac_2O–AcOH (2:1, 4 mL), and the reaction mixture was stirred for 2 h at room temperature. The reaction mixture was diluted with water (10 mL) and EtOAc (10 mL), and the organic layer was washed with water (10 mL) and a saturated aqueous Na_2CO_3 solution (3 X 10 mL). The organic layer was dried over Na_2SO_4, filtered, and concentrated under reduced vacuum to give a crude sample. The crude sample was dissolved in MeOH (2.5 mL) and NaOMe (1 mL, 30% solution in MeOH) was added to the reaction mixture. The reaction mixture was stirred for 4 h at room temperature and quenched with water (4 mL) and saturated aqueous NH_4Cl (10 mL). The reaction mixture was diluted with EtOAc (10 mL), and the organic layer was further washed with water (5 mL) and brine (5 mL). The organic layer was dried over Na_2SO_4, filtered, and concentrated under reduced vacuum. The product was purified using column chromatography (silica gel, *n*-nexane–EtOAc = 1:2) to yield the title compound (123 mg, 70% yield) as white solid. Analytical data matched reported data.[8]
Tribenzylvalienol: ^1H NMR (500 MHz, $CDCl_3$): δ 7.38 – 7.32 (m, 15H), 5.89 – 5.85 (m, 1H), 5.69 (bs, 1H), 5.32 (bs, 1H), 5.02 – 4.67 (m, 6H), 4.33 (dd, J = 12.3, 7.7 Hz, 1H), 4.19 – 4.07 (m, 4H), 3.62 (dd, J = 9.2, 4.1 Hz, 1H).

Synthesis of heptabenzylvalienol 1,7-diphosphate. A solution tribenzylvalienol (110 mg, 0. 246 mmol) in CH_2Cl_2 (4 mL) was added to a solution of dibenzyl *N*,*N*-diisopropylphosphoramidite (306 mg, 0.0.886 mmol) and 1*H*-tetrazole (69 mg, 0.985 mmol) in CH_2Cl_2 (4 mL), which had been stirred for 40 min at room temperature under Argon, and the reaction mixture was further stirred for 4 h at room temperature. The reaction mixture was cooled to –78 °C and *m*-CPBA (191 mg, 1.11 mmol) was added. After the cooling bath was removed, stirring of the reaction mixture was continued for an additional 40 min at room temperature. The solvent was removed using a rotary evaporator, and the residue was re-dissolved in EtOAc (15 mL). The organic layer was washed with 10% aqueous Na_2SO_3 (10 mL), saturated aqueous $NaHCO_3$ (10 mL), and brine (10 mL). The organic layer was dried over Na_2SO_4, filtered, and concentrated under reduced vacuum to get crude compound. Column chromatography (silica gel, *n*-hexane–EtOAc = 1:1) yielded the title compound (137 mg, 57% yield) as colorless syrup. Analytical data matched reported data.[8]
Heptabenzylvalienol 1,7-diphosphate: ^1H NMR (500 MHz, $CDCl_3$): δ 7.44 – 7.18 (m, 35H), 5.75 (d, J = 4.7 Hz, 1H), 5.15 – 4.96 (m, 9H), 4.93 (d, J = 11.1 Hz, 1H), 4.81 (dd, J = 24.4, 11.1 Hz, 2H), 4.61 (ddd, J = 18.9, 17.0, 10.0 Hz, 4H), 4.43 (d, J = 6.0 Hz, 1H), 4.09 – 4.01 (m, 2H), 3.54 – 3.48 (m, 1H); ^{31}P NMR (202 MHz, $CDCl_3$): δ -0.87 (s), -1.31 (s).

Synthesis of valienol 1,7-diphosphate. BBr_3 (276 µL of 1.0 M solution in CH_2Cl_2, 0.276 mmol) was added to a solution of heptabenzyl-valienol 1,7-diphosphate (30 mg, 0.031 mmol) in CH_2Cl_2, and the reaction mixture was stirred for 90 min at -40 °C. Water (1.0 mL) was added to the reaction mixture at the same temperature, and the pH of the solution was adjusted to weak basic ($_{P}H$ = ~8) using saturated aqueous $NaHCO_3$ solution. The reaction mixture was diluted with H_2O (3 mL) and CH_2Cl_2 (3 mL), and the aqueous layer was further extracted with CH_2Cl_2 (5 mL). The aqueous layer was subjected to biogel filtration using deionized water as eluent. The product fractions were pooled and lyophilized. The resulting white solid was dissolved in H_2O (0.2 mL) and desalted by passing it through a Sephadex LH-20 column using H_2O as an eluent. The product fractions were combined and lyophilized to give valienol 1,7-diphosphate (6 mg, 58 % yield). Analytical data matched reported data.[8]
Valienol 1,7-diphosphate: ^1H NMR (500 MHz, D_2O): δ 5.88 (d, J = 5.6 Hz, 1H), 4.66 – 4.57 (m, 1H), 4.46 (dd, J = 12.7, 8.3 Hz, 1H), 4.10 (dd, J = 11.6, 6.8 Hz, 2H), 3.76 (dd, J = 10.6, 8.0 Hz, 1H), 3.53 (dd, J = 10.6, 4.0 Hz, 1H); ^{31}P NMR (202 MHz, D_2O): δ 4.54 (s).

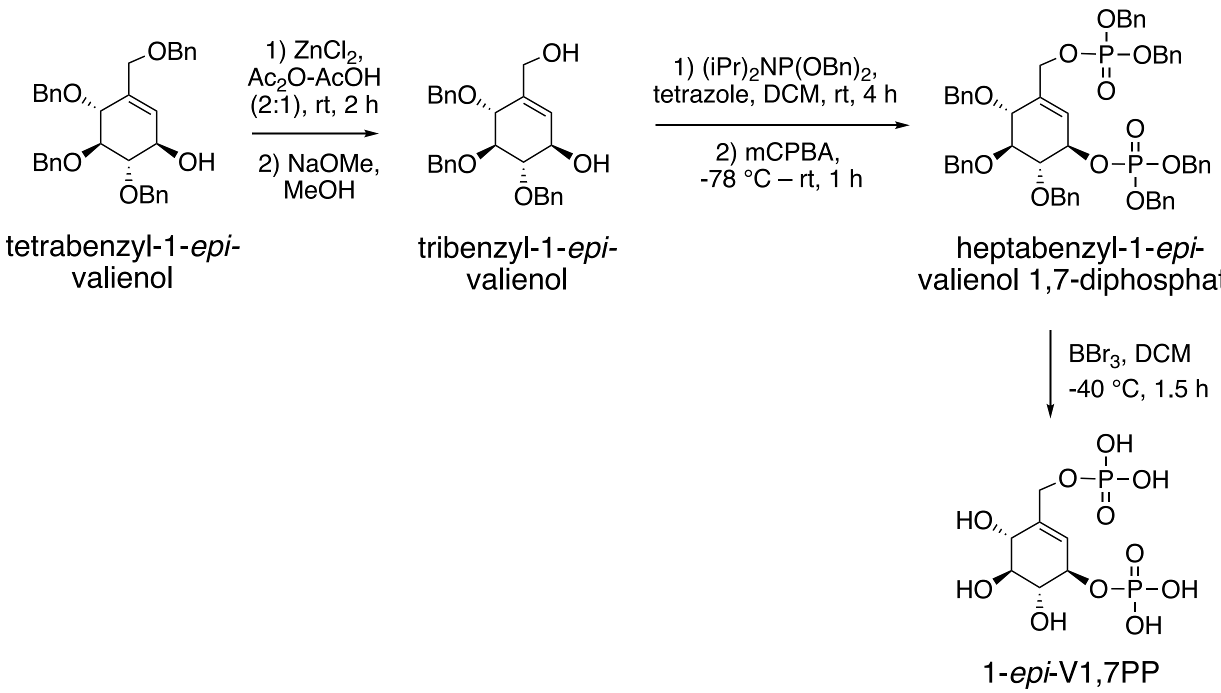

Supplementary Figure 20. Chemical synthesis of 1-*epi*-valienol 1,7-diphosphate (1-*epi*-V1,7PP) from tetrabenzyl-1-*epi*-valienol.

Synthesis of 1-*epi*-valienol 1,7-diphosphate

Synthesis of tribenzyl-1-*epi*-valienol. $ZnCl_2$ (240 mg, 1.84 mmol) was added to a solution of tetrabenzyl-1-*epi*-valienol (99 mg, 0.184 mmol) in Ac_2O–AcOH (2:1; 2 mL), and the reaction mixture was stirred for 2 h at room temperature. The reaction mixture was diluted with water (5 mL) and EtOAc (10 mL), and the organic layer further washed with water (5 mL) and saturated aqueous Na_2CO_3 solution (3 × 5 mL). The organic layer was dried over Na_2SO_4, filtered and concentrated under reduced vacuum to give a crude sample. The crude sample was dissolved in MeOH (2.5 mL) and NaOMe (0.8 mL of 30% solution in MeOH) was added to the reaction mixture. The reaction mixture was stirred for 4 h at room temperature and quenched with water (2 mL) and saturated aqueous NH_4Cl (10 mL). The reaction mixture was diluted with EtOAc (5 mL), and the organic layer further washed with water (5 mL) and brine (5 mL). The organic layer was dried over Na_2SO_4, filtered, and concentrated under reduced vacuum. Column chromatography (silica gel, *n*-hexane–EtOAc = 1:2) yielded tribenzyl-1-*epi*-valienol (50 mg, 60% yield). Analytical data matched reported data.[8]

Tribenzyl-1-*epi*-valienol: ^{1}H NMR (500 MHz, $CDCl_3$): δ 7.39–7.28 (m, 15H), 5.68 (s, 1H), 5.01 (d, J = 3 Hz, 1H), 4.98 (d, J = 3.5 Hz, 1H), 4.96–4.68 (m, 6H), 4.34 (m, 2H), 4.09 (d, J = 6.5 Hz, 2H), 3.88 (dd, J = 9.9, 7.3 Hz, 1H), 3.58 (dd, J = 9.9, 7.3 Hz, 1H); ^{13}C NMR (125 MHz, $CDCl_3$): δ 138.4, 138.2, 138.1, 137.9, 128.6, 128.6, 128.6, 128.5, 128.5, 128.4, 128.1, 128.1, 128.0, 128.0, 127.9, 127.9, 127.8, 127.8, 127.7, 126.5, 123.8, 84.1, 83.7, 80.3, 79.1, 79.1, 78.8, 75.1, 75.0, 74.7, 74.6, 74.0, 72.9, 71.4, 65.0, 63.7, 63.6.

Synthesis of heptabenzyl-1-*epi*-valienol 1,7-diphosphate. A solution of tribenzyl-1-*epi*-valienol (41 mg, 0.92 mmol) in CH_2Cl_2 (2 mL) was added to a solution of dibenzyl *N*,*N*-diisopropylphosphoramidite (0.12 mL, 0.367 mmol) and 1*H*-tetrazole (1.4 mL, 0.643 mmol) in CH_2Cl_2 (4 mL), which had been stirred for 40 min at room temperature under Argon, and the reaction mixture was further stirred for 3 h at room temperature. The reaction mixture was cooled to -78 ℃ and *m*-CPBA (79 mg, 0.456 mmol) was added. After the cooling bath was removed,

stirring of the reaction mixture was continued for an additional 40 min at room temperature. The solvent was removed using a rotary evaporator, and the residue was redissolved in EtOAc (15 mL). The organic layer was washed with 10% aqueous Na_2SO_3 (10 mL), saturated aq. $NaHCO_3$ (10 mL), and brine (10 mL). The organic layer was dried over Na_2SO_4, filtered, and concentrated under reduced vacuum. After column chromatography (silica gel, *n*-hexane–EtOAc = 6:4) to obtain heptabenzyl-1-*epi*-valienol 1,7-diphosphate (62 mg, 70% yield). Analytical data matched reported data.[8]

Heptabenzyl-1-*epi*-valienol 1,7-diphosphate: ^{1}H NMR (500 MHz, $CDCl_3$): δ 7.34–7.24 (m, 35H), 5.79 (s, 1H), 5.03–4.94 (m, 10H), 4.86–4.75 (m, 4H), 4.65 (d, J = 10.7 Hz, 1H), 4.60 (d, J = 8.5 Hz, 1H), 4.44 (dd, J = 12.4, 5.8 Hz, 1H), 4.23 (d, J= 7.6 Hz,1H), 3.79–3.68 (m, 2H); ^{13}C NMR (125 MHz, $CDCl_3$): δ 138.1, 138.1, 137.9, 136.3, 136.2, 135.7, 135.6, 128.6, 128.5, 128.5, 128.4, 128.3, 127.9, 127.9, 127.8, 127.7, 127.5, 125.1, 83.7, 82.2, 82.2, 78.6, 78.2, 78.2, 75.4, 75.3, 75.0, 69.4, 69.4, 69.3, 69.3, 66.6, 66.6; ^{31}P NMR (202 MHz, $CDCl_3$): δ -0.90, -1.20.

Synthesis of 1-*epi*-valienol 1,7-diphosphate. BBr_3 (0.23 mL of 1.0 M solution in CH_2Cl_2, 0.45 mmol) was added to a solution of heptabenzyl-1-*epi*-valienol 1,7-diphosphate (25 mg, 0.026 mmol) in CH_2Cl_2 (1 mL) and the reaction mixture was stirred for 90 min at –40 °C. Water (1 mL) was added to the reaction mixture at the same temperature, and the pH of the solution was adjusted to weak basic using saturated aqueous $NaHCO_3$ solution. The mixture was then partitioned between H_2O (5 mL) and CH_2Cl_2 (5 mL). The aqueous fraction was collected and subjected to Biogel P-2 Gel fine column chromatography (8 cm × 0.5 cm) and eluted with H_2O. Fractions containing the product were pooled and dried. The resulting white solid was dissolved in H_2O and desalted by passing it through a Sephadex LH-20 column using H_2O as an eluent. Fractions containing the product were combined and lyophilized to give 1-*epi*-valienol 1,7-diphosphate (4.9 mg, 56% yield). Analytical data matched reported data.[8]

1-*epi*-Valienol 1,7-diphosphate: ^{1}H NMR (500 MHz, D_2O): δ 5.59 (s,1 H), 4.44 (t, J = 7.15 Hz, 1H), 4.36 (t, J = 11.5 Hz, 1H), 4.16 (d, J = 6.6 Hz, 1H), 4.04 (dd, J = 12.7, 6.1 Hz, 1H), 3.52–3.43 (m, 2H); ^{31}P NMR (202 MHz, D_2O): δ 3.92, 3.70.

NMR data for 4-aminoDGG (Supplementary Figures 21 and 22)

^{1}H NMR (700 MHz, D_2O): δ 5.37-5.34 (m, 2H), 5.17/4.59 (d, J = 3.5/7.7 Hz, 1H), 3.92-3.50 (m, 15H), 2.82 (t, J = 13.3 Hz, 1H), 1.27 (d, J = 6.3 Hz, 3H); ^{13}C NMR (175 MHz, D_2O): δ 99.6, 99.4, 99.2, 95.6, 91.2, 76.9, 76.5, 76.1, 74.4, 73.2, 73.1, 71.2, 71.4, 71.3, 71.1, 70.9, 60.3, 56.8, 16.5.

NMR data for valienol 7-phosphate (Supplementary Figures 23, 24 and 25)

^{1}H NMR (700 MHz, D_2O): δ 6.86 (d, J = 5.6 Hz, 1H), 4.44 (dd, J = 13.3, 8.4 Hz, 1H), 4.22 (t, J = 4.9 Hz, 1H), 4.17 (dd, J = 13.3, 6.3 Hz, 1H), 4.11 (d, J = 8.4 Hz, 1H), 3.65 (m, 1H), 3.57 (dd, J = 10.5, 4.2 Hz, 1H); ^{13}C NMR (175 MHz, D_2O): δ 141.0, 122.9, 71.9, 71.4, 66.0, 63.9, 59.5; ^{31}P NMR (162 MHz, D_2O): 4.47.

NMR data for acarbose 7-phosphate (Supplementary Figures 26, 27 and 28)

^{1}H NMR (700 MHz, D_2O): δ 5.92 (d, J = 4.9, 1H), 5.34 (d, J = 4.2 Hz, 1H), 5.16/4.59 (d, J = 3.5/7.7 Hz, 1H), 4.42 (m, 1H), 4.24 (m, 1H), 4.06 (d, J = 7.0 Hz, 1H), 3.90-3.19 (m, 19H), 2.53 (t, J = 13.3 Hz, 1H), 1.30 (d, J = 6.3 Hz, 3H); ^{13}C NMR (175 MHz, D_2O): δ 138.5, 123.3, 99.5, 95.6, 91.2, 76.9, 76.8, 76.7, 76.1, 74.4, 73.8, 73.2, 72.6, 72.3, 71.7, 71.4, 71.3, 71.1, 71.0, 70.5, 70.2, 68.7, 64.6, 60.5, 60.3, 55.8, 17.1; ^{31}P NMR (162 MHz, D_2O): 2.65.

NMR data for valienol 1-phosphate (Supplementary Figures 29, 30 and 31)

^{1}H NMR (700 MHz, D_2O): δ 6.00 (d, J = 5.6 Hz, 1H), 4.85 (m, 1H), 4.31 (d, J = 19.6 Hz, 1H), 4.23 (d, J = 19.6 Hz, 1H), 4.12 (d, J = 9.8 Hz, 1H), 3.91 (t, J = 14.7 Hz, 1H), 3.64 (dd, J = 14.7, 5.6 Hz,

1H); ^{13}C NMR (175 MHz, D_2O): δ 141.7, 121.9, 73.0, 72.0, 70.6, 69.2, 61.2; ^{31}P NMR (162 MHz, D_2O): 3.20.

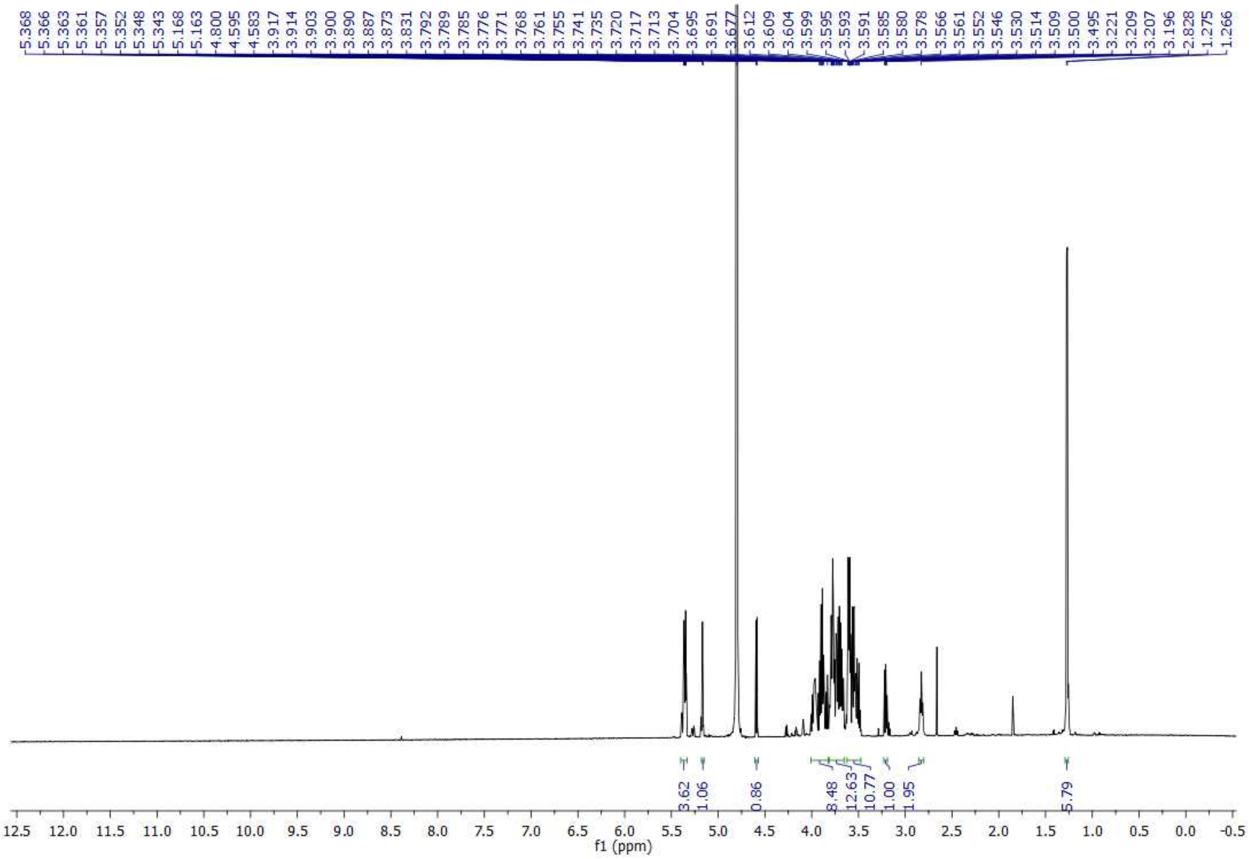

Supplementary Figure 21. ^1H NMR of 4-aminoDGG used as a substrate or standard (700 MHz, D_2O).

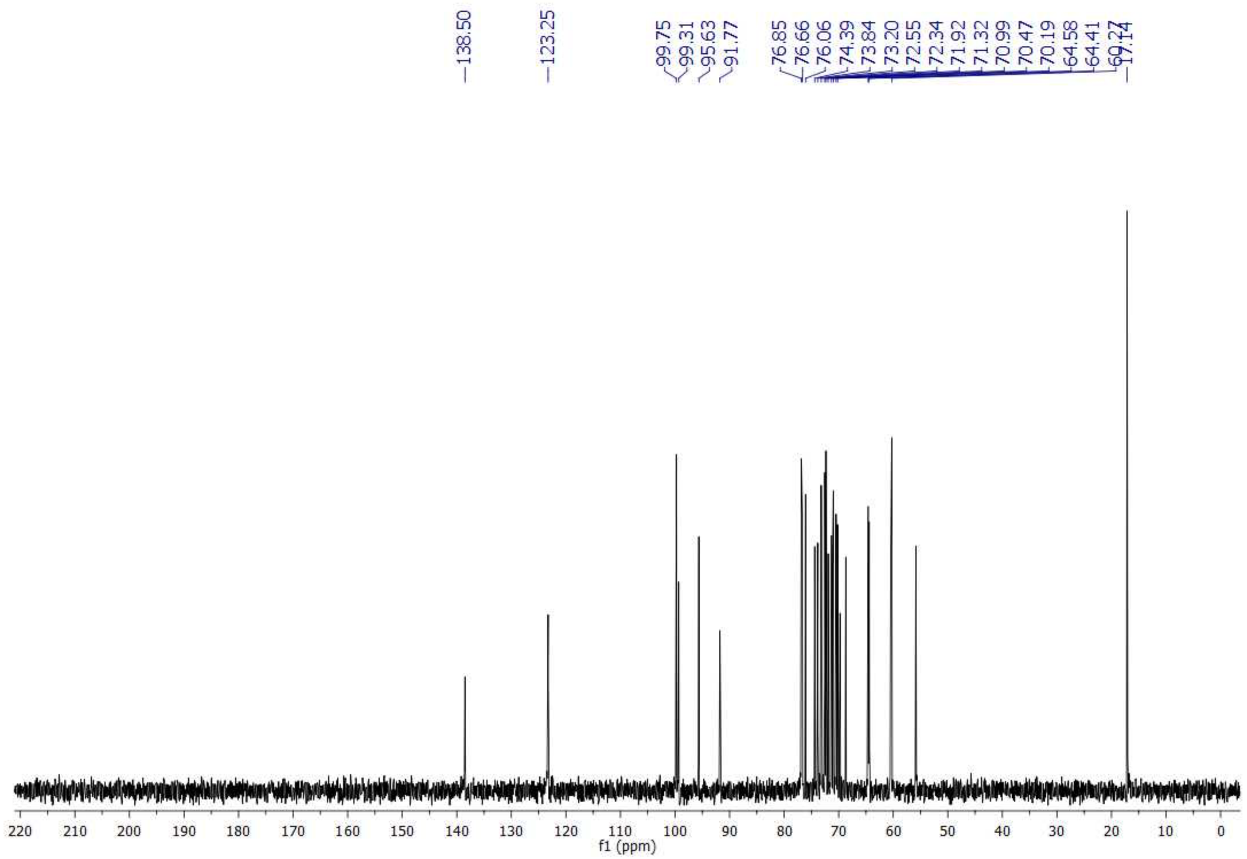

Supplementary Figure 22. ^{13}C NMR of 4-aminoDGG used as a substrate or standard (175 MHz, D_2O).

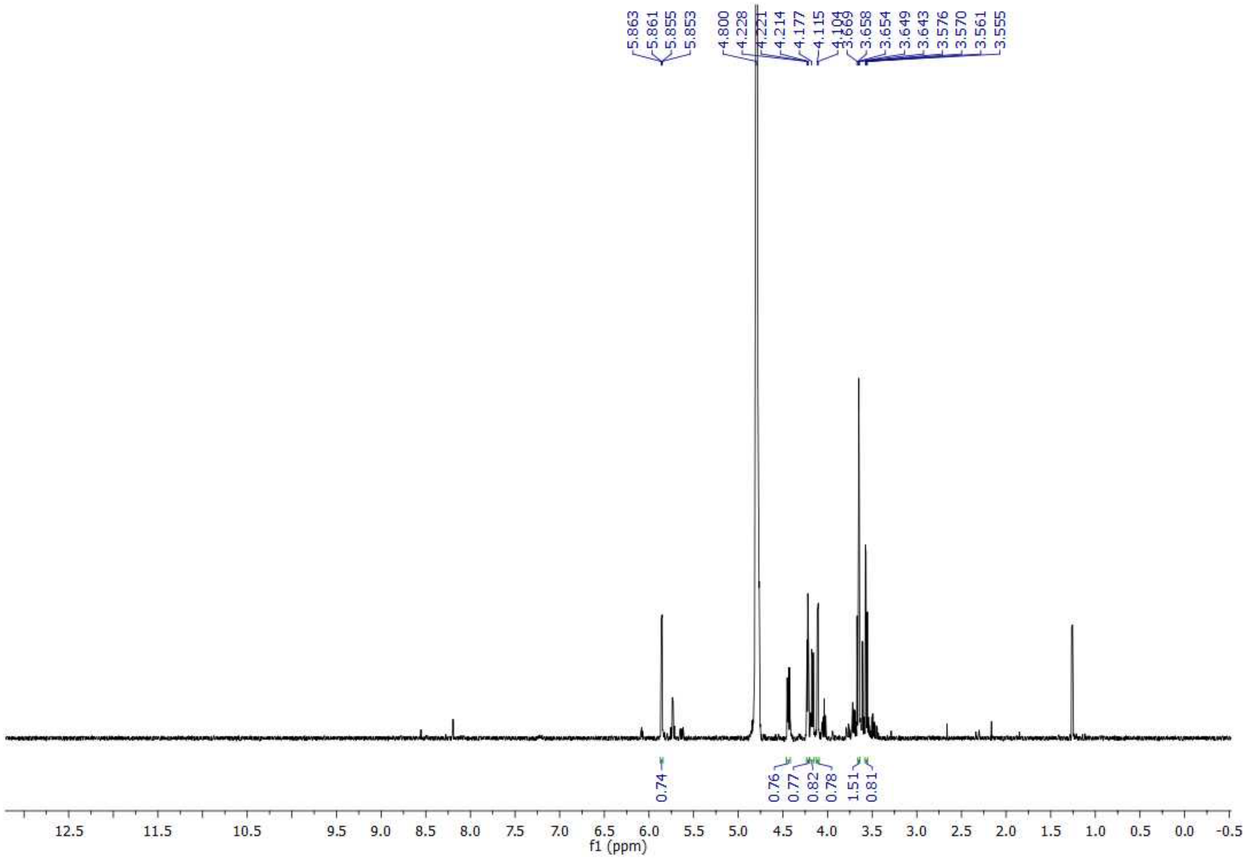

Supplementary Figure 23. ^{1}H NMR of valienol 7-phosphate used as a substrate and/or standard (700 MHz, D_2O).

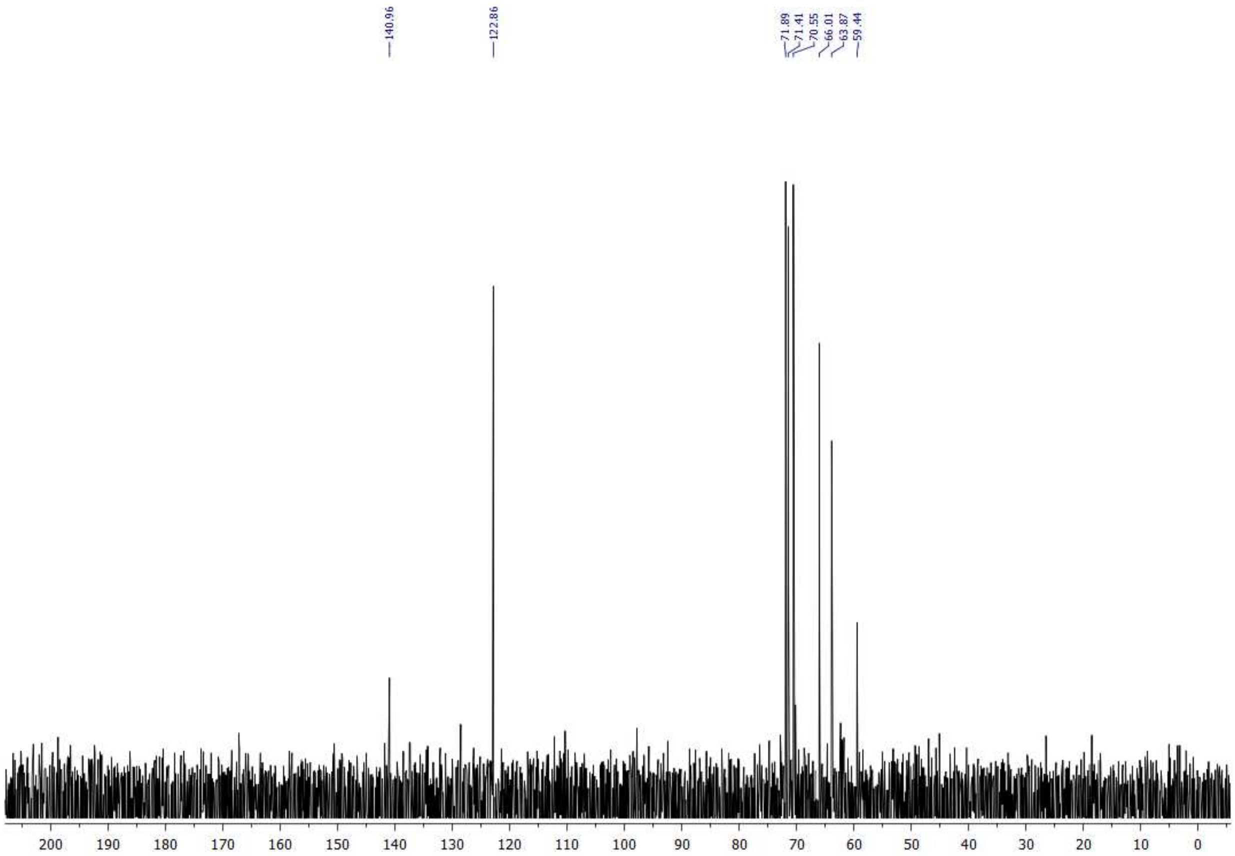

Supplementary Figure 24. ^{13}C NMR of valienol 7-phosphate used as a substrate and/or standard (175 MHz, D_2O).

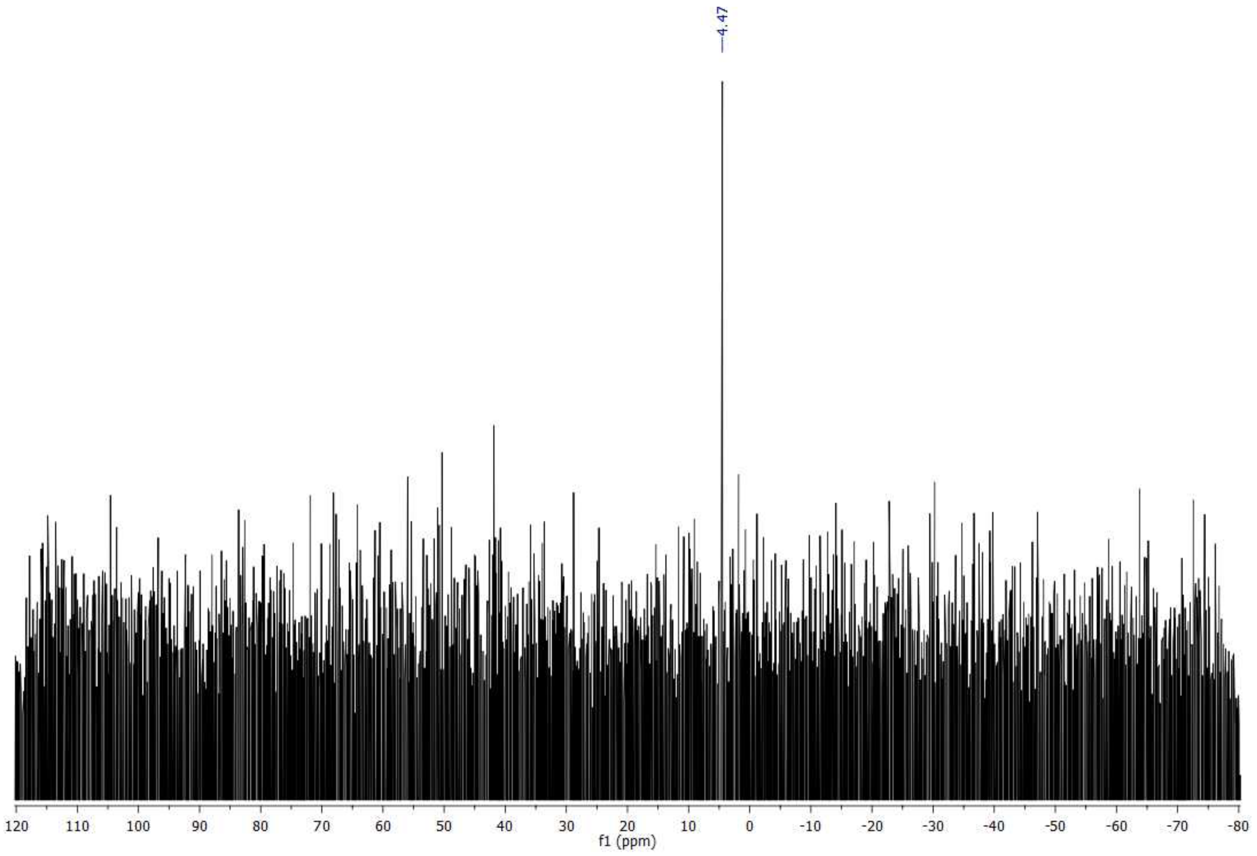

Supplementary Figure 25. ^{31}P NMR of valienol 7-phosphate used as a substrate and/or standard (162 MHz, D_2O).

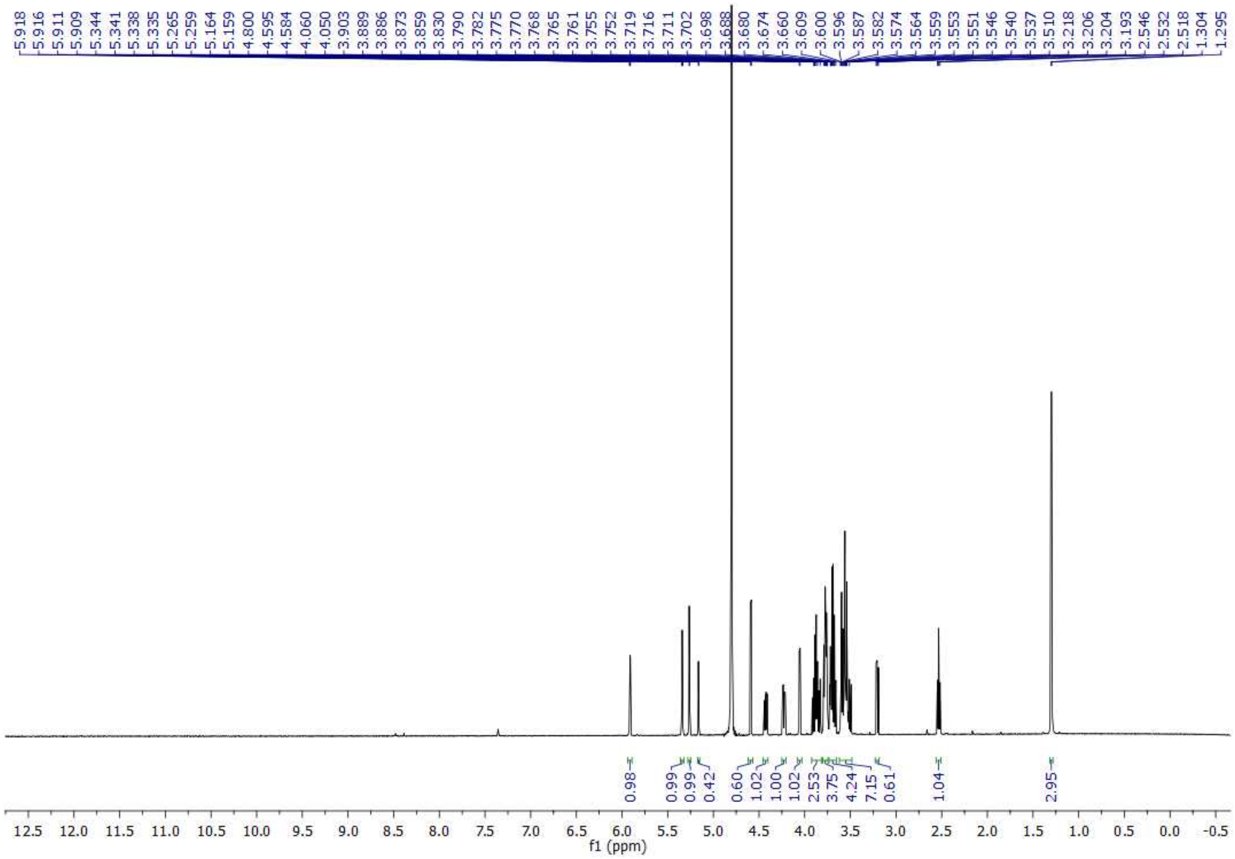

Supplementary Figure 26. ^1H NMR of acarbose 7-phosphate used as a substrate and/or standard (700 MHz, D_2O).

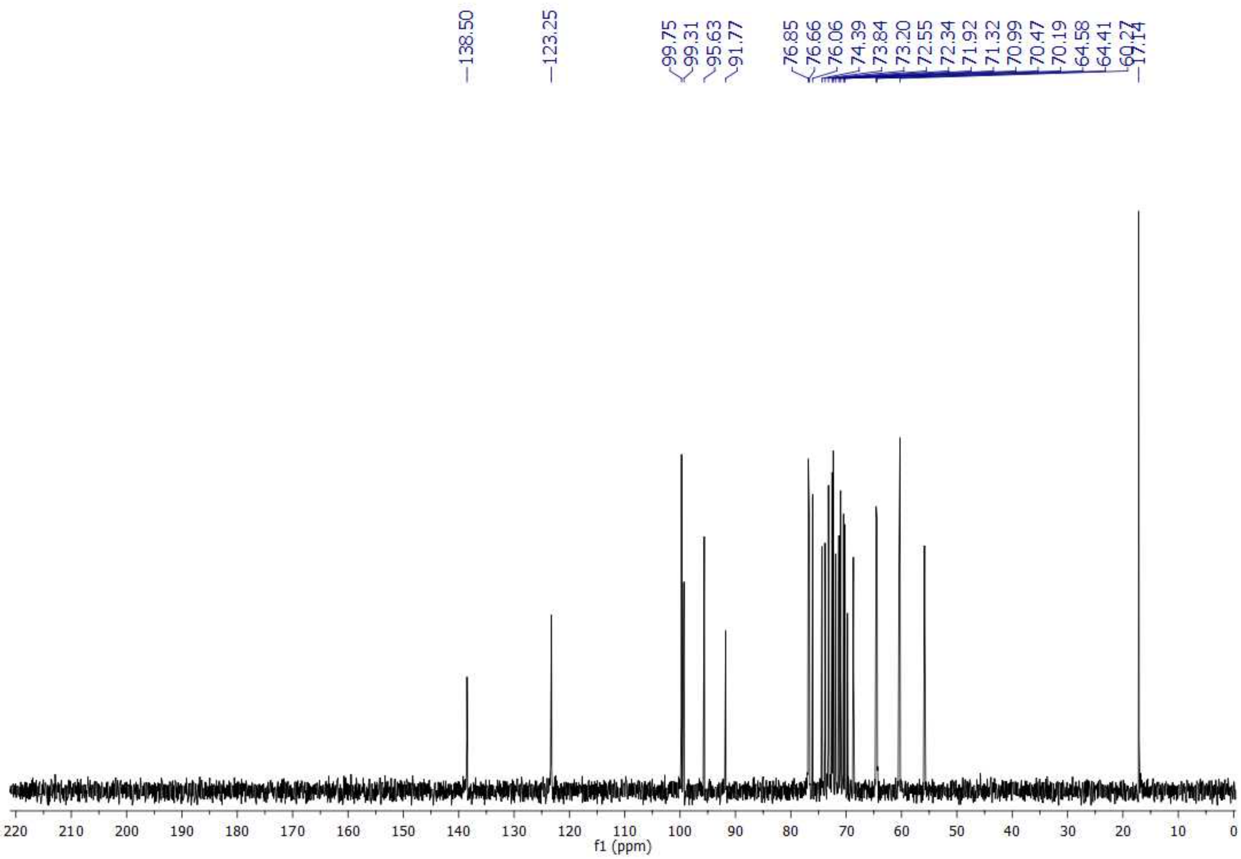

Supplementary Figure 27. ^{13}C NMR of acarbose 7-phosphate used as a substrate and/or standard (175 MHz, D_2O).

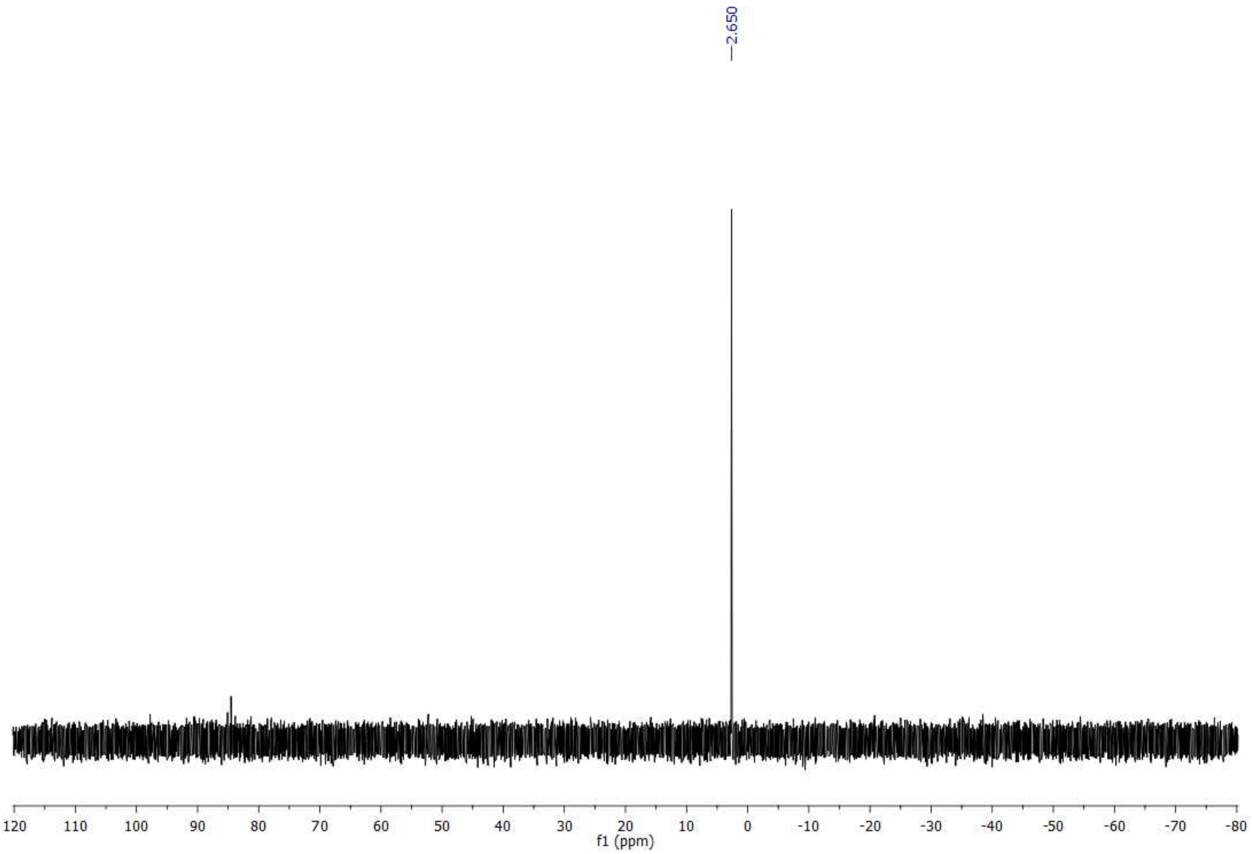

Supplementary Figure 28. ^{31}P NMR of acarbose 7-phosphate used as a substrate and/or standard (162 MHz, D_2O).

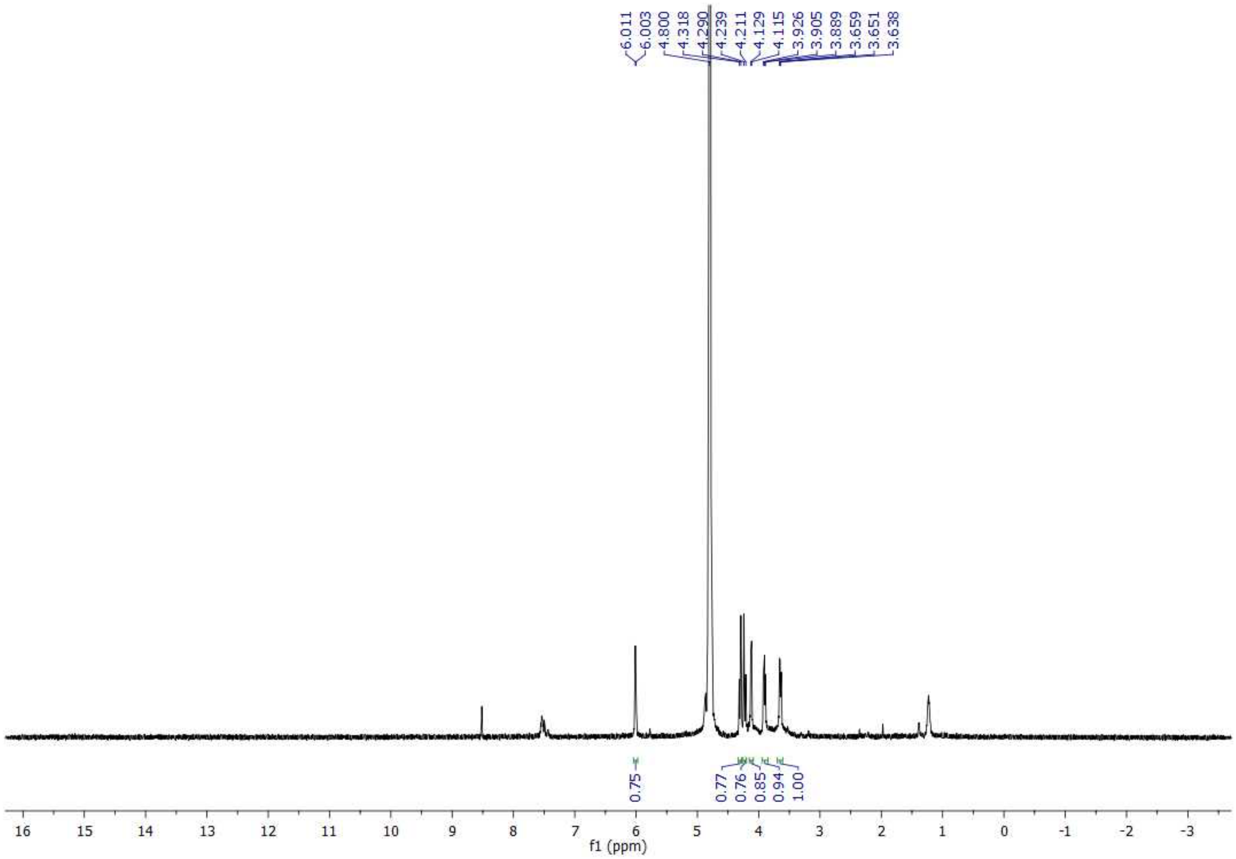

Supplementary Figure 29. ^{1}H NMR of valienol 1-phosphate (**15**) used as a substrate and/or standard (700 MHz, D_2O).

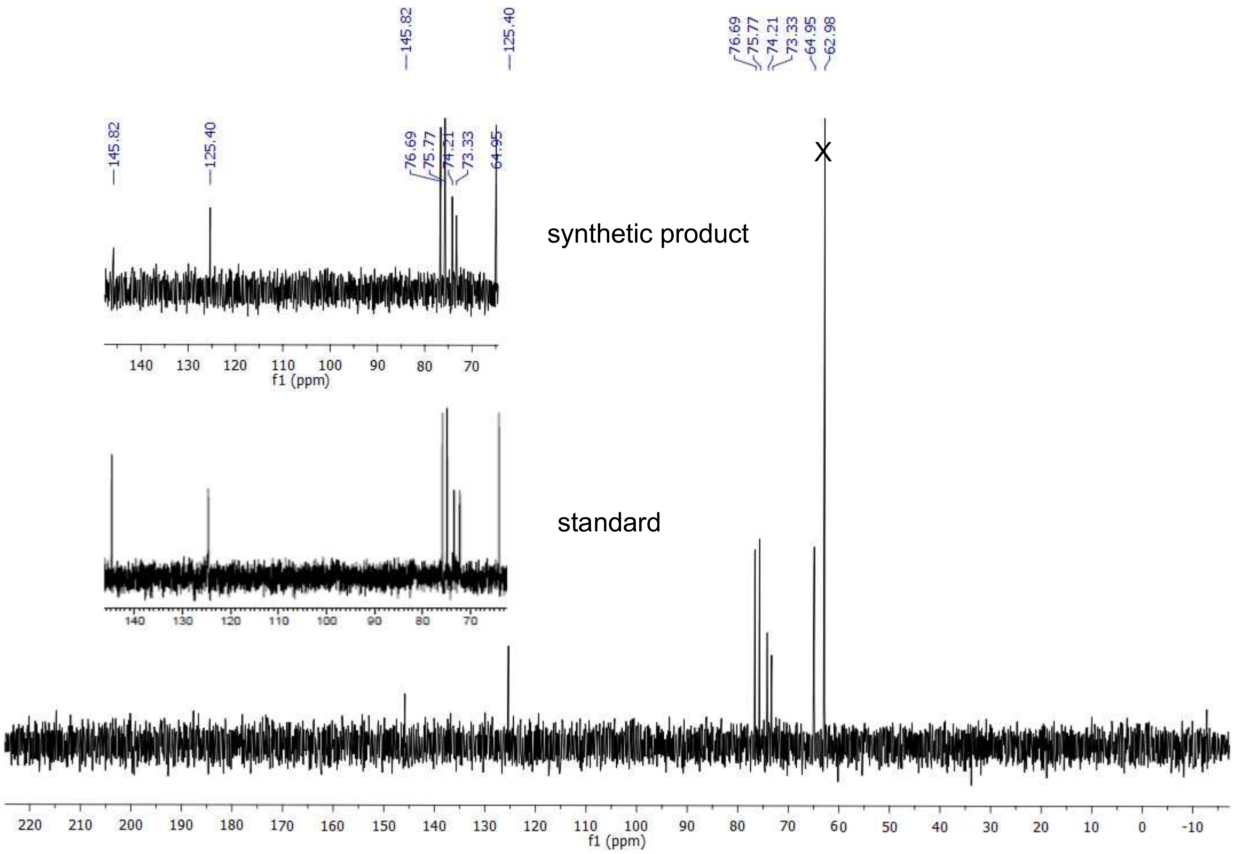

Supplementary Figure 30. ^{13}C NMR of valienol 1-phosphate (**15**) used as a substrate and/or standard (175 MHz, D_2O). X, impurity.

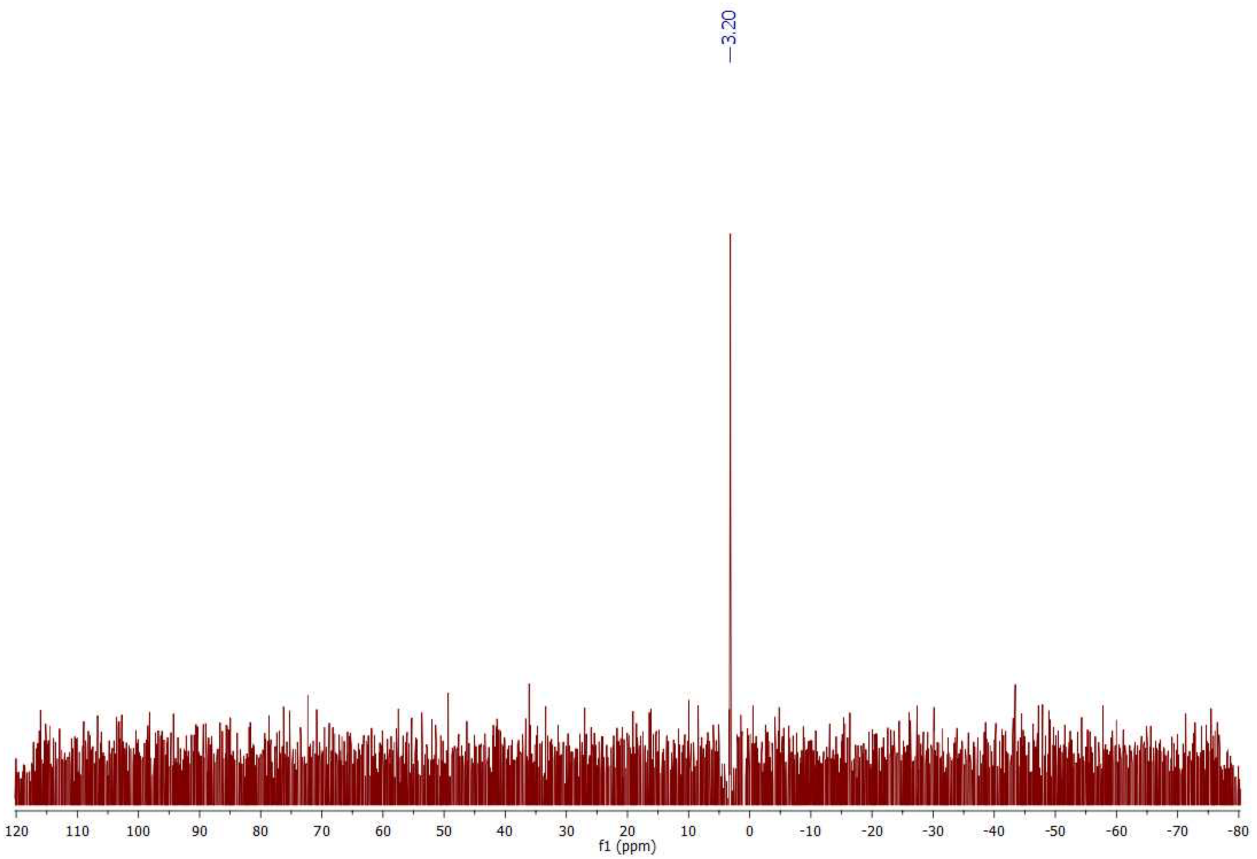

Supplementary Figure 31. ^{31}P NMR of valienol 1-phosphate (**15**) used as a substrate and/or standard (162 MHz, D_2O).

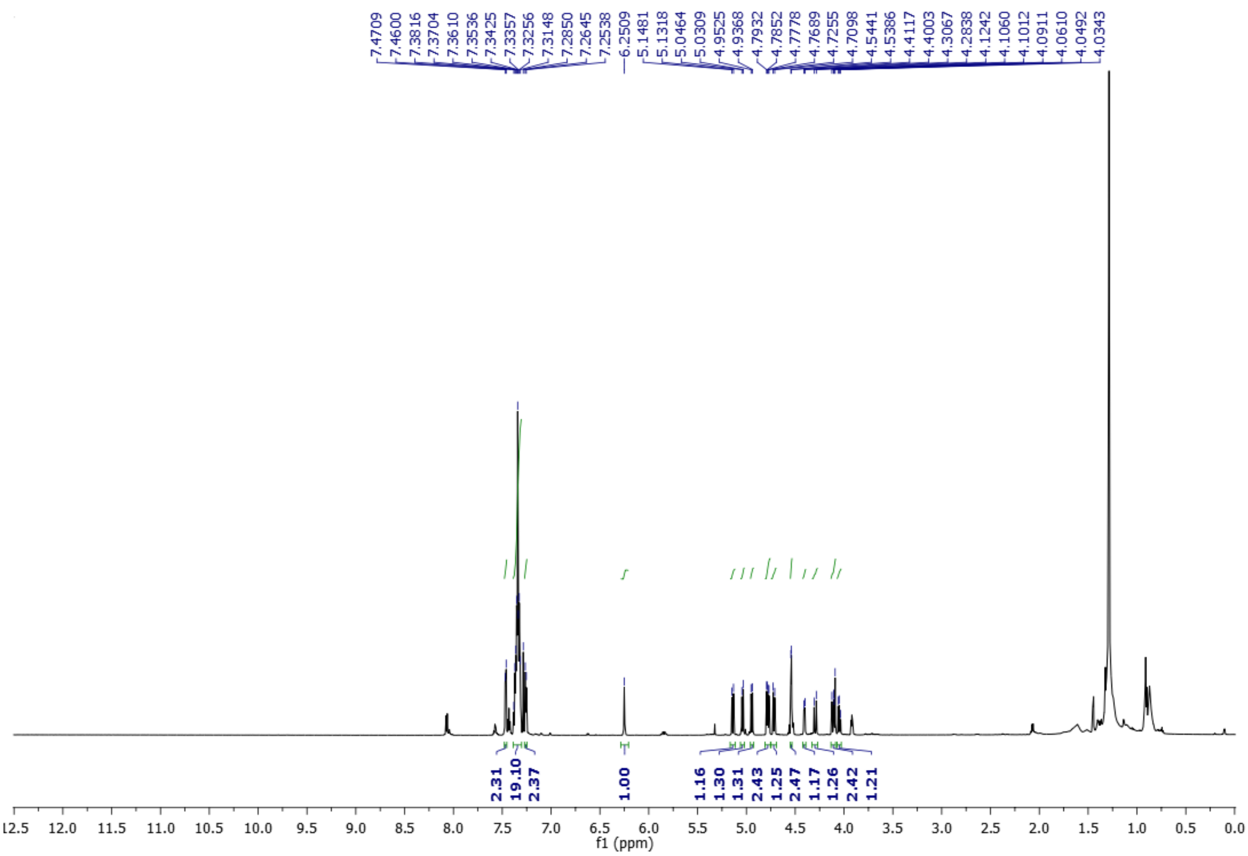

Supplementary Figure 32. ^{1}H NMR spectrum of tetrabenzylvalienone (700 MHz, $CDCl_3$).

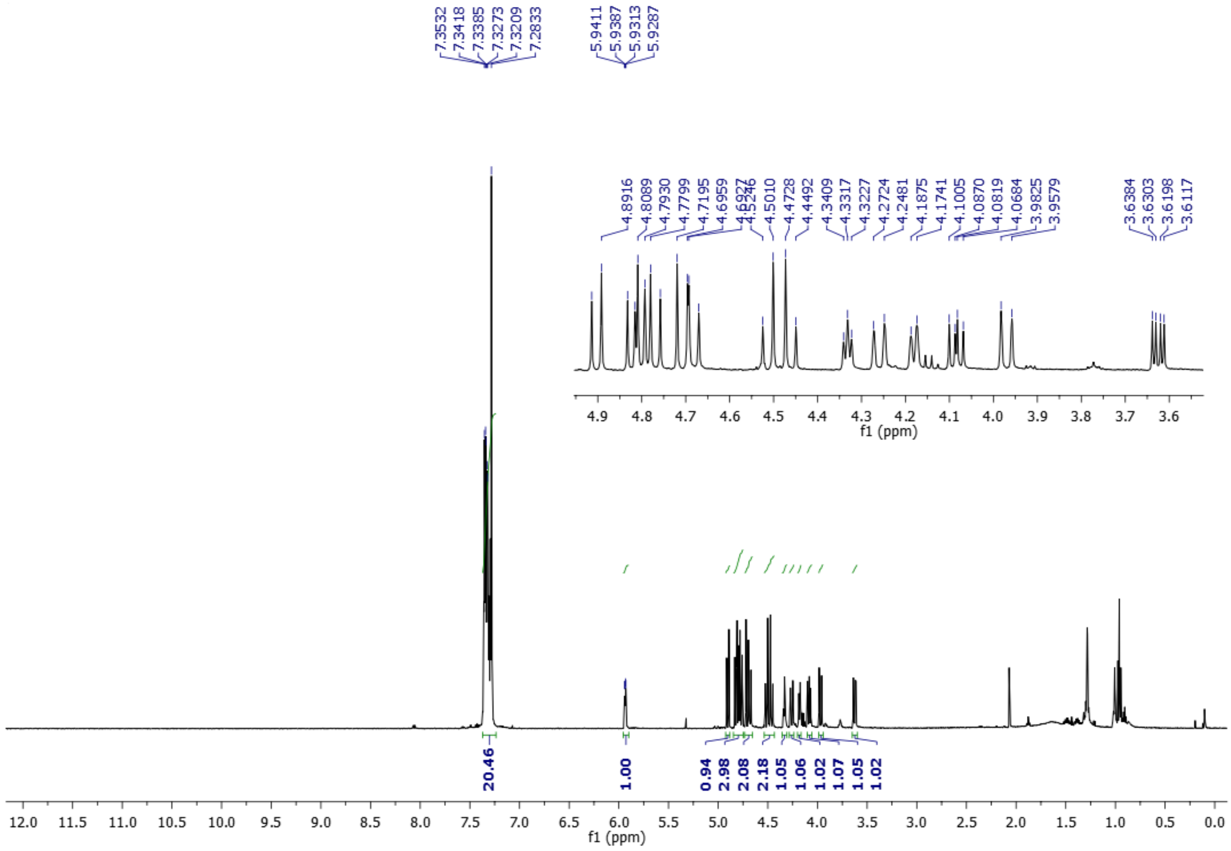

Supplementary Figure 33. ^{1}H NMR spectrum of tetrabenzylvalienol (500 MHz, $CDCl_3$).

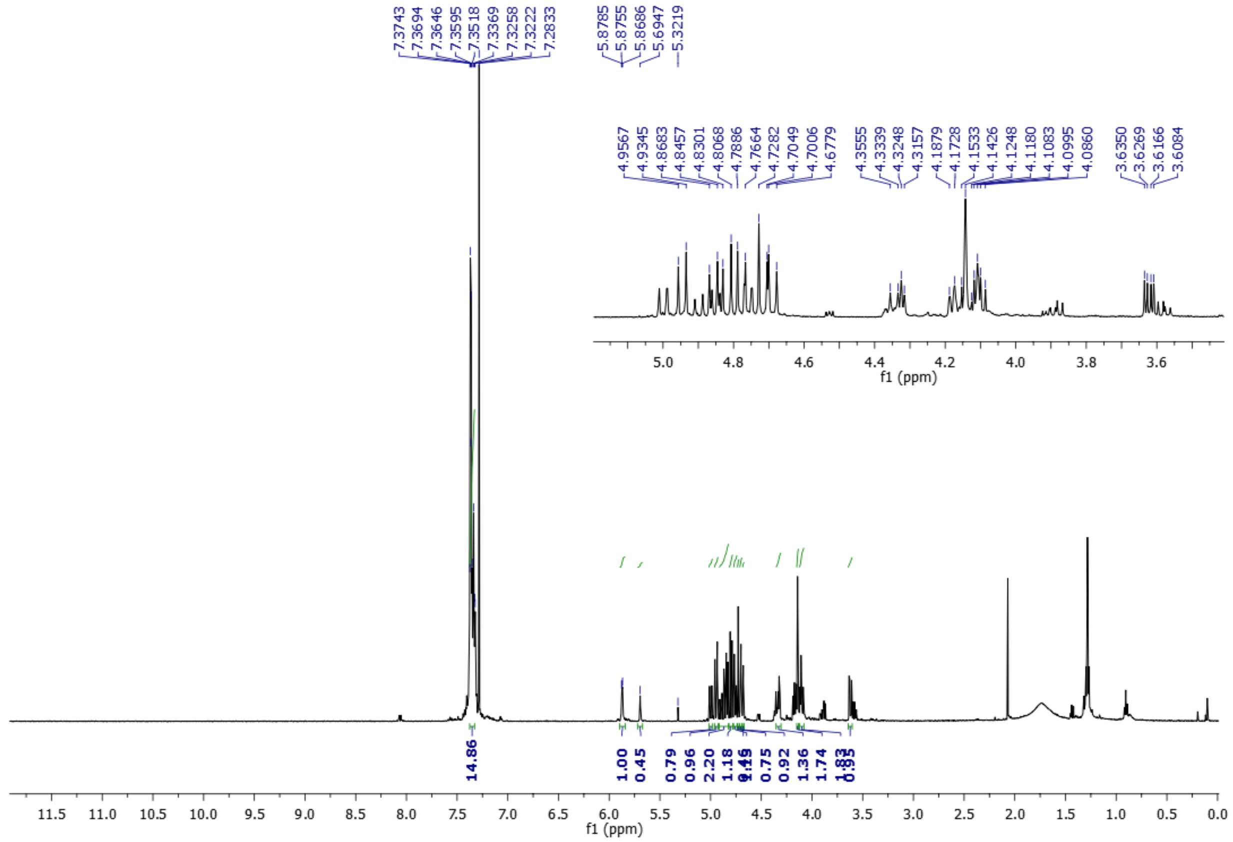

Supplementary Figure 34. ^{1}H NMR spectrum of tribenzylvalienol (500 MHz, $CDCl_3$).

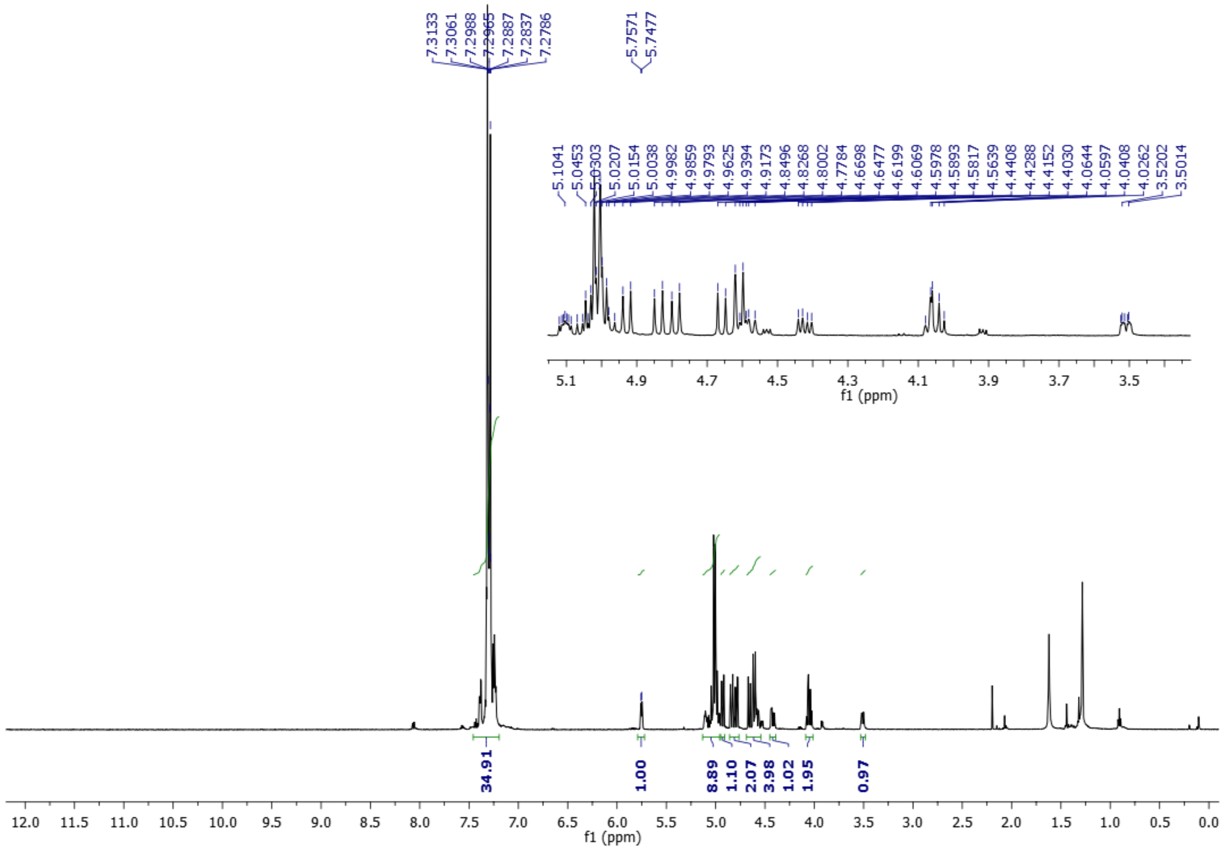

Supplementary Figure 35. ^{1}H NMR spectrum of heptabenzylvalienol 1,7 diphosphate (500 MHz, $CDCl_3$).

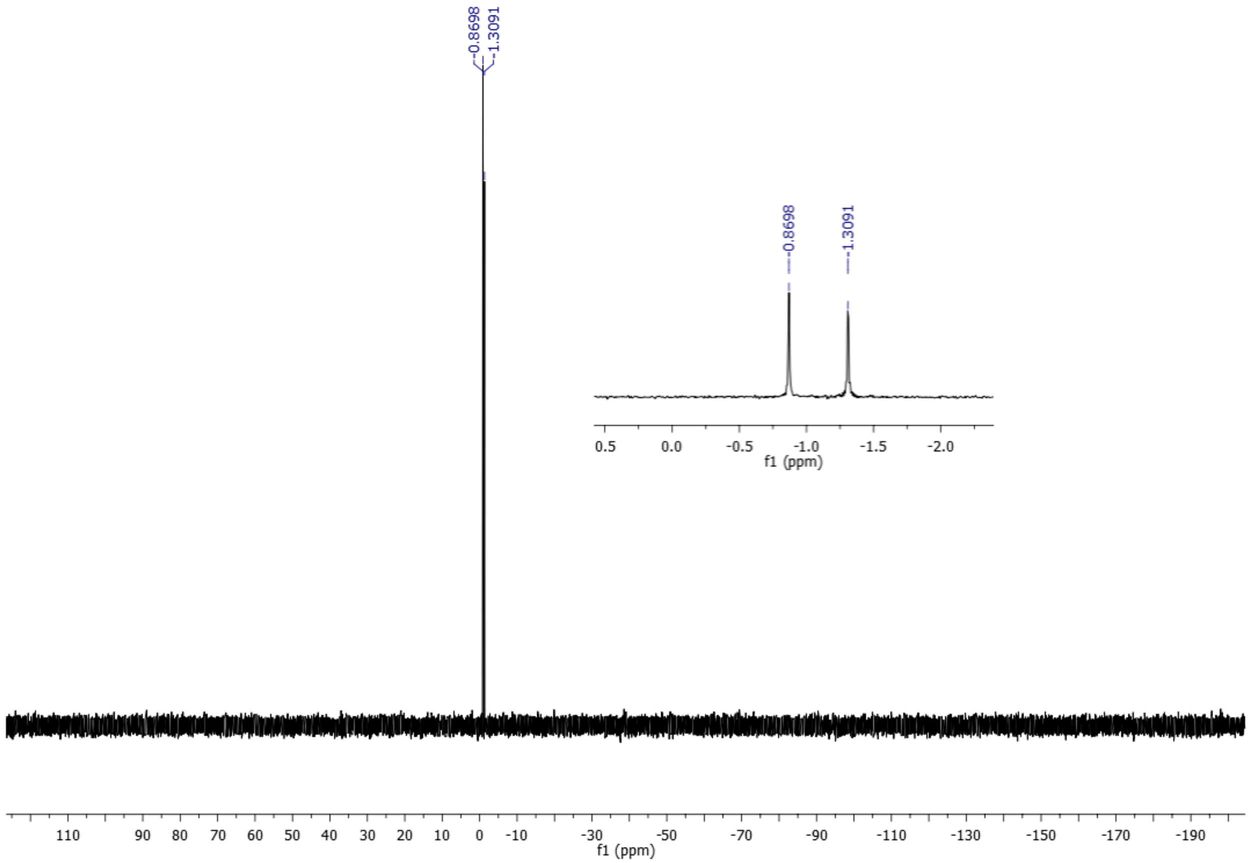

Supplementary Figure 36. ^{31}P NMR spectrum of heptabenzylvalienol 1,7 diphosphate (202 MHz, $CDCl_3$).

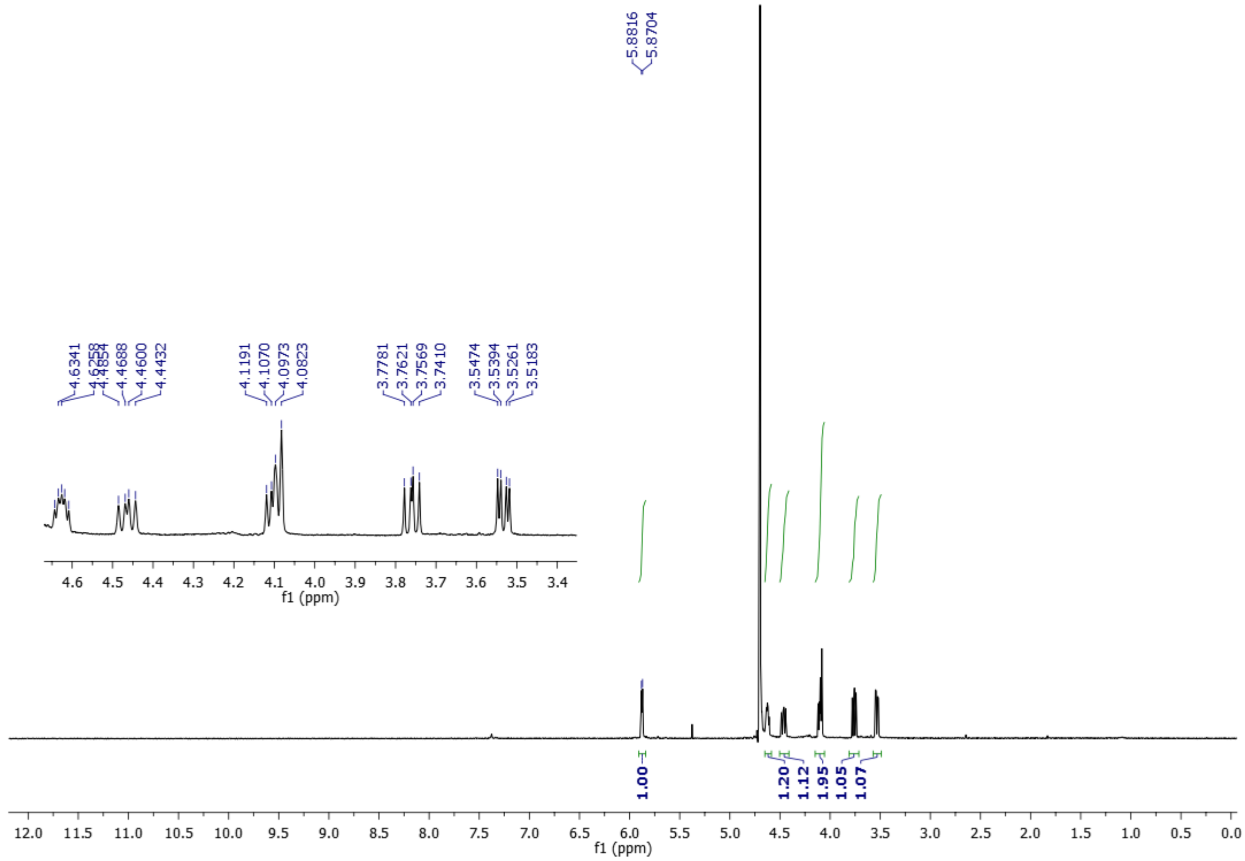

Supplementary Figure 37. ^{1}H NMR spectrum of valienol 1,7-diphosphate (500 MHz, D_2O).

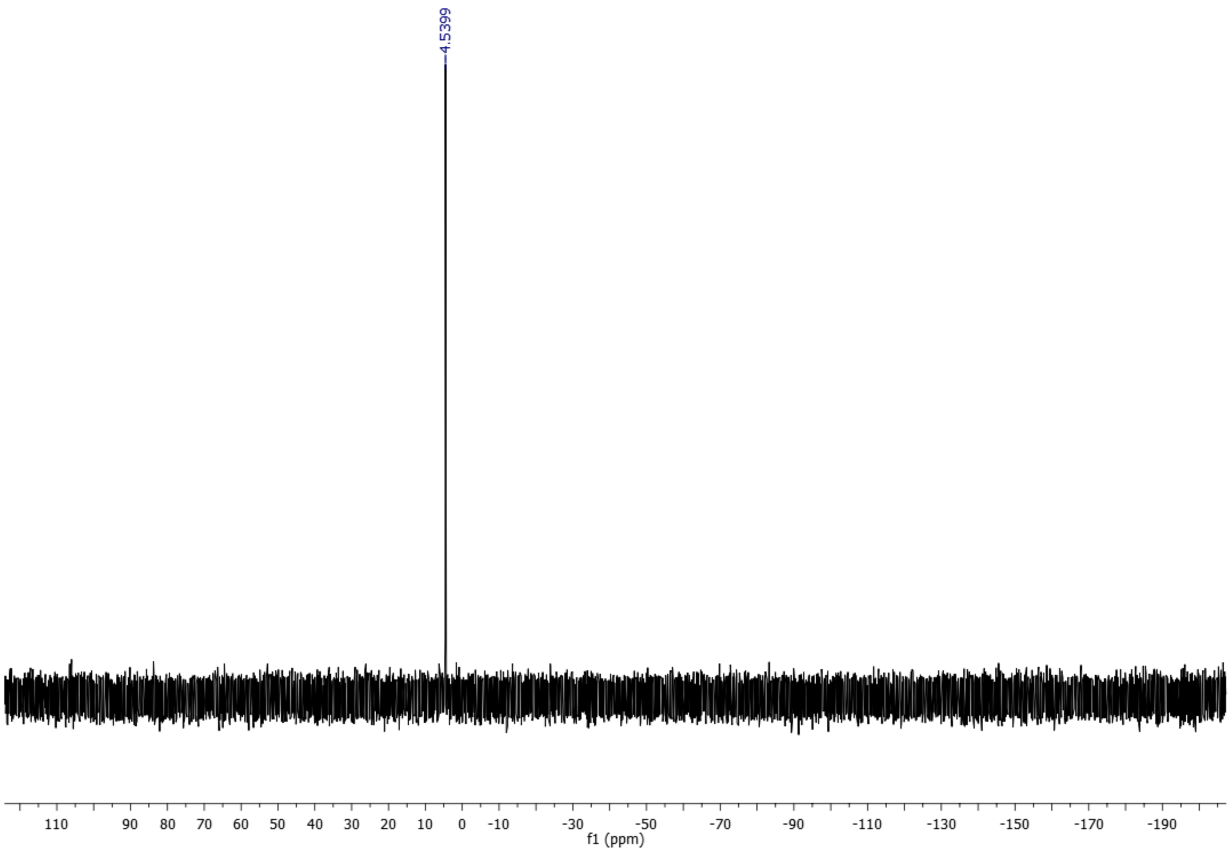

Supplementary Figure 38. ^{31}P NMR spectrum of valienol 1,7-diphosphate (202 MHz, D_2O).

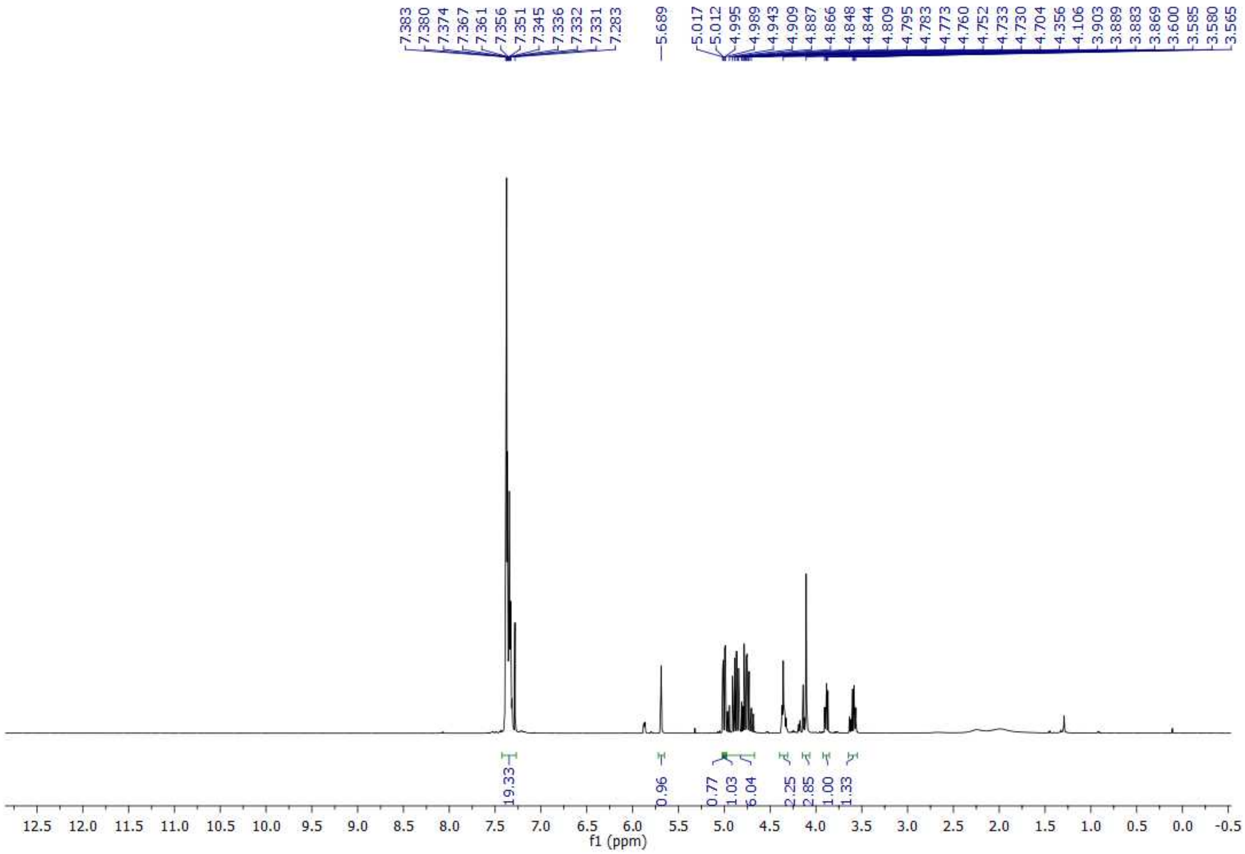

Supplementary Figure 39. ^{1}H NMR spectrum of tribenzyl-1-*epi*-valienol 1,7-diphosphate (500 MHz, $CDCl_3$).

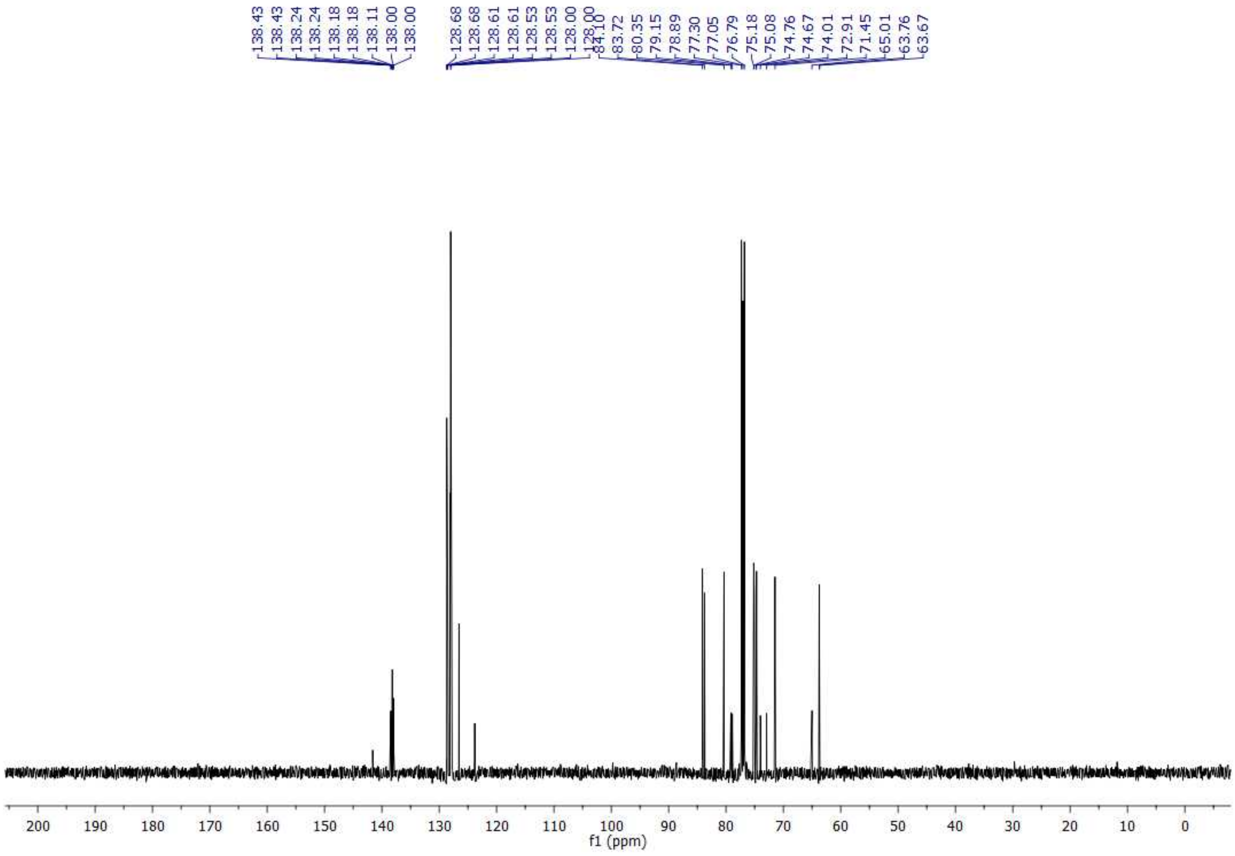

Supplementary Figure 40. ^{13}C NMR spectrum of tribenzyl-1-*epi*-valienol 1,7-diphosphate (125 MHz, $CDCl_3$).

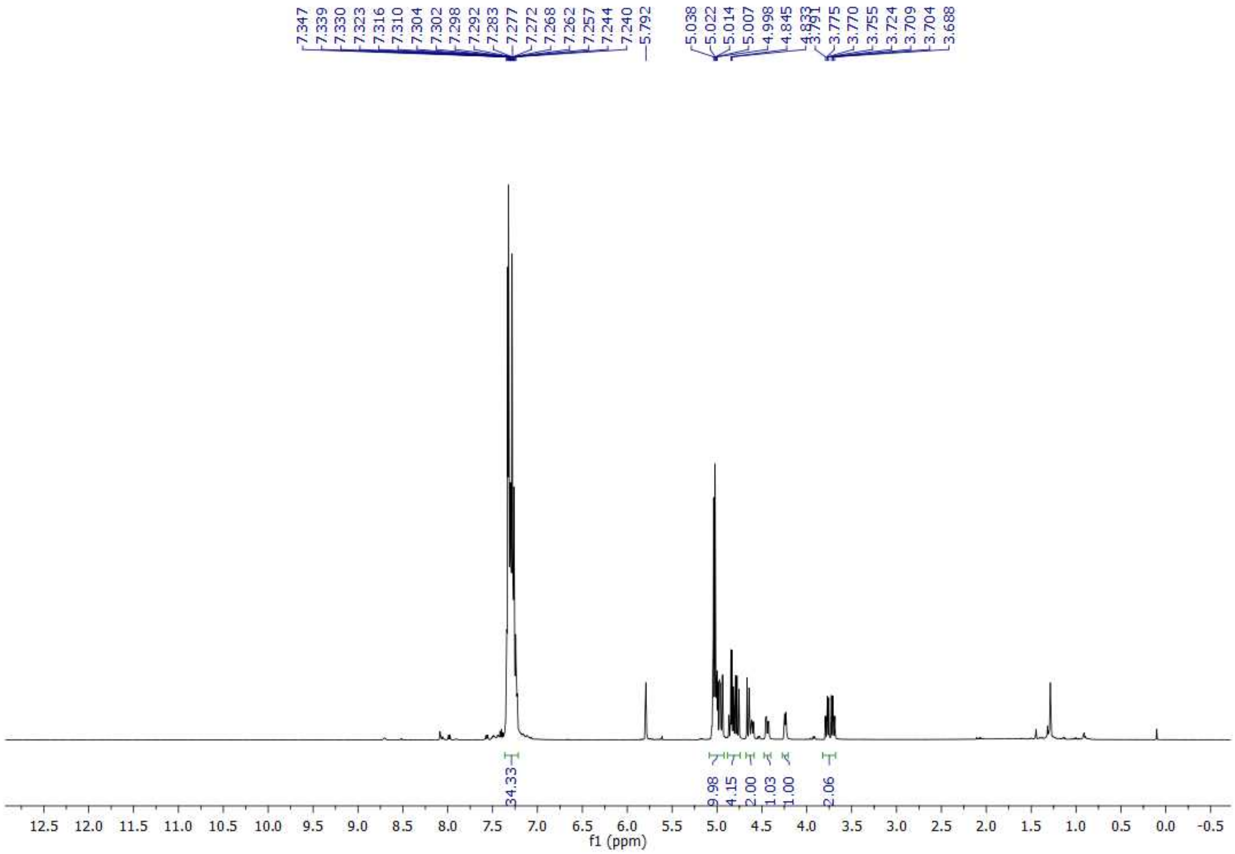

Supplementary Figure 41. ^1H NMR spectrum of heptabenzyl-1-*epi*-valienol 1,7-diphosphate (500 MHz, $CDCl_3$).

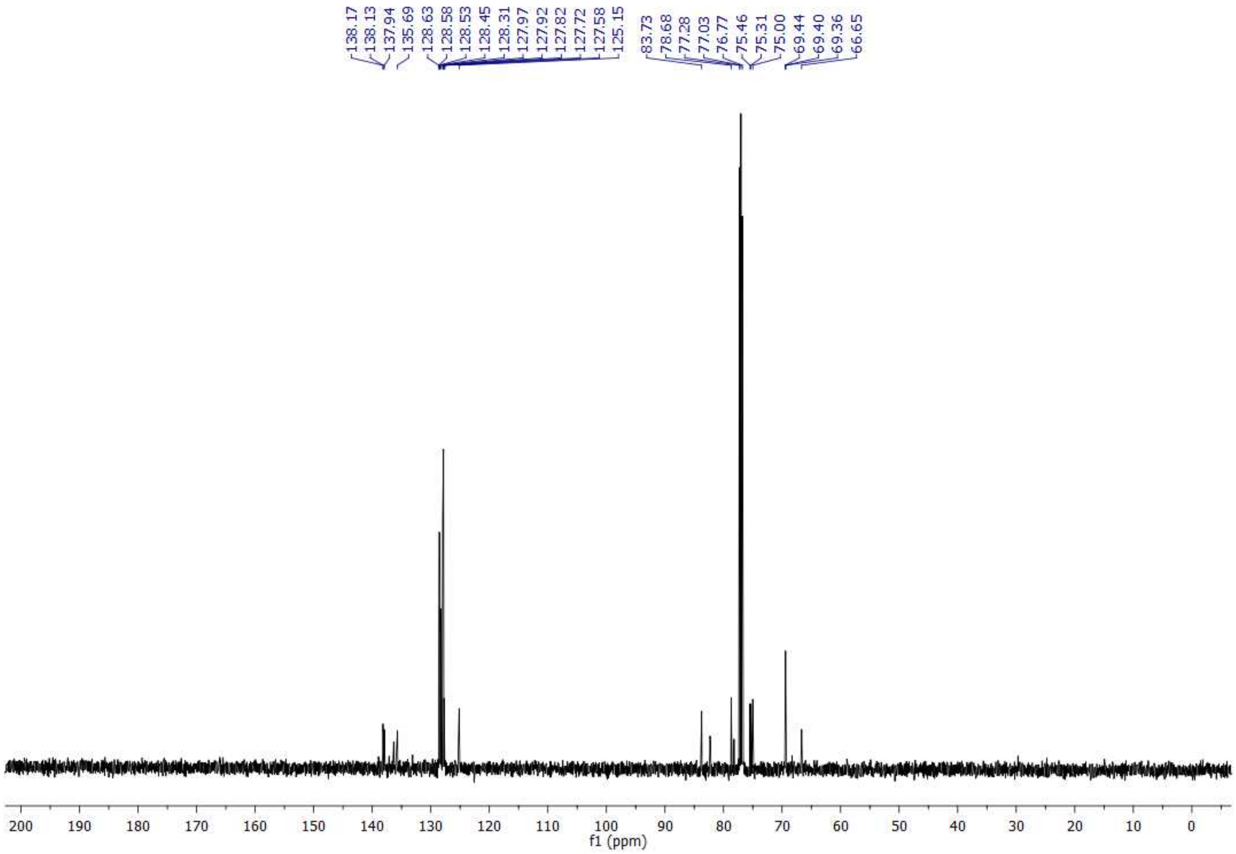

Supplementary Figure 42. ^{13}C NMR spectrum of heptabenzyl-1-*epi*-valienol 1,7-diphosphate (125 MHz, $CDCl_3$).

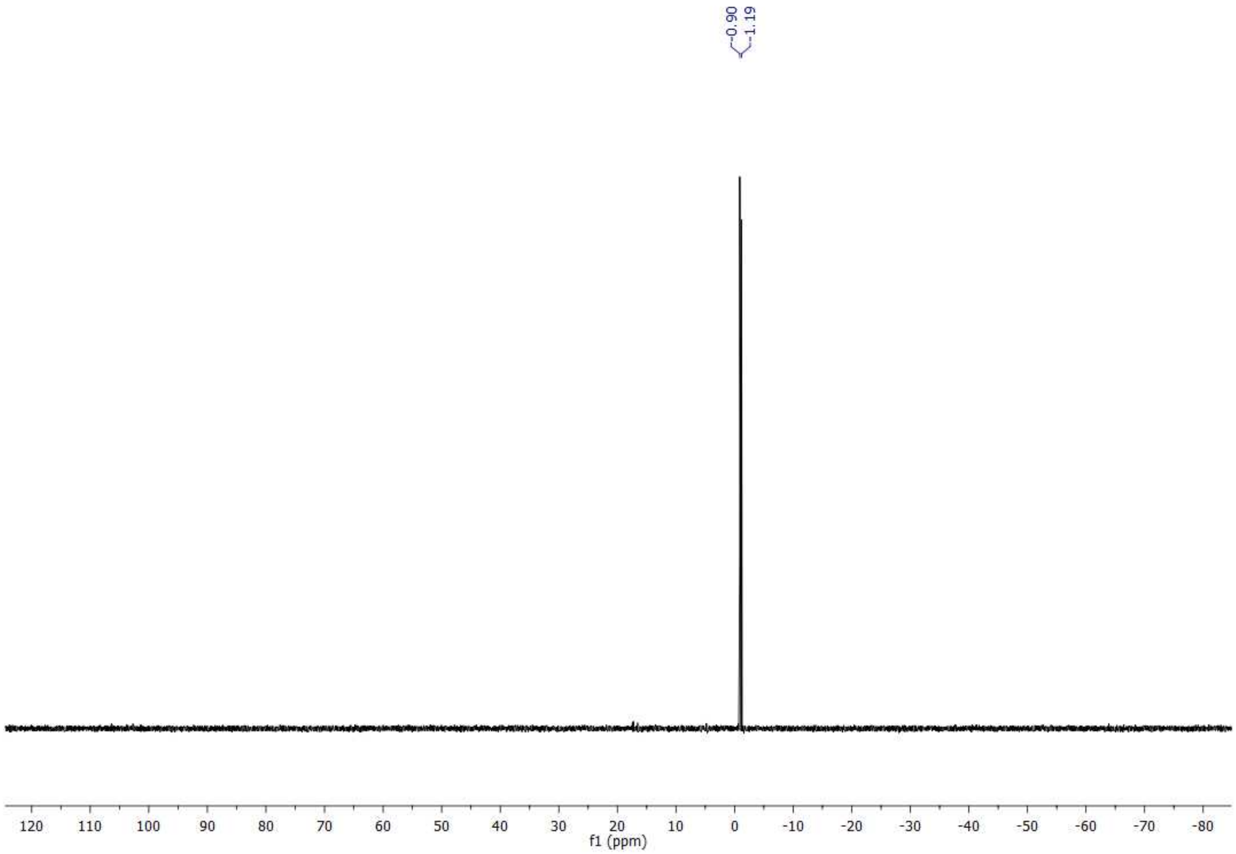

Supplementary Figure 43. ^{31}P NMR spectrum of heptabenzyl-1-*epi*-valienol 1,7-diphosphate (202 MHz, $CDCl_3$).

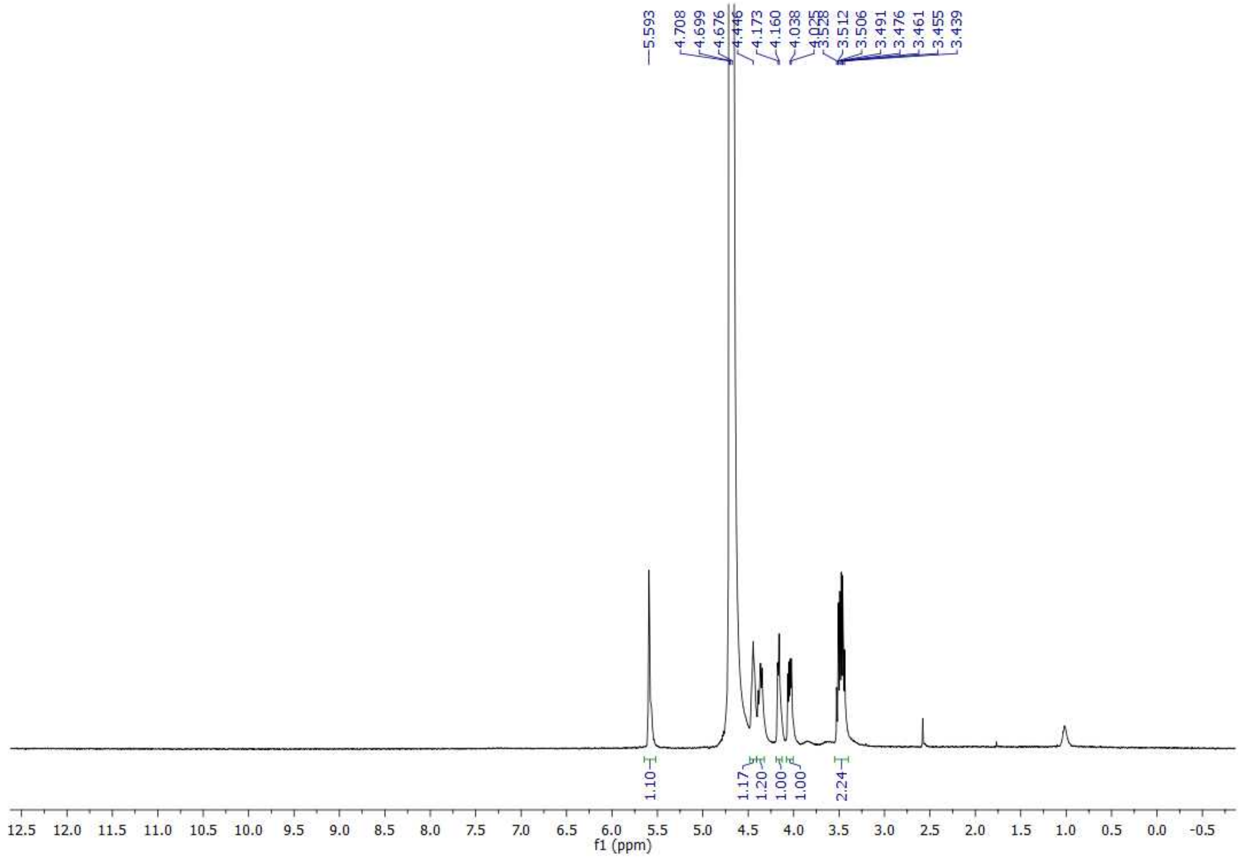

Supplementary Figure 44. ^{1}H NMR spectrum of 1-*epi*-valienol 1,7-diphosphate (500 MHz, D_2O).

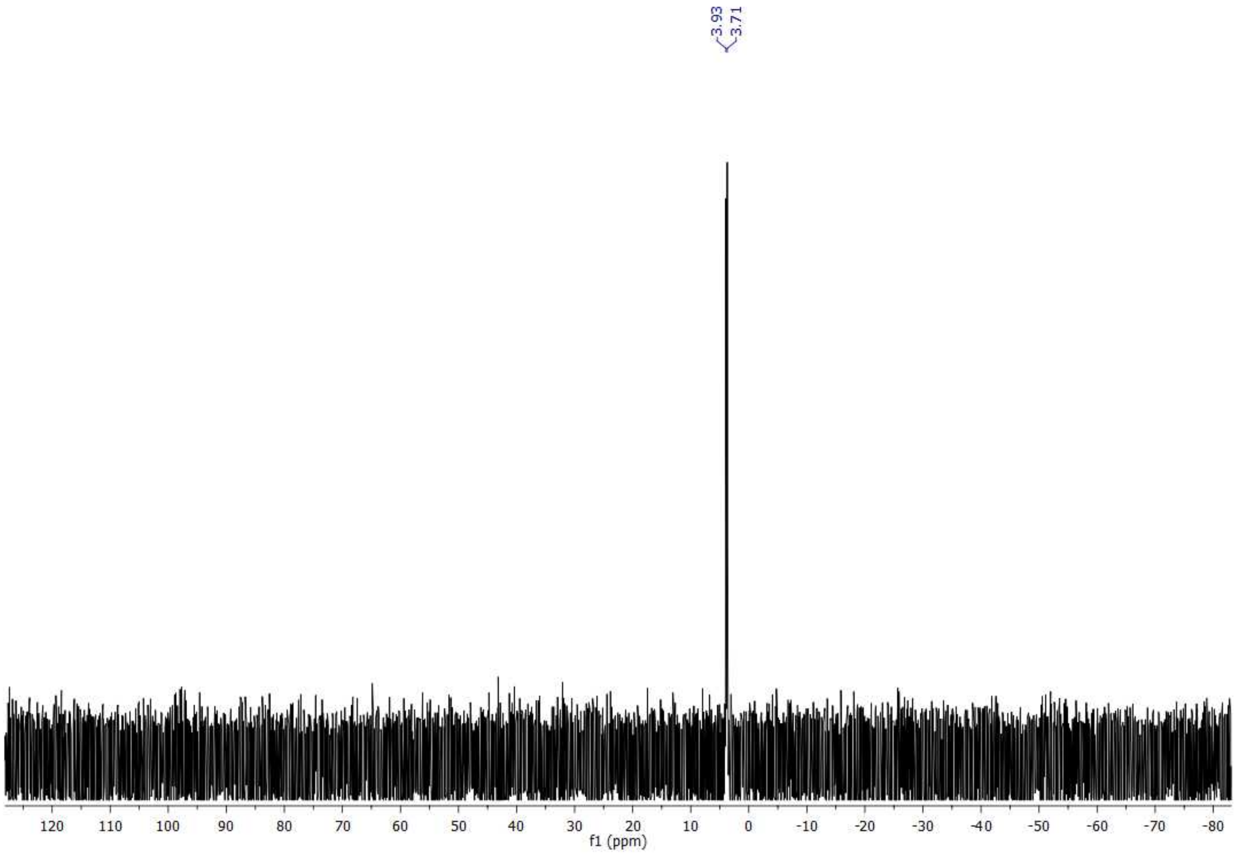

Supplementary Figure 45. ^{31}P NMR spectrum of 1-*epi*-valienol 1,7-diphosphate (202 MHz, D_2O).

Supplementary References

1 Paget, M. S., Chamberlin, L., Atrih, A., Foster, S. J. & Buttner, M. J. Evidence that the extracytoplasmic function sigma factor sigmaE is required for normal cell wall structure in *Streptomyces coelicolor* A3(2). *J. Bacteriol.* **181**, 204-211 (1999).
2 Ruckert, C. *et al.* Complete genome sequence of *Streptomyces lividans* TK24. *J. Biotechnol.* **199**, 21-22 (2015).
3 Cone, M. C., Yin, X., Grochowski, L. L., Parker, M. R. & Zabriskie, T. M. The blasticidin S biosynthesis gene cluster from *Streptomyces griseochromogenes*: sequence analysis, organization, and initial characterization. *ChemBioChem* **4**, 821-828 (2003).
4 Minagawa, K. *et al.* ValC, a new type of C_7-cyclitol kinase involved in the biosynthesis of the antifungal agent validamycin A. *ChemBioChem* **8**, 632-641 (2007).
5 Wehmeier, U. F. & Piepersberg, W. Biotechnology and molecular biology of the alpha-glucosidase inhibitor acarbose. *Appl. Microbiol. Biotechnol.* **63**, 613-625 (2004).
6 Wendler, S. *et al.* The cytosolic and extracellular proteomes of *Actinoplanes* sp. SE50/110 led to the identification of gene products involved in acarbose metabolism. *J. Biotechno.* **167**, 178-189 (2013).
7 Mahmud, T. *et al.* Biosynthetic studies on the alpha-glucosidase inhibitor acarbose in *Actinoplanes* sp.: 2-epi-5-epi-valiolone is the direct precursor of the valienamine moiety. *J. Am. Chem. Soc.* **121**, 6973-6983 (1999).
8 Yang, J. *et al.* Nucleotidylation of unsaturated carbasugar in validamycin biosynthesis. *Org. Biomol. Chem.* **9**, 438-449 (2011).
